# Synthesis and Biological Analysis of Iso-dimethyltryptamines in a Model of Light-Induced Retinal Degeneration

**DOI:** 10.1021/acsmedchemlett.4c00130

**Published:** 2024-06-13

**Authors:** Ethan J. Pazur, Anna Kalatanova, Nikhil R. Tasker, Katri Vainionpää, Henri Leinonen, Peter Wipf

**Affiliations:** † Department of Chemistry, University of Pittsburgh, Pittsburgh, Pennsylvania 15260, United States; ‡ Yliopistonrinne 3, Canthia, School of Pharmacy, University of Eastern Finland, 70211 Kuopio, Finland

**Keywords:** Serotonin Receptor Agonists, 5-HTR, Ergot Alkaloids, Iso-dimethyltryptamines, LIRD, Ophthalmology, Retinal Diseases

## Abstract

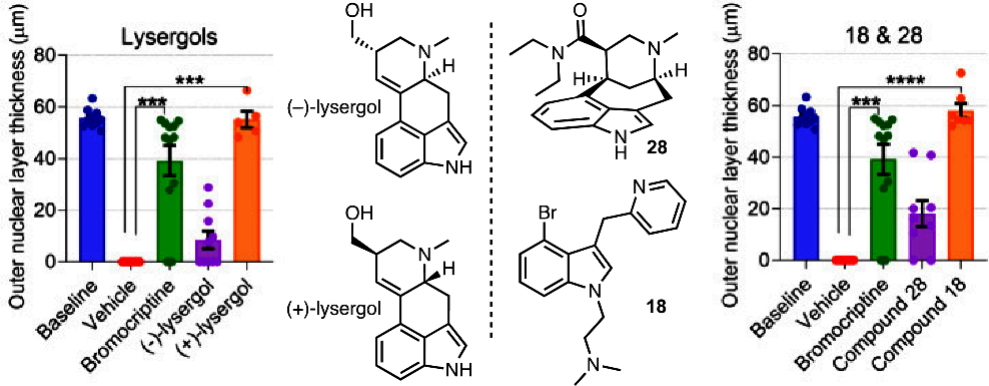

Iso-dimethyltryptamine (isoDMT) analogues with heterocyclic substitutions at the indole C(3) were prepared in a hydrogen autotransfer alkylation and tested in combination with natural and unnatural clavine alkaloids in a model of light-induced retinal degeneration for protection against retinal degeneration. On the basis of measurements with optical coherence tomography and electroretinography, three compounds showed better efficacy than the positive control bromocriptine at equivalent systemically administered doses. These studies provide further insights into the role of serotonin receptors and their potential therapeutic applications in ocular diseases.

The discovery of serotonin (5-hydroxytryptamine, 5-HT) receptors (5-HTRs) in retinal cells in the 1960s was followed by seminal studies that demonstrated that 5-HTR binding is involved in retinal pathology and photoreceptor survival.^1^ The *de novo* synthesis of 5-HT from tryptophan, activation of cAMP signaling pathways by binding to 5-HTRs, reuptake by 5-HT transporters, and 5-HT degradation by monoamine oxidases (MAO), are active processes in retina cells (Figure 1). A subset of retinal interneurons (amacrine cells) both synthesize and release 5-HT and, therefore, act as serotonergic neurons. 5-HTRs in retinal bipolar and ganglion cells are responsible for neuromodulation.^1−3^ Importantly, while many pharmacological studies have focused on the expression of 5-HTRs in animals, they are also expressed and play a neuroprotective role in human retina cells.^2,3^ However, the specific signaling pathways differ between cells and are influenced by off-target effects of the chemical probes utilized in earlier studies (Table 1).

**Figure 1:**
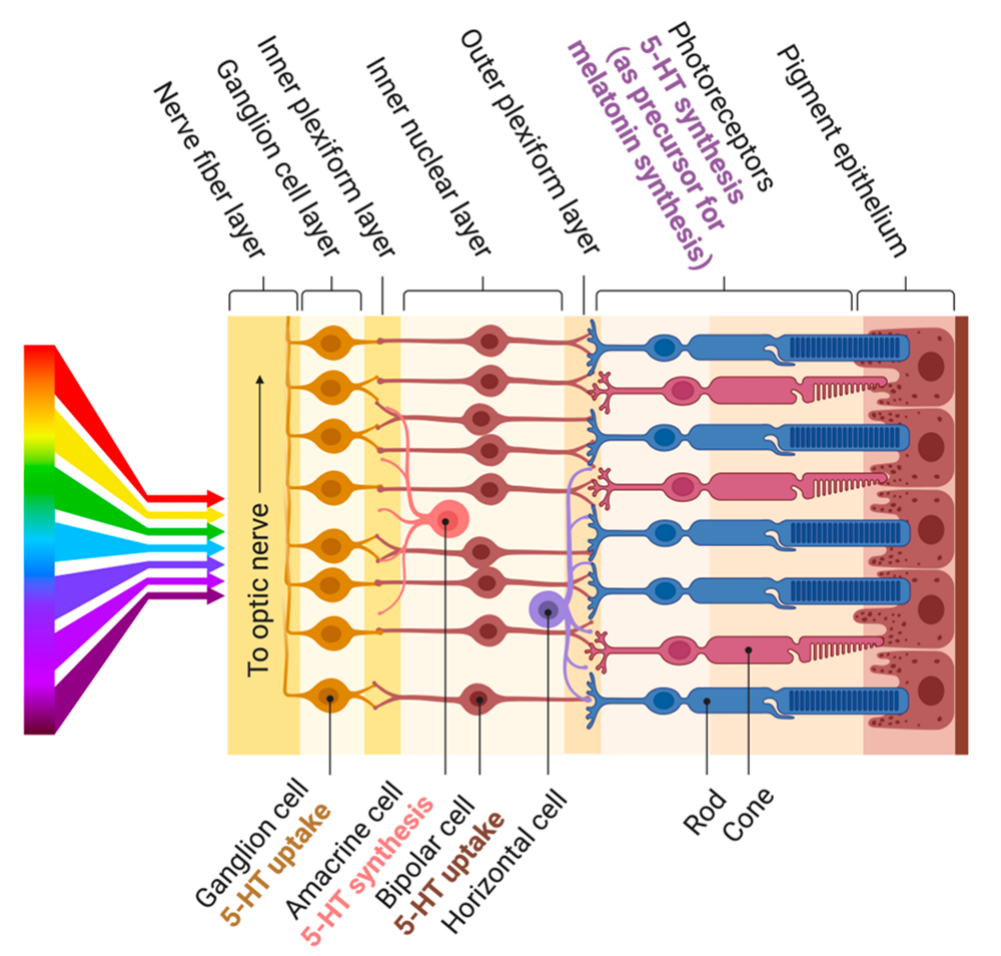
Serotonin (5-HT) synthesis and uptake in retina cells (created with BioRender).^1^

**Table 1:** 5-HTR Expression in Retina, Signaling Pathways, And Relevant Chemical Probes^1^

tissue or cell	5-HTR	activated signaling pathway	agonist/antagonist used
rabbit and goldfish retina	5-HT_1A_	increased cAMP	buspirone/spiroxatrine
culture human retinal pigment epithelium (RPE)	5-HT_1A_	decreased cAMP	buspirone/spiroxatrine
cultured rat RPE and retinal ganglion cells (RGC)	5-HT_2A,C_	increased inositol and Ca^2+^	5-HT/methysergide/spiperone/WAY-161503

Among the constitutively expressed 5-HT receptors, 5-HT_1A_, 5-HT_2A,B,C_, 5-HT_3A_, 5-HT_5A,B_, and 5-HT_7_ have been detected in the retina of various species, including humans.^1^ Activation of ocular 5-HT receptors was shown to rapidly initiate a CNS survival pathway and protect against injuries, such as severe photooxidative damage induced by exposure to blue light.^4^ 5-HT_1A_R agonists, such as buspirone, xaliproden, and 8-hydroxy-2-(di-*n*-propylamino)-tetralin (8-OH-DPAT), protect ARPE-19 human retinal pigment epithelium (RPE) cells against oxidative damage, as well as mouse RPE cells *in vivo* in geographic atrophy models,^5^ but currently there is only anecdotal evidence that links 5-HT_1A_R agonists to protection against light-induced retinal degeneration (LIRD).^6^ In contrast, this effect has been more thoroughly investigated in glaucoma. Activation of 5-HT_1A_R in the retina facilitates presynaptic γ-aminobutyric acid (GABA) release by suppressing cyclic adenosine monophosphate–protein kinase A (cAMP-PKA) signaling and decreasing PKA phosphorylation (Figure 2),^7^ which can explain the reduction of excitotoxicity in retinal ganglion cells (RGCs) during experimental glaucoma.^8^ Excessive cAMP signaling is also linked to inherited retinal degenerative diseases,^9^ and drugs that suppress cAMP show a remarkable therapeutic potential.^10^

**Figure 2:**
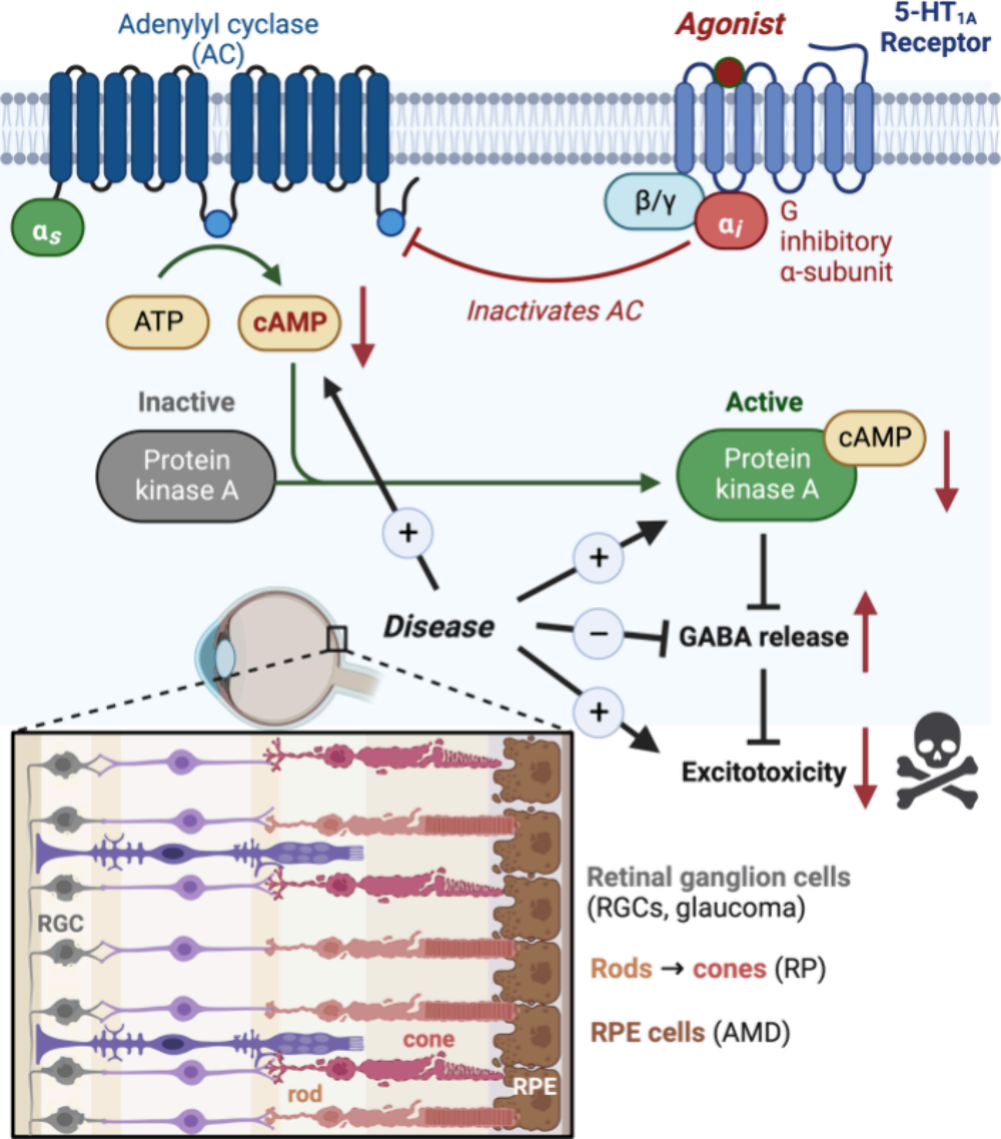
Mechanistic hypothesis for the neuroprotective effects of 5-HT_1A_R agonists. Cyclic adenosine monophosphate (cAMP) levels are elevated during retinal disease and are driving further degeneration.^9^ For instance, cAMP-activated PKA-mediated protein phosphorylation reduces GABA release, thereby causing hyperexcitability associated with glaucomatous damage. Agonists at 5-HT_1A_ inhibit this cascade by exchanging GDP for GTP on the α-subunit of Gi/o (Giα/Goα), thereby inhibiting adenylyl cyclase (AC) and resulting in decreased cAMP (created with BioRender).

Because of the insufficient subtype selectivity of the current generation of small molecule 5-HT modulators,^11^ it is not yet clear which 5-HTR among 5-HT_1A_, _2A_,_2B_ and 5-HT_2C_ receptors is mainly responsible for the protective effects of agonists, or if pan-activation is useful for therapeutic purposes. Furthermore, only limited information is currently available about the interplay of 5-HT_3A_, 5-HT_5A,B_, and 5-HT_7_ receptors in the retina. Literature data suggest that serotonergic 5-HT_1A_R activation in the retina is neuroprotective, whereas pan-5-HT_2_R activation might have a detrimental effect on retinal survival.^1−3^ In contrast, 5-HT_2_R agonists were found to be effective in reducing intraocular pressure (IOP) in a primate model of glaucoma,^12^ and 5-HT_2C_ regulates neurite growth and retinal processing of visual information.^13^ Sarpogrelate, a 5-HT_2A_/5-HT_2B_ antagonist, also proved protective in light-induced retinopathy.^14^ Significantly, 5-HTR agonists or antagonists have not yet been used in vision therapies, despite the large need for new treatment options in ocular diseases. Accordingly, we envisioned that specific 5-HTR probes will be useful to clarify receptor properties and identify new therapeutic opportunities.

Retinal degeneration is a common symptom of several blinding diseases, such as retinitis pigmentosa (RP)^15^ and age-related macular degeneration (AMD).^16^ Interestingly, RP is a leading cause of vision loss for people under the age of 55, while AMD is the leading cause of vision loss in the elderly.^17^ Both diseases are prevalent worldwide, and AMD is projected to be diagnosed in 288 million patients by 2040.^18^ Advanced AMD can be categorized as either “wet” or “dry” AMD with their differentiating characteristic being the abnormal formation and leakage of blood vessels.^17^ While dry AMD is much more common, available treatments remain limited. In fact, the FDA approved the first two drugs for treatment of dry AMD as recently as 2023.^19,20^ Treatments for RP have historically focused on attenuating symptoms, but new research efforts have leveraged gene therapy, which resulted in the first gene therapy to gain FDA approval for the eye.^21^

Constant and intense exposure to light is associated with photoreceptor cell death and ultimately retinal degeneration.^22,23^ This can lead to the acceleration of RP^24−26^ and is a risk factor for the development of AMD.^16,27,28^ Mechanistically, prolonged light exposure can disrupt the retinoid cycle by hindering the clearance of intermediates, such as all-*trans*-retinal, which results in its accumulation and eventual retinopathy.^29−32^ Build-up of all-*trans*-retinal may also lead to the formation of toxic byproducts that can cause additional damage to the retina.^33−37^ Furthermore, extended light exposure can result in the excessive production of reactive oxygen species (ROS) in the retina. Elevated ROS levels increase oxidative stress and activate apoptotic and proinflammatory pathways, both of which are contributing to retinal degeneration.^14,38−41^ There is also growing evidence that ROS play a significant role in the development and progression of glaucoma.^42^

Iso-dimethyltryptamines (isoDMTs) are potent 5-HTR agonists and have garnered considerable interest in recent years because of their potential as treatments for anxiety and depression with reduced hallucinogenic side effects (Figure 3).^43−45^ For example, AAZ-A-154 is a nonhallucinogen that has antidepressant properties similar to the drug ketamine.^45^ Structure–activity relationship (SAR) studies of the tryptamine scaffold typically include three major zones: the benzene ring of indole, the indole side chain, and the degree of alkylation at the terminal basic nitrogen.^43−51^ We have recently developed a high-yielding, robust indole C(3)-alkylation reaction that allows the installation of a pyridyl substituent at this position and offers opportunities to expand the SAR of isoDMTs.^52^

**Figure 3:**
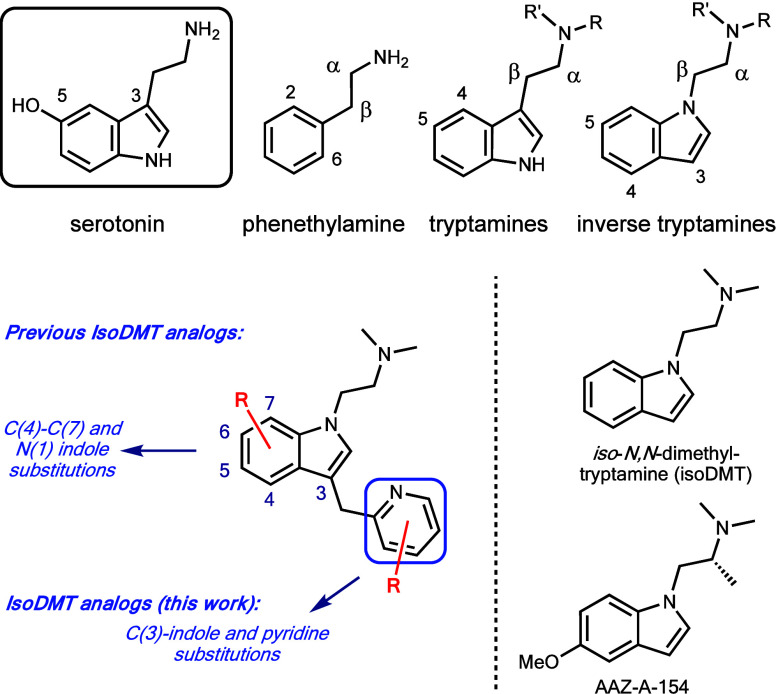
Examples of tryptamines, isoDMTs, and novel 3-methylpyridyl isoDMT analogues.

Specifically, pyridyl-substituted isoDMT analogues were synthesized following a two-step sequence (Scheme 1).^52,53^ Treatment of halo-, methoxy-, or aza-indoles **1**–**7** with 2- or 4-pyridinemethanol in the presence of oxone and Cs_2_CO_3_ in xylenes at reflux afforded the corresponding 3-substituted indoles **8**–**11**, **13**, and **16** and 2-aza-indole **12** in good to excellent yields. Hydrogen autotransfer (HA)-type alkylation of fluorinated indoles was also performed with 2-pyrimidinemethanol and 6-methyl-2-pyridinemethanol and provided the substituted indoles **14** and **17** in good yields. Furthermore, 5-fluoroindole (**2**) successfully reacted with 1-(2-pyridyl)ethanol to give **15** in 57% yield, which suggests that carbon chain branching at the benzylic position is possible. Subsequent treatment of **8**–**17** with 2-dimethylaminoethyl chloride hydrochloride, potassium hydroxide, and potassium iodide in DMSO at room temperature gave the *N*-alkylated isoDMT analogues **18** to **27** in 24% to 71% yield. With some substrates, oxidized side products were also isolated, which decreased the yields of the desired products. Although these side products were formed in relatively low amounts, they were difficult to remove chromatographically from the desired products. We explored changing solvent and base conditions to minimize side product formation, but these modifications resulted in sluggish reaction rates.

**Scheme 1:**
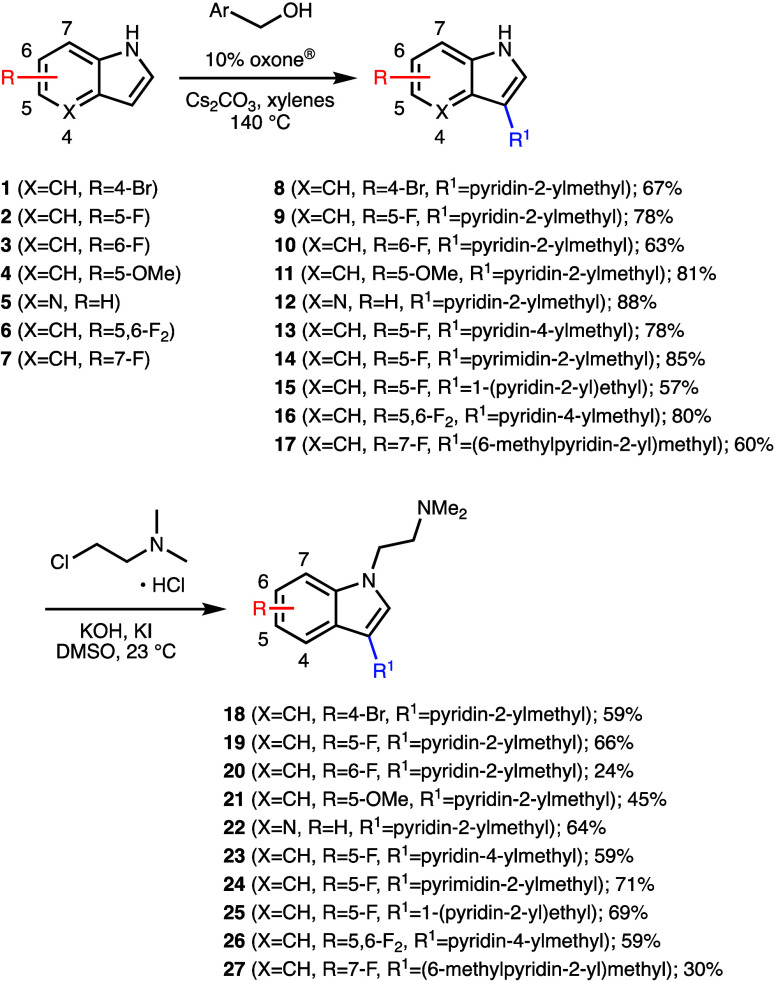
Synthesis of isoDMT Analogues by a Hydrogen Autotransfer (HA) Process Followed by *N*-Alkylation^52^

In order to obtain an assessment of the potential for these isoDMT derivatives to serve as lead structures for retinal degeneration therapeutics, we selected the halogenated analogues **18**–**20** for evaluation in a well-established model of light-induced retinal degeneration (LIRD).^54^ In addition to the isoDMTs, we also tested the protective effects of recently synthesized natural and unnatural clavine alkaloids that were demonstrated to have considerable 5-HTR subtype selectivity in this model (Figure 4).^55−57^ For example, (+)-cycloclavine was shown to possess ≥10-fold greater potency at 5-HT_2C_ versus 5-HT_1A/2A/2B_.^58^ In contrast, the bridged diethylamide **28** did not show any notable activity at 5-HT_1A,2A,2B,2C_.^59^

**Figure 4:**
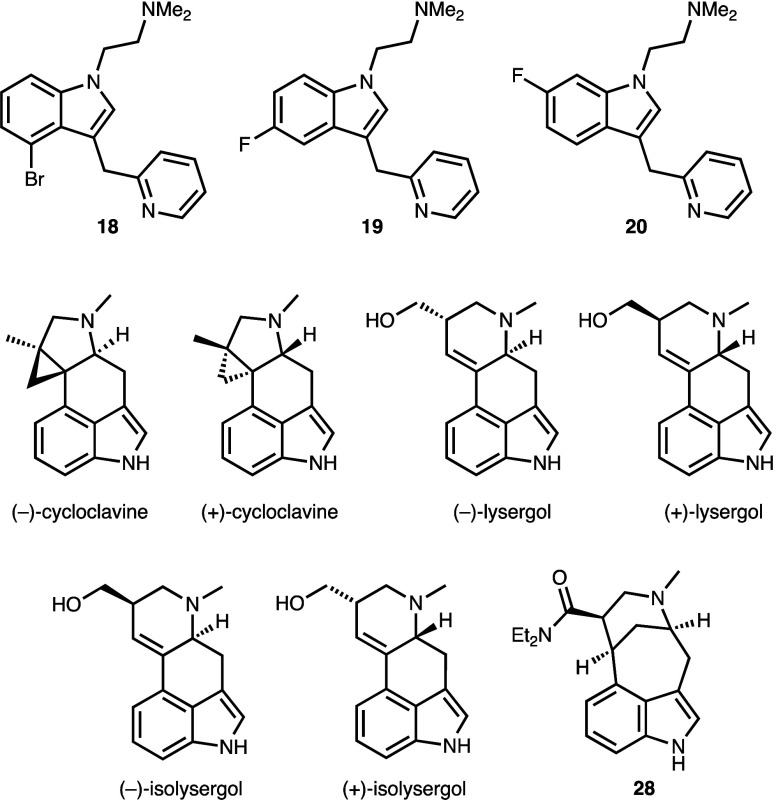
IsoDMT and clavine alkaloids selected for LIRD analysis.

BALB/c albino mice aged 5–8 weeks were used in the LIRD experiments. Mice were housed in a temperature-controlled animal facility with a 12 h light/dark cycle and fed a standard rodent diet *ad libitum*. All procedures were conducted in accordance with the Directive 86/609/EEC for animal experiments, Federation of European Laboratory Animal Science Associations (FELASA) guidelines and recommendations, and Association for Research in Vision and Ophthalmology (ARVO) Statement for the Use of Animals in Ophthalmic and Vision Research. Experiments were approved by the Finnish Project Authorization Board with protocol number ESAVI/26320/2021.

The isoDMTs and clavine alkaloids (Figure 4), or bromocriptine, were first individually dissolved in DMSO to generate 10 mg/mL stock solutions. On the day of LIRD induction, stock solutions were mixed with saline at a 1:4 ratio. The mice were dark-adapted overnight, and all handling prior to LIRD induction was performed under dim red light. Drugs [10 mg/kg of body weight (b.w.)] or vehicle (25% DMSO, 75% saline; volume adjusted to 100 μL) were intraperitoneally (ip) injected 30 min prior to the bright light exposure, and the mouse pupils were dilated using duplicate corneal administrations of metaoxedrin (20 mg/mL) and tropicamide (4 mg/mL) solution: first, at 30 min prior, and second, at 15 min prior to light exposure. LIRD was induced with a 30 min exposure to 15 kLux white light in freely moving mice (see schematic presentation of method in Supplementary Figure 1) and then transferred back to the vivarium. One week later, optical coherence tomography (OCT) imaging^60^ and electroretinography (ERG) recording^61^ were performed to assess retinal structure and function, respectively.

The method’s validity was confirmed since the retinas of all vehicle-treated mice showed severe LIRD (Figure 5; Supplementary Figure 2). As measured from the OCT images, the outer nuclear layer (ONL) thickness, a readout of mouse rod photoreceptor population, was reduced from a baseline mean at 55.6 to 0 μm as a result of LIRD. ERG a- and b-wave amplitudes, representing primarily rod photoreceptor and ONL bipolar cell population activation,^62^ respectively, were significantly attenuated across a large range of light intensities used for stimulation (Supplementary Figures 3 and 4). We used bromocriptine, an FDA-/EMA-approved semisynthetic ergot alkaloid drug, as a reference compound and positive control.^63^ Bromocriptine has been shown to possess therapeutic properties in multiple neuropathological contexts, including amytrophic lateral sclerosis (ALS) and Alzheimer′s disease.^64,65^ In our experiments, systemically administered bromocriptine (10 mg/kg) protected from ONL thinning by 70% (Figure 5) and ERG a- and b-wave amplitude (at 10 cd·s/m^2^ stimulus) deterioration also by 70% each (Figure 6). At equal dose, (+)-lysergol, (+)-isolysergol, and isoDMT **18** protected against LIRD on average better than bromocriptine did (Figures 5 and 6). In contrast, (−)-isolysergol was devoid of protective efficacy, and (−)-lysergol showed only a minute ERG amplitude improvement (Figure 6). Both (−)- and (+)-cycloclavines were approximately equally effective against LIRD as bromocriptine was, whereas **19** and **20** showed lower efficacy on average (Figures 5 and 6). Notably, the OCT data obtained from treatments with cycloclavines and compounds **19** and **20** showed high variance of responses to treatments: some mice displayed LIRD damage equal to vehicle-treated mice, whereas some mice were practically fully protected. The higher variance of responses with partially effective compounds, however, may be a characteristic of the LIRD model rather than arising from the compounds’ properties, per se.

**Figure 5:**
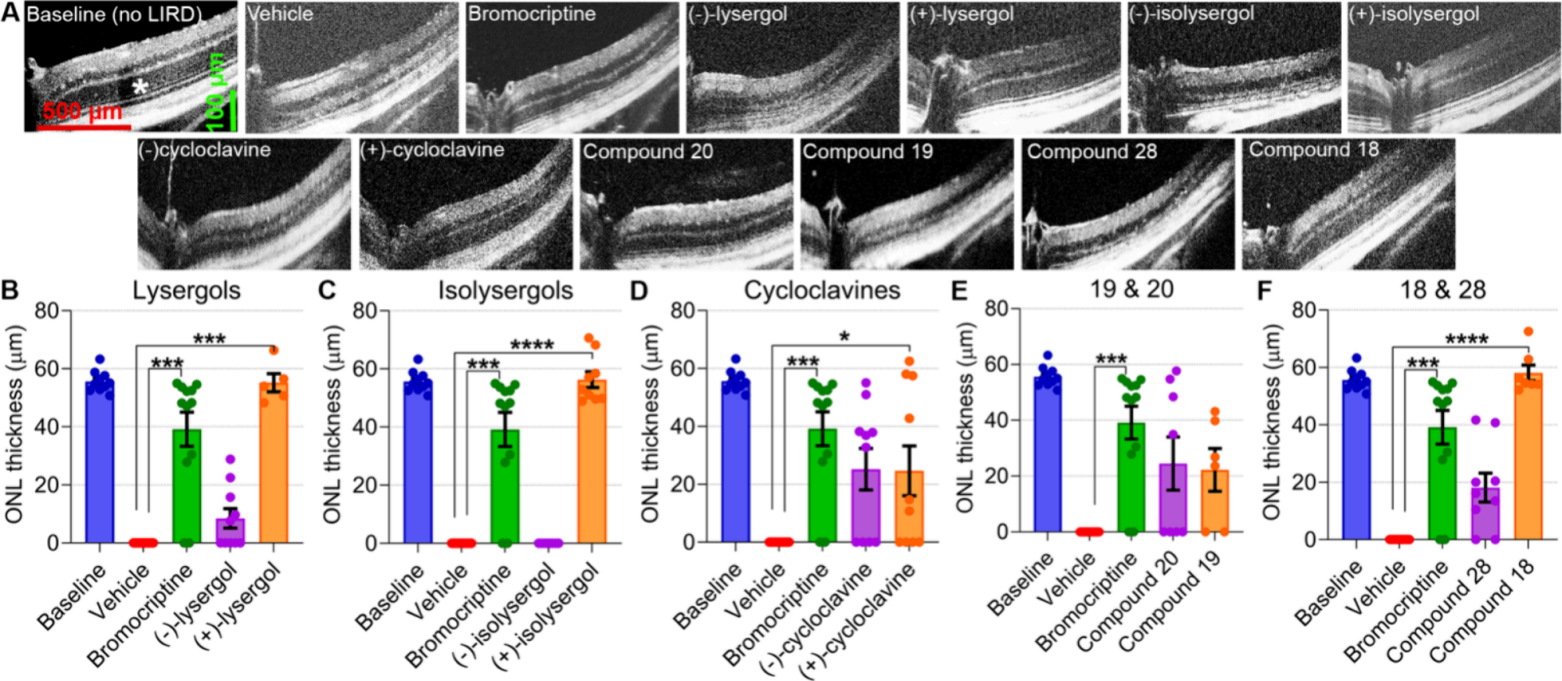
Systemically administered (+)-lysergol, (+)-isolysergol, and compound **18** display strong protection against LIRD-associated photoreceptor death. (A) Representative OCT images from each study group. Imaging was centered at the optic nerve head (ONH). ONL thickness was measured at 500 μm distance from the ONH border. Panels (B–F) display ONL thickness measurements (data averaged from superior, inferior, nasal, and temporal retinal quadrants) from experiments with lysergols (B), isolysergols (C), cycloclavines (D), **19** and **20** (E), and **18** and **28** (F) all contrasted with the data obtained from vehicle- and positive control (bromocriptine) treatments or baseline conditions (i.e., BALB/c mouse retinas without LIRD). The statistical analysis was performed using the nonparametric Kruskal–Wallis (K–W) test followed by Dunn’s tests for multiple comparisons. The asterisks signify results from the Dunn’s tests: *P < 0.05, ***P < 0.001, and ***P < 0.0001. Data is presented as mean ± SEM.

**Figure 6:**
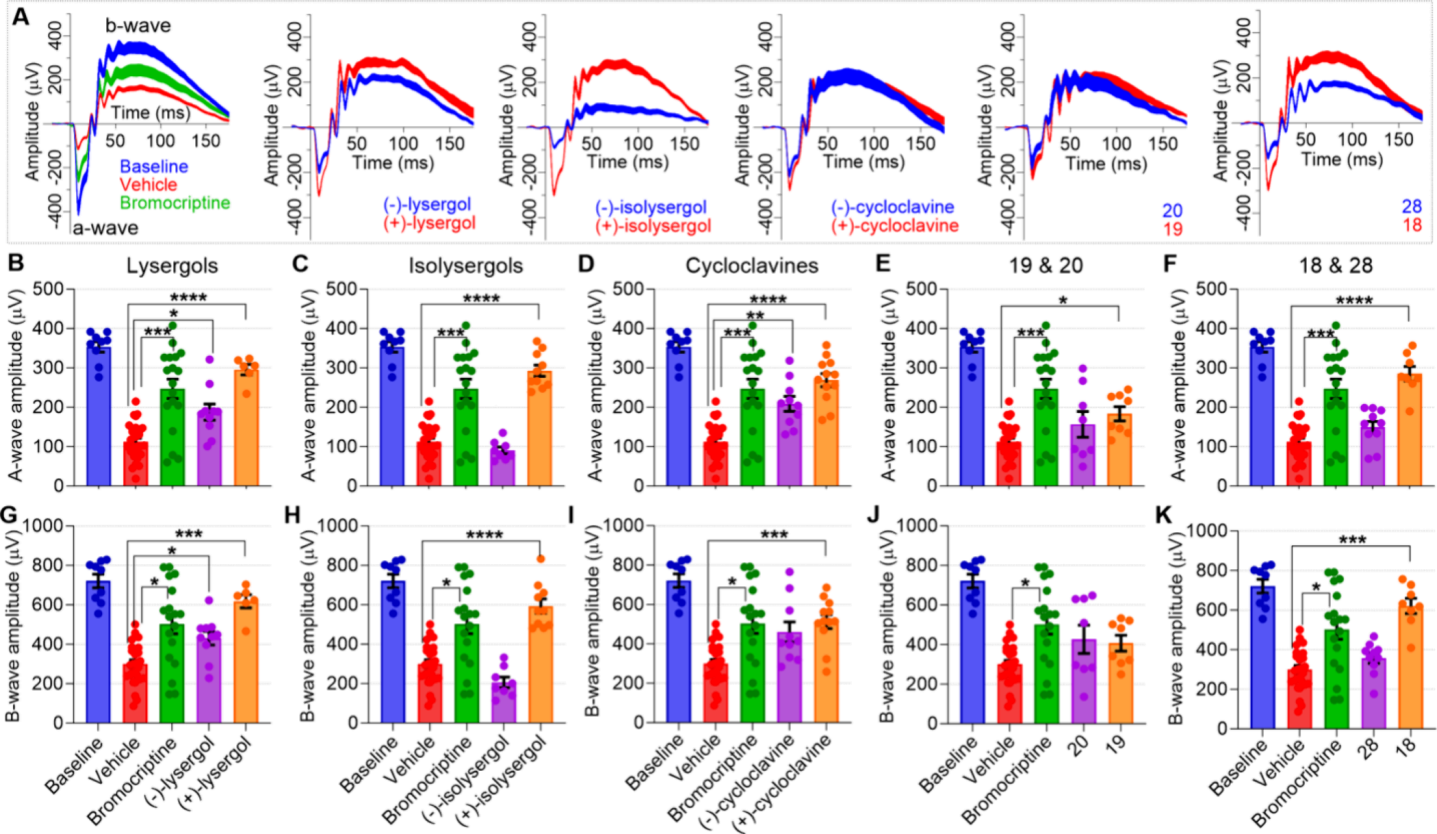
Retinal protection by (+)-lysergol, (+)-isolysergol, and **18** leads to near normal ERG responses 7 days after LIRD induction. For clarity, this figure presents ERG data from only 1 of 12 stimulation intensities used (10 cd·s/m^2^); full stimulus intensity–amplitude graphs are presented in Supplementary Figure 4. Panel (A) shows group-averaged ERG waveforms from all study groups. Panels (B–F) and (G–K) display ERG a- (B–F) and b-wave (G–K) amplitudes, respectively, from experiments with lysergols (B,G), isolysergols (C,H), cycloclavines (D,I), **19** and **20** (E,J), and **18** and **28** (F,K). The data of study compounds is contrasted with the data obtained from the treatments with vehicle- and positive control (bromocriptine) or at baseline conditions (no LIRD). The statistical analysis was performed using the Welch′s ANOVA test followed by Dunnett’s T3 post hoc tests. The asterisks signify results from the post hoc tests: *P < 0.05, **P < 0.01, ***P < 0.001, and ****P < 0.0001. Data is presented as mean ± SEM.

In summary, we have synthesized several new isoDMT analogues with heterocyclic substitutions at the indole C(3) and tested representative analogues in a proof-of-concept LIRD model for protection against retinal degeneration. The clavine alkaloids (+)-lysergol and (+)-isolysergol share strong agonistic activities at 5-HT_1A_R and 5-HT_2C_R^66^ and demonstrate a similar high level of protection in the LIRD model, whereas (−)-lysergol and (−)-isolysergol are both inactive in the LIRD model and at 5-HT_2C_R. However, (−)-isolysergol is quite potent as an agonist at 5-HT_1A_R.^66^ (+)-Lysergol is very potent at 5-HT_2A_R, but (+)-isolysergol and (−)- and (+)-cycloclavines lack potency at this receptor, as well as at 5-HT_2B_R, and have moderate to high potency at 5-HT_2C_R.^66^ The bridged scaffold **28** did not bind to 5-HT_1A,2A,2B,2C_ receptors and was also inactive in the LIRD assay. Accordingly, the data suggest that activity at 5-HT_2C_R likely drives the observed efficacy in the LIRD model. Interestingly, the structurally much simpler isoDMT **18** also demonstrates significant LIRD protective properties and, therefore, validates this scaffold for future investigations of its potential for therapeutic applications in retinal degeneration. Combined, these studies provide valuable insights into the role that serotonin receptors and their agonists play in ocular diseases and provide suitable lead compounds for further preclinical development.

## Supporting Information Available

Experimental details and ^1^H and ^13^C NMR spectra for new synthetic products and biological assay information (PDF)

## Abbreviations

5-HT: serotonin (5-hydroxytryptamine)
5-HTR: 5-hydroxytryptamine receptor
8-OH-DPAT: 8-hydroxy-2-(di-*n*-propylamino)-tetralin
ALS: amytrophic lateral sclerosis
AMD: age-related macular degeneration
ARPE-19: spontaneously arising retinal pigment epithelia cell line
cAMP: cyclic adenosine monophosphate
ARVO: Association for Research in Vision and Ophthalmology
BALB/c: Bagg albino c
DMT: dimethyltryptamine
ERG: electroretinography
FELASA: Federation of European Laboratory Animal Science Associations
GABA: γ-aminobutyric acid
GDP: guanosine diphosphate
GTP: guanosine triphosphate
HA: hydrogen autotransfer
IOP: intraocular pressure
isoDMT: iso-dimethyltryptamine
LIRD: light-induced retinal degeneration
MAO: monoamine oxidase
OCT: optical coherence tomography
ONL: outer nuclear layer
PKA: protein kinase A
RGC: retinal ganglion cell
ROS: reactive oxygen species
RP: retinitis pigmentosa
RPE: retinal pigment epithelium
SAR: structure–activity relationship
