# Supplementary material for: Synthesis and Biological Analysis of Iso-dimethyltryptamines in a Model of Light-Induced Retinal Degeneration
Source: ACS Med Chem Lett. 2024 Jun 13;15(7):1049–56. doi: 10.1021/acsmedchemlett.4c00130 (PMC11247652; doi:10.1021/acsmedchemlett.4c00130)

Supporting Information

Synthesis and Biological Analysis of *Iso*-Dimethyltryptamines in a Model of Light-Induced Retinal Degeneration

Ethan J. Pazur,[#,‡] Anna Kalatanova,[^,‡] Nikhil R. Tasker,[#] Katri Vainionpää,[^] Henri Leinonen,[^,*] and Peter Wipf[#,^,*]
[#]Department of Chemistry, University of Pittsburgh, Pittsburgh PA 15260, U.S.A.
[^]School of Pharmacy, University of Eastern Finland, 70211 Kuopio, Finland

henri.leinonen@uef.fi; pwipf@pitt.edu

Table of Contents

1. General Information ... 2
2. Procedures ... 3
2a. Synthesis and characterization of heteroaryl-substituted indoles. General Protocol A ... 3
2b. 5-Fluoro-3-(pyridin-4-ylmethyl)-1*H*-indole (**13**) ... 3
2c. 5-Fluoro-3-(pyrimidin-2-ylmethyl)-1*H*-indole (**14**) ... 3
2d. 5-Fluoro-3-(1-(pyridin-2-yl)ethyl)-1*H*-indole (**15**) ... 4
2e. 5,6-Difluoro-3-(pyridin-4-ylmethyl)-1*H*-indole (**16**) ... 5
2f. 7-Fluoro-3-((6-methylpyridin-2-yl)methyl)-1*H*-indole (**17**) ... 5
3. Synthesis and characterization of isoDMT analogs ... 6
3a. 2-(4-Bromo-3-(pyridin-2-ylmethyl)-1*H*-indol-1-yl)-*N*,*N*-dimethylethan-1-amine (**18**) ... 6
3b. 2-(5-Fluoro-3-(pyridin-2-ylmethyl)-1*H*-indol-1-yl)-*N*,*N*-dimethylethan-1-amine (**19**) ... 6
3c. 2-(6-Fluoro-3-(pyridin-2-ylmethyl)-1*H*-indol-1-yl)-*N*,*N*-dimethylethan-1-amine (**20**) ... 7
3d. 2-(5-Methoxy-3-(pyridin-2-ylmethyl)-1*H*-indol-1-yl)-*N*,*N*-dimethylethan-1-amine (**21**) ... 7
3e. *N*,*N*-Dimethyl-2-(3-(pyridin-2-ylmethyl)-1*H*-pyrrolo[3,2-*b*]pyridin-1-yl)ethan-1-amine (**22**) ... 8
3f. 2-(5-Fluoro-3-(pyridin-4-ylmethyl)-1*H*-indol-1-yl)-*N*,*N*-dimethylethan-1-amine (**23**) ... 9
3g. 2-(5-Fluoro-3-(pyrimidin-2-ylmethyl)-1*H*-indol-1-yl)-*N*,*N*-dimethylethan-1-amine (**24**) ... 10
3h. 2-(5-Fluoro-3-(1-(pyridin-2-yl)ethyl)-1*H*-indol-1-yl)-*N*,*N*-dimethylethan-1-amine (**25**) ... 10
3i. 2-(5,6-Difluoro-3-(pyridin-4-ylmethyl)-1*H*-indol-1-yl)-*N*,*N*-dimethylethan-1-amine (**26**) ... 11
3j. 2-(7-Fluoro-3-((6-methylpyridin-2-yl)methyl)-1*H*-indol-1-yl)-*N*,*N*-dimethylethan-1-amine (**27**) ... 12
Assays ... 13
Copies of NMR Spectra ... 19

1. General Information.

Unless stated otherwise, all reactions were performed under an atmosphere of N_2 gas that was passed through a column (10 x 2 cm) of Drierite®. Prior to use, Et_2O and THF were freshly distilled over sodium/benzophenone ketyl radical anion, and CH_2Cl_2 was freshly distilled over CaH_2. DMSO, DMF, MeCN were freshly distilled over CaH_2 and stored over 3 Å molecular sieves. For water- sensitive reactions, glassware and stir bars were dried in an oven at 140 °C for at least 16 h prior to use. Reactions were monitored by ^{1}H-NMR and TLC analysis (pre-coated silica gel 60 F254). TLC spots were visualized by UV light (254 nm and 395 nm), $KMnO_4$ stain, or a DNP stain. All NMR spectra were recorded on Bruker Avance 300 MHz, Bruker Avance 400 MHz, or Bruker Avance 500 MHz instruments. High resolution mass spectra were obtained on a Micromass UK Limited, Q-TOF Ultima API. Chemical shifts were reported in parts per million (ppm) with the residual solvent peak ($CDCl_3$: 7.26 ppm for ^{1}H, 77.16 ppm for ^{13}C; DMSO: 2.50 ppm for ^{1}H, 39.52 ppm for ^{13}C; D_2O: 4.79 ppm for ^{1}H) as the internal standard. Chemical shifts were tabulated as follows: chemical shift, multiplicity (s = singlet, d = doublet, t = triplet, q = quartet, quint = quintet, dd = doublet of doublets, dt = doublet of triplets, td = triplet of doublets, brs = broad singlet, m = multiplet), coupling constant(s), and integration. IR spectra were obtained using neat samples on a PerkinElmer 100 IR-ATR spectrometer. Temperature was monitored with a Chemglass Life Sciences high temperature (-10–260 °C) or a low temperature (-100–50 °C) thermometer. Melting points were obtained using a Mel-Temp instrument and are uncorrected. The products were mostly oils and air- and temperature-sensitive, making rigorous drying conditions difficult and leading, in some cases, to small residual solvent contamination. The syntheses and characterization data of compounds **8–12** have previously been described.[1]

The purity of final products was assessed using an Agilent Technologies 1260 Infinity II LC at 220 nm UV absorption (Waters XBridge BEH C_{18} 2.1 × 50 mm, 2.5 µm). All final assay samples showed a purity >95% by LCMS analysis with UV (220 and 254 nm) and TIC-MS detection. Compound **18** showed an additional peak at the LCMS solvent front, but passed QC based on the LCMS UV trace purity profile at 254 nm and NMR spectral analysis.

No unexpected or unusually high safety hazards were encountered.

[1] Pazur, E. J.; Tasker, N. R.; Wipf, P. "C3-Functionalization of Indoles with α-Heteroaryl-Substituted Methyl Alcohols." *Org. Biomol. Chem.* **2023**, *21*, 8651–8657.

2. Procedures

2a. Synthesis and characterization of heteroaryl-substituted indoles. General Protocol A.[1] A mixture of indole (1 equiv), pyridyl alcohol (3.0 equiv), Cs_2CO_3 (1.1 equiv), oxone® (0.10 equiv) in xylenes (2 M) in a sealed vessel was stirred at 140 °C under an atmosphere of N_2 until the starting material was completely consumed by TLC analysis. The reaction mixture was cooled to room temperature, and H_2O (10 mL) was added. The aqueous layer was extracted with CH_2Cl_2/MeOH (9:1, 3 x 15 mL) and the combined organic layers were dried ($MgSO_4$), filtered, and concentrated. The crude residue was purified by chromatography on SiO_2 (3% MeOH/CH_2Cl_2) to afford the desired product.

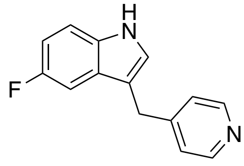

13

2b. 5-Fluoro-3-(pyridin-4-ylmethyl)-1*H*-indole (**13**). According to General Protocol A and using a reaction time of 24 h, 5-fluoroindole (0.500 g, 3.63 mmol) and 4-pyridinemethanol (1.20 g, 10.9 mmol) provided **13** (0.636 g, 2.81 mmol, 78%) as a tan solid: M.p. 141.4 °C (dec.); IR (ATR) ν_{max} 3047, 2911, 1606, 1584, 1487, 1464, 1418, 1434, 1354, 1233, 1219, 1180, 1149, 1101, 1045, 1001, 935, 912, 843, 818, 789, 764, 691 cm^{-1}; ^1H NMR (500 MHz, $CDCl_3$) δ 8.50 (d, J = 5.9 Hz, 2 H), 8.25 (brs, 1 H), 7.29 (dd, J = 8.8, 4.3 Hz, 1 H), 7.18 (d, J = 5.9 Hz, 2 H), 7.06 (dd, J = 9.5, 2.5 Hz, 1 H), 7.04 (d, J = 2.3 Hz, 1 H), 6.95 (td, J = 9.1, 2.5 Hz, 1 H), 4.07 (s, 2 H); ^{13}C NMR (125 MHz, $CDCl_3$) δ 158.0 (d, $J_{C,F}$ = 233.8 Hz), 150.2, 149.8, 133.1, 127.7 (d, $J_{C,F}$ = 9.4 Hz), 124.6, 124.1, 113.7 (d, $J_{C,F}$ = 5.0 Hz), 112.1 (d, $J_{C,F}$ = 9.5 Hz), 110.9 (d, $J_{C,F}$ = 26.3 Hz), 103.9 (d, $J_{C,F}$ = 23.1 Hz), 31.1; ^{19}F NMR (282 MHz, $CDCl_3$) δ –124.2; HRMS (ESI$^+$) *m/z* calcd for $C_{14}H_{12}N_2F$ ($[M+H]^+$) 227.0979, found 227.0981.

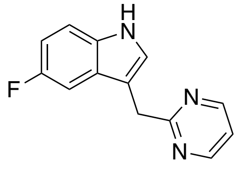

14

2c. 5-Fluoro-3-(pyrimidin-2-ylmethyl)-1*H*-indole (**14**). A mixture of 5-fluoroindole (0.500 g, 3.63 mmol), 2-pyrimidinemethanol (1.22 g, 10.9 mmol), Cs_2CO_3 (1.30 g, 3.99 mmol) and oxone® (0.223 g, 0.363 mmol) in xylenes (1.82 mL) in a sealed vessel was stirred under an atmosphere of N_2 at 140 °C for 24 h. The reaction mixture was cooled to room temperature, and H_2O/MeOH (1:1, 20 mL) was added.

The aqueous layer was extracted with CH_2Cl_2 (4 x 30 mL), and the combined organic layers were dried ($MgSO_4$), filtered through a celite plug, and concentrated. The crude residue was purified by reverse-phase chromatography on C18-SiO_2 (30 g, 10% MeCN/H_2O to 100% MeCN). Fractions containing the desired product were combined and extracted with CH_2Cl_2/MeOH (9:1, 3 x 30 mL). The combined organic extracts were dried ($MgSO_4$), filtered, and concentrated to afford **14** (0.698 g, 3.07 mmol, 85%) as a brown solid: M.p. 123.8 °C (dec.); IR (ATR) ν_{max} 3152, 2916, 1563, 1494, 1427, 1358, 1250, 1215, 1162, 1130, 1108, 1030, 1002, 936, 847, 793, 747, 760, 727, 697 cm^{-1}; ^{1}H NMR (400 MHz, $CDCl_3$) δ 8.69 (d, J = 4.9 Hz, 2 H), 8.12 (brs, 1 H), 7.30 (dd, J = 9.7, 2.5 Hz, 1 H), 7.24–7.21 (m, 2 H), 7.12 (t, J = 4.9 Hz, 1 H), 6.90 (td, J = 9.1, 2.5 Hz, 1 H), 4.40 (s, 2 H); ^{13}C NMR (100 MHz, $CDCl_3$) δ 170.2, 157.9 (d, $J_{C,F}$ = 233.0 Hz), 157.4, 133.0, 128.0 (d, $J_{C,F}$ = 10.0 Hz), 124.5, 118.8, 113.2 (d, $J_{C,F}$ = 4.6 Hz), 111.8 (d, $J_{C,F}$ = 9.6 Hz), 110.6 (d, $J_{C,F}$ = 26.4 Hz), 104.6 (d, $J_{C,F}$ = 23.5 Hz), 36.4; ^{19}F NMR (282 MHz, $CDCl_3$) δ –124.7; HRMS (ESI$^+$) m/z calcd for $C_{13}H_{11}N_3F$ ($[M+H]^+$) 228.0932, found 228.0933.

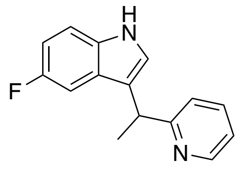

15

2d. 5-Fluoro-3-(1-(pyridin-2-yl)ethyl)-1*H*-indole (**15**). According to General Protocol A and using a reaction time of 48 h and 15% EtOAc/CH_2Cl_2 as eluent for purification by chromatography on SiO_2, 5-fluoroindole (0.200 g, 1.45 mmol) and 1-(pyridin-2-yl)ethanol (0.536 g, 4.35 mmol) gave **15** (0.197 g, 0.821 mmol, 57%) as a tan solid: M.p. 118.0 °C (dec.); IR (ATR) ν_{max} 3044, 2969, 2873, 1705, 1592, 1573, 1543, 1490, 1469, 1437, 1351, 1253, 1223, 1167, 1125, 1113, 1082, 1027, 933, 842, 806, 787, 748, 707 cm^{-1}; ^{1}H NMR (300 MHz, $CDCl_3$) δ 8.58 (ddd, J = 4.9, 1.7, 0.9 Hz, 1 H), 8.16 (brs, 1 H), 7.55 (td, J = 7.7, 1.8 Hz, 1 H), 7.23 (dd, J = 8.9, 4.5 Hz, 1 H), 7.16–7.08 (m, 3 H), 7.01 (dd, J = 9.8, 2.5 Hz, 1 H), 6.88 (td, J = 9.0, 2.5 Hz, 1 H), 4.46 (q, J = 7.1 Hz, 1 H), 1.75 (d, J = 7.2 Hz, 3 H); ^{13}C NMR (75 MHz, $CDCl_3$) δ 165.5, 157.7 (d, $J_{C,F}$ = 232.5 Hz), 149.2, 136.8, 133.3, 127.3 (d, $J_{C,F}$ = 9.6 Hz), 123.3, 121.9, 121.4, 120.2 (d, $J_{C,F}$ = 4.7 Hz), 111.8 (d, $J_{C,F}$ = 9.7 Hz), 110.5 (d, $J_{C,F}$ = 26.2 Hz), 104.6 (d, $J_{C,F}$ = 23.4 Hz) 39.6, 20.8; ^{19}F NMR (282 MHz, $CDCl_3$) δ –124.7; HRMS (ESI$^+$) m/z calcd for $C_{15}H_{14}N_2F$ ($[M+H]^+$) 241.1136, found 241.1127.

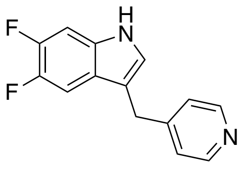

16

2e. 5,6-Difluoro-3-(pyridin-4-ylmethyl)-1*H*-indole (**16**). According to General Protocol A and using a reaction time of 24 h, 5,6-difluoroindole (0.500 g, 3.20 mmol) and 4-pyridinemethanol (1.06 g, 9.60 mmol) gave **16** (0.626 g, 2.56 mmol, 80%) as a greenish-blue solid: M.p. 148.6 °C (dec.); IR (ATR) ν_{max} 3128, 3077, 2997, 2919, 2856, 1603, 1557, 1476, 1446, 1419, 1352, 1340, 1201, 1146, 1099, 1032, 1003, 857, 846, 825, 789, 740 cm^{-1}; ^{1}H NMR (400 MHz, $CDCl_3$) δ 8.50 (dd, J = 4.6, 1.4 Hz, 2 H), 8.22 (brs, 1 H), 7.17–7.10 (m, 4 H), 7.01 (d, J = 2.0 Hz, 1 H), 4.04 (s, 2 H); ^{13}C NMR (125 MHz, $CDCl_3$) δ 150.0, 149.8, 148.5 (dd, $J_{C,F}$ = 221.3, 15.5 Hz), 146.6 (dd, $J_{C,F}$ = 217.6, 14.6 Hz), 131.6 (d, $J_{C,F}$ = 10.1 Hz), 124.1 (d, $J_{C,F}$ = 3.6 Hz), 124.0, 122.6 (d, $J_{C,F}$ = 7.2 Hz), 113.9 (dd, $J_{C,F}$ = 4.3, 1.9 Hz), 105.6 (d, $J_{C,F}$ = 18.9), 99.4 (d, $J_{C,F}$ = 21.6 Hz), 31.1; ^{19}F NMR (282 MHz, $CDCl_3$) δ –143.3 (d, $J_{F,F}$ = 19.7 Hz), –146.9 (d, $J_{F,F}$ = 19.7 Hz); HRMS (ESI$^+$) *m/z* calcd for $C_{14}H_{11}N_2F_2$ ($[M+H]^+$) 245.0885, found 245.0896.

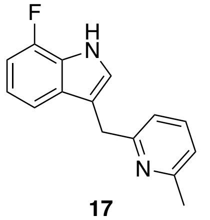

17

2f. 7-Fluoro-3-((6-methylpyridin-2-yl)methyl)-1*H*-indole (**17**). According to General Protocol A and using a reaction time of 144 h and 15% EtOAc/CH_2Cl_2 as eluent for purification by chromatography on SiO_2, 7-fluoroindole (0.200 g, 1.45 mmol) and 6-methyl-2-pyridinemethanol (0.547 g, 4.35 mmol) gave **17** (0.209 g, 0.868 mmol, 60%) as a light-tan solid: M.p. 162.5–163.7 °C; IR (ATR) ν_{max} 3013, 2951, 2924, 2779, 1638, 1600, 1576, 1502, 1459, 1432, 1351, 1220, 1108, 1046, 1004, 961, 803, 784, 756, 707, 668 cm^{-1}; ^{1}H NMR (500 MHz, $CDCl_3$) δ 8.25 (brs, 1 H), 7.42 (t, J = 7.7 Hz, 1 H), 7.29 (d, J = 7.9 Hz, 1 H), 7.08 (apparent d, J = 2.2 Hz, 1 H), 7.00–6.95 (m, 2 H), 6.91–6.87 (m, 2 H), 4.26 (s, 2 H), 2.57 (s, 3 H); ^{13}C NMR (125 MHz, $CDCl_3$) δ 160.3, 157.9 149.7 (d, $J_{C,F}$ = 242.1 Hz), 136.9, 131.4 (d, $J_{C,F}$ = 5.2 Hz), 124.9 (d, $J_{C,F}$ = 13.3 Hz), 123.4, 120.8, 119.8 (d, $J_{C,F}$ = 6.2 Hz), 119.7, 115.2 (d, $J_{C,F}$ = 3.4 Hz), 115.1 (d, $J_{C,F}$ = 2.0 Hz), 107.0 (d, $J_{C,F}$ = 15.7 Hz), 34.6, 24.7; ^{19}F NMR (282 MHz, $CDCl_3$) δ –135.6; HRMS (ESI$^+$) *m/z* calcd for $C_{15}H_{14}N_2F$ ($[M+H]^+$) 241.1136, found 241.1142.

3. Synthesis and characterization of isoDMT analogs

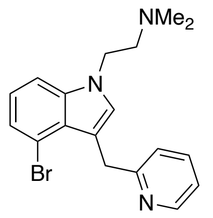

18

3a. 2-(4-Bromo-3-(pyridin-2-ylmethyl)-1*H*-indol-1-yl)-*N*,*N*-dimethylethan-1-amine (**18**). A mixture of **8** (0.100 g, 0.348 mmol), 2-dimethylaminoethyl chloride hydrochloride (0.0557 g, 0.383 mmol), KI (0.0642 g, 0.383 mmol), and KOH (0.115 g, 1.74 mmol) in DMSO (0.870 mL) was stirred at room temperature for 24 h. EtOAc (10 mL) was added, and the organic layer was washed with ice-cold brine (3 x 5 mL), dried ($MgSO_4$), filtered, and concentrated. The crude residue was purified by chromatography on SiO_2 (15% MeOH/EtOAc with 1% Et_3N) to afford **18** (0.0730 g, 0.204 mmol, 59%) as a brown oil: IR (ATR) ν_{max} 3360, 3061, 2972, 2941, 2860, 2821, 2769, 1606, 1591, 1568, 1548, 1473, 1434, 1397, 1357, 1329, 1311, 1266, 1246, 1156, 1113, 1095, 1049, 1037, 1021, 994, 946, 866, 832, 766, 735, 679 cm^{-1}; ^1H NMR (500 MHz, $CDCl_3$) δ 8.57 (d, J = 4.5 Hz, 1 H), 7.56 (td, J = 7.5, 1.5 Hz, 1 H), 7.34 (d, J = 8.2 Hz, 1 H), 7.25 (d, J = 8.2 Hz, 1 H), 7.13–7.11 (m, 2 H), 7.05 (t, J = 7.9 Hz, 1 H), 6.97 (s, 1 H), 4.58 (s, 2), 4.37 (brs, 2 H), 2.89 (brs, 2 H), 2.43 (brs, 6 H); ^{13}C NMR (125 MHz, $CDCl_3$) δ 162.0, 149.2, 137.8, 136.5, 128.9, 126.2, 123.9, 123.1, 122.7, 121.1, 114.8, 113.6, 108.8, 58.7, 45.6, 44.6, 35.0; HRMS (ESI^+) *m/z* calcd for $C_{18}H_{21}N_3Br$ ($[M+H]^+$) 358.0913, found 358.0915.

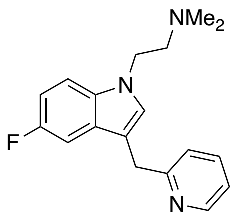

19

3b. 2-(5-Fluoro-3-(pyridin-2-ylmethyl)-1*H*-indol-1-yl)-*N*,*N*-dimethylethan-1-amine (**19**). A mixture of **9** (0.0612 g, 0.271 mmol), 2-dimethylaminoethyl chloride hydrochloride (0.0433 g, 0.298 mmol), KI (0.0499 g, 0.298 mmol), and KOH (0.0767 g, 1.35 mmol) in DMSO (0.678 mL) was stirred at room temperature for 24 h. Aqueous NaOH (1 M, 10 mL) was added, and the aqueous layer was extracted with CH_2Cl_2 (3 x 15 mL). The combined organic extracts were dried ($MgSO_4$), filtered, and concentrated. The crude residue was purified by chromatography on SiO_2 (10% MeOH/EtOAc with 1% Et_3N) to afford **19** (0.0529 g, 0.178 mmol, 66%) as a brown oil: IR (ATR) ν_{max} 3059, 2942, 2770, 1623, 1591, 1569, 1486,

1453, 1434, 1377, 1358, 1306, 1252, 1228, 1205, 1149, 1094, 1048, 994, 907, 851, 789, 767, 749, 695 cm^{-1}; ^{1}H NMR (300 MHz, $CDCl_3$) δ 8.54 (d, J = 4.8 Hz, 1 H), 7.54 (td, J = 7.7, 1.8 Hz, 1 H), 7.23 (dd, J = 7.4, 4.3 Hz, 1 H), 7.15–7.07 (m, 4 H), 6.92 (td, J = 9.1, 2.5 Hz, 1 H), 4.25–4.20 (m, 4 H), 2.75 (t, J = 7.2 Hz, 2 H), 2.33 (s, 6 H); ^{13}C NMR (75 MHz, $CDCl_3$) δ 161.0, 157.7 (d, $J_{C,F}$ = 232.9 Hz), 149.3, 136.6, 133.1, 128.3 (d, $J_{C,F}$ = 9.6 Hz), 128.1, 122.8, 121.3, 112.9 (d, $J_{C,F}$ = 4.8 Hz), 110.2 (d, $J_{C,F}$ = 25.6 Hz), 110.0 (d, $J_{C,F}$ = 9.1 Hz), 104.5 (d, $J_{C,F}$ = 23.2 Hz), 58.8, 45.6, 44.5, 34.5; ^{19}F NMR (282 MHz, $CDCl_3$) δ –125.3; HRMS (ESI$^+$) *m/z* calcd for $C_{18}H_{21}N_3F$ ([M+H]$^+$) 298.1714, found 298.1708.

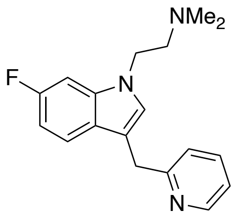

20

3c. 2-(6-Fluoro-3-(pyridin-2-ylmethyl)-1*H*-indol-1-yl)-*N,N*-dimethylethan-1-amine (**20**). A mixture of **10** (0.0300 g, 0.133 mmol), 2-dimethylaminoethyl chloride hydrochloride (0.0212 g, 0.146 mmol), KI (0.0245 g, 0.146 mmol), and KOH (0.0372 g, 0.663 mmol) in DMSO (0.333 mL) was stirred at room temperature for 24 h. Aqueous NaOH (1 M, 10 mL) was added, and the aqueous layer was extracted with CH_2Cl_2 (3 x 15 mL). The combined organic extracts were dried ($MgSO_4$), filtered, and concentrated. The crude residue was purified by chromatography on SiO_2 (10% MeOH/EtOAc with 1% Et_3N) to afford **20** (0.00940 g, 0.0316 mmol, 24%) as a brown oil: IR (ATR) ν_{max} 3058, 2945, 2772, 1620, 1591, 1568, 1472, 1435, 1374, 1335, 1255, 1219, 1146, 1115, 1094, 1049, 1017, 995, 922, 895, 825, 800, 749, 707, 670 cm^{-1}; ^{1}H NMR (500 MHz, $CDCl_3$) δ 8.55 (d, J = 4.4 Hz, 1 H), 7.54 (td, J = 7.7, 1.8 Hz, 1 H), 7.38 (dd, J = 8.7, 5.4 Hz, 1 H), 7.14 (d, J = 7.9 Hz, 1 H), 7.10 (dd, J = 7.3, 5.1 Hz 1 H), 6.99–6.97 (m with apparent singlet at 6.99, 2 H), 6.80 (td, J = 8.9, 2.3 Hz, 1 H), 4.24 (s, 2 H), 4.15 (t, J = 7.1 Hz, 2 H), 2.71 (t, J = 7.2 Hz, 2 H), 2.31 (s, 6 H); ^{13}C NMR (125 MHz, $CDCl_3$) δ 161.2, 160.1 (d, $J_{C,F}$ = 236.0 Hz), 149.3, 136.7 (d, $J_{C,F}$ = 11.5 Hz), 136.6, 126.9 (d, $J_{C,F}$ = 3.6 Hz), 124.6, 122.8, 121.3, 120.4 (d, $J_{C,F}$ = 10.1 Hz), 113.2, 107.9 (d, $J_{C,F}$ = 24.4 Hz), 95.8 (d, $J_{C,F}$ = 26.1 Hz), 58.8, 45.8, 44.7, 34.6; ^{19}F NMR (282 MHz, $CDCl_3$) δ –120.9; HRMS (ESI$^+$) *m/z* calcd for $C_{18}H_{21}N_3F$ ([M+H]$^+$) 298.1714, found 298.1705.

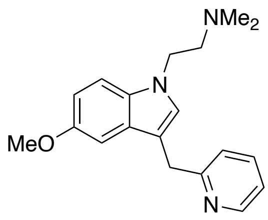

21

3d. 2-(5-Methoxy-3-(pyridin-2-ylmethyl)-1*H*-indol-1-yl)-*N*,*N*-dimethylethan-1-amine (**21**). A mixture of **11** (0.0500 g, 0.210 mmol), 2-dimethylaminoethyl chloride hydrochloride (0.0336 g, 0.231 mmol), KI (0.0387 g, 0.231 mmol), and KOH (0.0595 g, 1.05 mmol) in DMSO (0.525 mL) was stirred at room temperature for 24 h. Aqueous NaOH (1 M, 10 mL) was added, and the aqueous layer was extracted with CH_2Cl_2 (3 x 15 mL). The combined organic extracts were dried ($MgSO_4$), filtered, and concentrated. The crude residue was purified by chromatography on SiO_2 (10% MeOH/EtOAc with 1% Et_3N) to afford **21** (0.0295 g, 0.0953 mmol, 45%) as a brown oil: IR (ATR) ν_{max} 3415, 2940, 2824, 2769, 1621, 1591, 1568, 1486, 1452, 1434, 1221, 1153, 1100, 1038, 994, 895, 835, 768, 788, 751, 692cm^{-1}; ^{1}H NMR (300 MHz, $CDCl_3$) δ 8.54 (d, *J* = 4.1 Hz, 1 H), 7.52 (td, *J* = 7.7, 1.8 Hz, 1 H), 7.21 (d, *J* = 8.8 Hz, 1 H), 7.14 (d, *J* = 7.8 Hz, 1 H), 7.08 (dd, *J* = 7.2, 5.0 Hz, 1 H), 7.00 (s, 1 H), 6.93 (d, *J* = 2.3 Hz, 1 H), 6.85 (dd, *J* = 8.8, 2.4 Hz 1 H), 4.25—4.21 (m, 4 H), 3.77 (s, 3 H), 2.77 (t, *J* = 7.2 Hz, 2 H), 2.34 (s, 6 H); ^{13}C NMR (75 MHz, $CDCl_3$) δ 161.3, 153.9, 149.1, 136.5, 131.7, 128.4, 127.1, 122.8, 121.1, 112.5, 112.0, 110.0, 101.4, 58.7, 55.9, 45.4, 44.3, 34.6; HRMS (ESI^{+}) *m/z* calcd for $C_{19}H_{24}ON_3$ ([M+H]$^{+}$) 310.1914, found 310.1909.

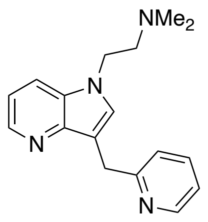

22

3e. *N*,*N*-Dimethyl-2-(3-(pyridin-2-ylmethyl)-1*H*-pyrrolo[3,2-*b*]pyridin-1-yl)ethan-1-amine (**22**). A mixture of **12** (0.100 g, 0.478 mmol), 2-dimethylaminoethyl chloride hydrochloride (0.0765 g, 0.526 mmol), KI (0.0873 g, 0.526 mmol), and KOH (0.134 g, 2.39 mmol) in DMSO (1.20 mL) was stirred at room temperature for 24 h. The supernatant was removed and directly purified by reverse-phase chromatography on C18-SiO_2 (12 g, 10% MeCN/H_2O to 100% MeCN). Fractions containing the desired product were combined, diluted with H_2O (10 mL), and extracted with CH_2Cl_2 (4 x 30 mL). The combined organic extracts were dried ($MgSO_4$), filtered, and concentrated to afford **22** (0.0861 g, 0.307 mmol, 64%) as a brown oil: IR (ATR) ν_{max} 3376, 2941, 2770, 1719, 1607, 1591, 1568, 1430, 1400, 1342, 1320, 1305, 1153, 1040, 767, 661 cm^{-1}; ^{1}H NMR (400 MHz, $CDCl_3$) δ 8.54 (dd, *J* = 4.9, 0.8 Hz, 1

H), 8.47 (dd, J = 4.6, 1.3 Hz, 1 H), 7.62 (dd, J = 8.2, 1.2 Hz, 1 H), 7.55 (td, J = 7.7, 1.8 Hz, 1 H), 7.35 (d, J = 7.8 Hz, 1 H), 7.22 (s, 1 H), 7.13–7.08 (m, 2 H), 4.40 (s, 2 H), 4.17 (t, J = 7.1 Hz, 2 H), 2.67 (t, J = 7.2 Hz, 2 H), 2.28 (s, 6 H); ^{13}C NMR (100 MHz, $CDCl_3$) δ 161.4, 149.2, 145.5, 142.8, 136.6, 129.8, 129.5, 123.4, 121.2, 116.5, 116.4, 113.9, 59.2, 45.8, 44.9, 33.0; HRMS (ESI$^+$) *m/z* calcd for $C_{17}H_{21}N_4$ ([M+H]$^+$) 281.1761, found 281.1761.

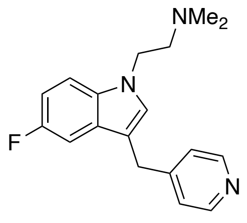

23

3f. 2-(5-Fluoro-3-(pyridin-4-ylmethyl)-1*H*-indol-1-yl)-*N*,*N*-dimethylethan-1-amine (**23**). A mixture of **13** (0.100 g, 0.442 mmol), 2-dimethylaminoethyl chloride hydrochloride (0.0707 g, 0.486 mmol), KI (0.0815 g, 0.486 mmol), and KOH (0.124 g, 2.21 mmol) in DMSO (1.11 mL) was stirred at room temperature for 24 h. EtOAc (10 mL) was added, and the organic layer was washed with ice-cold brine (3 x 5 mL). The combined aqueous layers were extracted with EtOAc (10 mL), and the combined organic layers were dried ($MgSO_4$), filtered, and concentrated. The crude residue was purified by reverse-phase chromatography on C18-SiO_2 (12 g, 10% MeCN/H_2O to 100% MeCN). Fractions containing the desired product were combined, diluted with H_2O (10 mL), and extracted with CH_2Cl_2 (4 x 30 mL). The combined organic extracts were dried ($MgSO_4$), filtered, and concentrated to afford **23** (0.0778 g, 0.262 mmol, 59%) as a brown oil: IR (ATR) ν_{max} 2943, 2823, 2770, 1710m 1623, 1600, 1560, 1487, 1453, 1415, 1378, 1358, 1309, 1219, 1149, 1095, 1035, 933, 907, 848, 790, 752, 731, 693 cm^{-1}; ^{1}H NMR (300 MHz, $CDCl_3$) δ 8.48 (d, J = 4.8 Hz, 2 H), 7.24 (dd, J = 8.9, 4.2 Hz, 1 H), 7.16 (d, J = 5.8, Hz, 2 H), 7.04 (dd, J = 9.5, 2.3 Hz, 1 H), 6.98–6.92 (m with apparent singlet at 6.96, 2 H), 4.17 (t, J = 7.0 Hz, 2 H), 4.03 (s, 2 H), 2.67 (t, J = 7.2 Hz, 2 H), 2.28 (s, 6 H); ^{13}C NMR (75 MHz, $CDCl_3$) δ 157.8 (d, $J_{C,F}$ = 233.4 Hz), 150.2, 150.0, 133.2, 128.3, 128.0 (d, $J_{C,F}$ = 9.6 Hz), 124.0, 112.2 (d, $J_{C,F}$ = 4.8 Hz), 110.4 (d, $J_{C,F}$ = 22.7 Hz), 110.1 (d, $J_{C,F}$ = 6.1 Hz), 104.1 (d, $J_{C,F}$ = 23.1 Hz), 59.1, 45.9, 45.0, 31.0; ^{19}F NMR (282 MHz, $CDCl_3$) δ –124.9; HRMS (ESI$^+$) *m/z* calcd for $C_{18}H_{21}N_3F$ ([M+H]$^+$) 298.1714, found 298.1705.

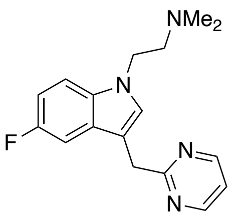

24

3g. 2-(5-Fluoro-3-(pyrimidin-2-ylmethyl)-1*H*-indol-1-yl)-*N*,*N*-dimethylethan-1-amine (**24**). A mixture of **14** (0.200 g, 0.880 mmol), 2-dimethylaminoethyl chloride hydrochloride (0.141 g, 0.968 mmol), KI (0.162 g, 0.968 mmol), and KOH (0.247 g, 4.40 mmol) in DMSO (2.20 mL) was stirred at room temperature for 24 h. The supernatant was removed and directly purified by reverse-phase chromatography on C18-SiO_2 (12 g, 10% MeCN/H_2O to 100% MeCN). Fractions containing the desired product were combined, diluted with H_2O (10 mL), and extracted with CH_2Cl_2 (4 x 30 mL). The combined organic extracts were dried ($MgSO_4$), filtered, and concentrated to afford **24** (0.186 g, 0.623 mmol, 71%) as a brown solid: M.p. 64.8–66.8 °C; IR (ATR) ν_{max} 3402, 2944, 2825, 2773, 1561, 1487, 1453, 1420, 1173, 1150, 1096, 1059, 1036, 928, 906, 895, 853, 788, 696 cm^{-1}; ^1H NMR (300 MHz, $CDCl_3$) δ 8.67 (d, J = 4.9 Hz, 2 H), 7.28 (dd, J = 9.7, 2.4 Hz, 1 H), 7.21–7.17 (m with apparent singlet at 7.17, 2 H), 7.10 (t, J = 4.9 Hz, 1 H), 6.91 (td, J = 9.1, 2.5 Hz, 1 H) 4.36 (s, 2 H), 4.15 (t, J = 7.1 Hz, 2 H), 2.68 (t, J = 7.3 Hz, 2 H), 2.29 (s, 6 H); ^{13}C NMR (75 MHz, $CDCl_3$) δ 170.3, 157.7 (d, $J_{C,F}$ = 232.4 Hz), 157.4, 133.2, 128.3 (d, $J_{C,F}$ = 9.8 Hz), 128.2, 118.7, 111.6 (d, $J_{C,F}$ = 4.8 Hz), 110.0 (d, $J_{C,F}$ = 32.4 Hz) 109.9 (d, $J_{C,F}$ = 3.5 Hz), 104.8 (d, $J_{C,F}$ = 23.3 Hz), 59.1, 45.9, 44.9, 36.4; ^{19}F NMR (282 MHz, $CDCl_3$) δ –125.4; HRMS (ESI^+) *m/z* calcd for $C_{17}H_{20}N_4F$ ($[M+H]^+$) 299.1667, found 299.1667.

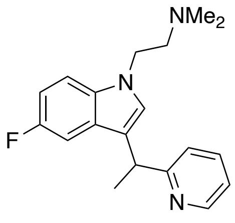

25

3h. 2-(5-Fluoro-3-(1-(pyridin-2-yl)ethyl)-1*H*-indol-1-yl)-*N*,*N*-dimethylethan-1-amine (**25**). A mixture of **15** (0.100 g, 0.416 mmol), 2-dimethylaminoethyl chloride hydrochloride (0.0666 g, 0.458 mmol), KI (0.0768 g, 0.458 mmol), and KOH (0.117 g, 2.08 mmol) in DMSO (1.04 mL) was stirred at room temperature for 24 h. EtOAc (10 mL) was added, and the organic layer was washed with ice-cold brine (3 x 5 mL). The combined aqueous layers were extracted with EtOAc (10 mL), and the combined organic layers were dried ($MgSO_4$), filtered, and concentrated. The crude residue was purified by reverse-phase chromatography on C18-SiO_2 (12 g, 10% MeCN/H_2O to 100% MeCN). Fractions containing the desired

product were combined, diluted with H_2O (10 mL), and extracted with CH_2Cl_2 (4 x 30 mL). The combined organic extracts were dried ($MgSO_4$), filtered, and concentrated to afford **25** (0.0895 g, 0.287 mmol, 69%) as a yellow oil: IR (ATR) ν_{max} 3383, 2937, 2871, 2823, 2772, 1640, 1622, 1590, 1569, 1485, 1452, 1432, 1365, 1274, 1220, 1182, 1150, 1097, 1043, 931, 898, 883, 850, 787, 750, 710 cm^{-1}; ^{1}H NMR (300 MHz, $CDCl_3$) δ 8.56 (dd, *J* = 4.8, 0.9 Hz, 1 H), 7.53 (td, *J* = 7.7, 1.8 Hz, 1 H), 7.19 (dd, *J* = 8.9, 4.3 Hz, 1 H), 7.14–7.06 (m with apparent singlet at 7.10, 3 H), 6.99 (dd, *J* = 9.8, 2.4 Hz, 1 H), 6.89 (td, *J* = 9.1, 2.5 Hz, 1 H), 4.44 (q, *J* =7.1 Hz, 1 H), 4.18 (t, *J* = 7.1 Hz, 2 H), 2.70 (t, *J* = 7.4 Hz, 2 H), 2.30 (s, 6 H), 1.73 (d, *J* = 7.2 Hz, 3 H); ^{13}C NMR (75 MHz, $CDCl_3$) δ 165.5, 157.5 (d, $J_{C,F}$ = 232.6 Hz), 149.3, 136.6, 133.3, 127.7 (d, $J_{C,F}$ = 9.7 Hz), 126.9, 121.8, 121.3, 118.9 (d, $J_{C,F}$ = 4.8 Hz), 110.1 (d, $J_{C,F}$ = 18.9 Hz), 109.8 (d, $J_{C,F}$ = 2.3 Hz), 104.8 (d, $J_{C,F}$ = 23.3 Hz), 59.1, 45.9, 45.1, 39.5, 20.9; ^{19}F NMR (282 MHz, $CDCl_3$) δ –125.4; HRMS (ESI$^+$) *m/z* calcd for $C_{19}H_{23}N_3F$ ([M+H]$^+$) 312.1871, found 312.1860.

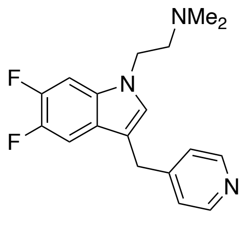

26

3i. 2-(5,6-Difluoro-3-(pyridin-4-ylmethyl)-1*H*-indol-1-yl)-*N*,*N*-dimethylethan-1-amine (**26**). A mixture of **16** (0.100 g, 0.409 mmol), 2-dimethylaminoethyl chloride hydrochloride (0.0655 g, 0.450 mmol), KI (0.0755 g, 0.450 mmol), and KOH (0.115 g, 2.05 mmol) in DMSO (1.02 mL) was stirred at room temperature for 24 h. EtOAc (10 mL) was added, and the organic layer was washed with ice-cold brine (3 x 5 mL). The combined aqueous layers were extracted with EtOAc (10 mL), and the combined organic layers were dried ($MgSO_4$), filtered, and concentrated. The crude residue was purified by reverse-phase chromatography on C18-SiO_2 (12 g, 10% MeCN/H_2O to 100% MeCN). Fractions containing the desired product were combined, diluted with H_2O (10 mL), and extracted with CH_2Cl_2 (4 x 30 mL). The combined organic extracts were dried ($MgSO_4$), filtered, and concentrated to afford **26** (0.0777 g, 0.243 mmol, 59%) as a brown oil: IR (ATR) ν_{max} 2943, 2823, 2772, 1600, 1560, 1523, 1487, 1475, 1414, 1386, 1337, 1288, 1246, 1208, 1141, 1089, 1034, 994, 938, 844, 798, 752, 673 cm^{-1}; ^{1}H NMR (500 MHz, $CDCl_3$) δ 8.49 (d, *J* = 5.8 Hz, 2 H), 7.14 (d, *J* = 5.9 Hz, 2 H), 7.12–7.08 (m, 2 H), 6.94 (s, 1 H), 4.10 (t, *J* = 7.0 Hz, 2 H), 4.01 (s, 2 H), 2.65 (t, *J* = 7.0 Hz, 2 H), 2.28 (s, 6 H); ^{13}C NMR (125 MHz, $CDCl_3$) δ 150.0, 149.9, 148.2 (dd, $J_{C,F}$ = 230.1, 15.7 Hz), 146.3 (dd, $J_{C,F}$ = 225.8, 14.7 Hz), 131.9 (d, $J_{C,F}$ = 9.9 Hz), 127.9 (d, $J_{C,F}$ = 3.7 Hz), 124.0, 122.9 (d, $J_{C,F}$ = 8.2 Hz), 112.4 (d, $J_{C,F}$ = 4.5 Hz), 105.8 (d, $J_{C,F}$ = 18.5 Hz), 97.6 (d, $J_{C,F}$ = 21.9 Hz), 59.0, 45.9, 45.2, 31.0; ^{19}F NMR (282 MHz, $CDCl_3$) δ –143.3 (d, $J_{F,F}$ = 19.7 Hz), –147.7 (d, $J_{F,F}$ = 22.6 Hz); HRMS (ESI$^+$) *m/z* calcd for $C_{18}H_{20}N_3F$ ([M+H]$^+$) 316.1620, found 316.1610.

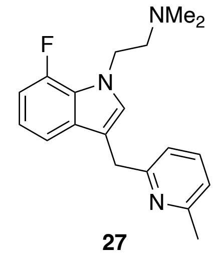

27

3j. 2-(7-Fluoro-3-((6-methylpyridin-2-yl)methyl)-1*H*-indol-1-yl)-*N*,*N*-dimethylethan-1-amine (**27**). A mixture of **17** (0.100 g, 0.416 mmol), 2-dimethylaminoethyl chloride hydrochloride (0.0666 g, 0.458 mmol), KI (0.0760 g, 0.458 mmol), and KOH (0.117 g, 2.08 mmol) in DMSO (1.04 mL) was stirred at room temperature for 24 h. EtOAc (10 mL) was added, and the organic layer was washed with ice-cold brine (3 x 5 mL). The combined aqueous layers were extracted with EtOAc (10 mL), and the combined organic layers were dried ($MgSO_4$), filtered, and concentrated. The crude residue was purified by reverse-phase chromatography on C18-SiO_2 (12 g, 10% MeCN/H_2O to 100% MeCN). Fractions containing the desired product were combined, diluted with H_2O (10 mL), and extracted with CH_2Cl_2 (4 x 30 mL). The combined organic extracts were dried ($MgSO_4$), filtered, and concentrated to afford **27** (0.0387 g, 0.124 mmol, 30%) as a yellow-brown oil: IR (ATR) ν_{max} 2932, 2858, 2824, 2774, 1592, 1576, 1498, 1456, 1371, 1338, 1294, 1242, 1229, 1151, 1090, 1058, 1048, 907, 778, 725 cm^{-1}; ^{1}H NMR (400 MHz, $CDCl_3$) δ 7.41 (t, J = 7.6 Hz, 1 H), 7.25 (d, J = 8.8 Hz, 1 H), 6.97–6.82 (m with apparent singlet at 6.95, 5 H), 4.35 (t, J = 7.2 Hz, 2 H), 4.22 (s, 2 H), 2.72 (t, J = 7.2 Hz, 2 H), 2.57 (s, 3 H), 2.30 (s, 6 H); ^{13}C NMR (100 MHz, $CDCl_3$) δ 160.4, 157.8, 150.1 (d, $J_{C,F}$ = 241.5 Hz), 136.8, 132.4 (d, $J_{C,F}$ = 5.6 Hz), 128.5, 124.4 (d, $J_{C,F}$ = 9.5 Hz), 120.8, 119.6, 119.3 (d, $J_{C,F}$ = 6.6 Hz), 115.4 (d, $J_{C,F}$ = 3.3 Hz), 113.6, 107.4 (d, $J_{C,F}$ = 18.1 Hz), 60.4 (d, $J_{C,F}$ = 1.2 Hz), 47.0 (d, $J_{C,F}$ = 4.6 Hz), 45.8, 34.5, 24.7; ^{19}F NMR (282 MHz, $CDCl_3$) δ –136.2; HRMS (ESI$^+$) m/z calcd for $C_{19}H_{23}N_3F$ ([M+H]$^+$) 312.1871, found 312.1859.

Assays

Light-induced retinal degeneration

Animals. BALB/c albino mice aged 5-8 weeks were used. Mice were housed in a temperature-controlled animal facility with a 12-hour light/dark cycle (lights on 7 am, lights off 7 pm). Food and water were provided *ad libitum*. All procedures were conducted in accordance with the Directive 86/609/EEC for animal experiments, FELASA Guidelines and Recommendations, and ARVO Statement for the Use of Animals in Ophthalmic and Vision Research. Experiments were approved by the Finnish Project Authorization Board, with protocol number ESAVI/26320/2021.

Study items. (+)- and (-)-Lysergols, isolysergols, clavines, and Compounds **18**, **19**, **20** and **28**, or bromocriptine (a known partial agonists for several 5-HT receptors and dopamine-2/3-receptors), presented in solid form, were dissolved in DMSO to obtain 10 mg/mL stock solutions. The stock solutions were stored at -20 °C until use. On the day of the experiment, the stock and vehicle (25% dimethyl sulfoxide, 75% saline) were used for the drug preparation. All experiments were conducted at a set dose of 10 mg/kg per body weight, and the total volume of injection was set at 100 µL / animal.

Drug administration and LIRD induction. The design of the experiment is presented in Supplementary Figure 1. Mice were randomized, marked, and placed in the darkroom for overnight dark adaptation one day before the experiments. On the first day of the experiment, dark-adapted mice were intraperitoneally (i.p.) injected with either a drug or vehicle solution under dim red-light observation. Mydriasis was achieved through the topical application of metaoxedrin-tropicamide drops (Oftan®TROPICAMID, 5 mg/mL, Oftan®METAOKSEDRIN 100 mg/ml; Santen Oy, Tampere, Finland; mixed in a 1:5 ratio) 30 and 15 minutes before light exposure. Starting 30 minutes after drug/vehicle administration, freely moving mice were exposed to bright light at 15 kLux for 30 minutes and transferred back to the vivarium thereafter. A maximum of 6 mice were bright light-exposed in the same trial, and each trial consisted of at least one vehicle-treated mouse. The manipulations were performed without separation of sexes; however, when feasible, female and male mice underwent the LIRD procedure in different sessions.

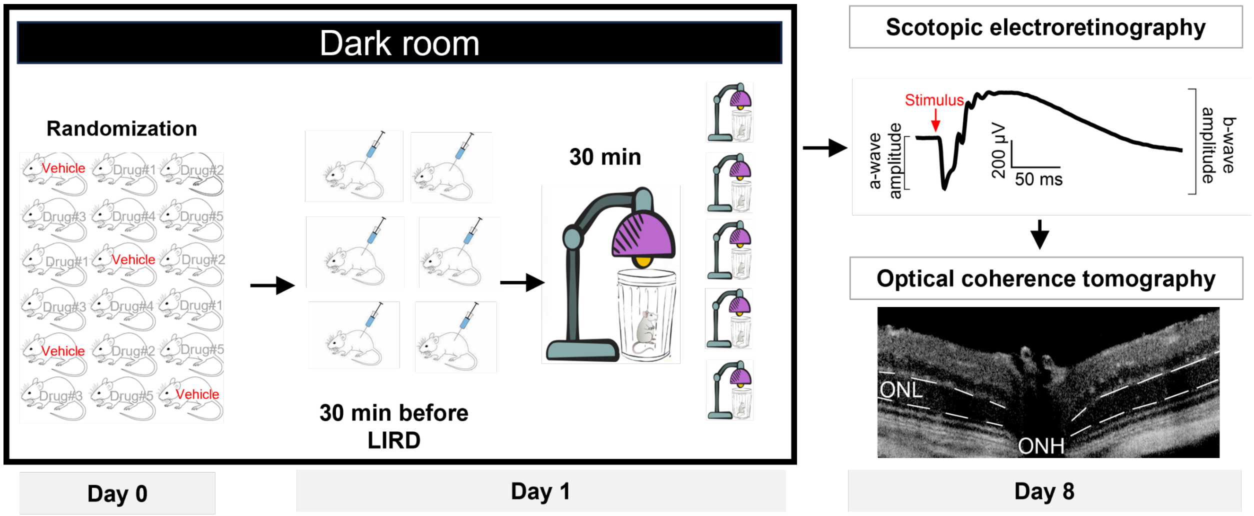

Supplementary Figure 1. LIRD study design.

Electroretinography (ERG). The ERG was performed in fully dark-adapted mice anesthetized by intraperitoneal injection of 60 mg/kg ketamine (Ketaminol®Vet, MSD Animal Health, Espoo, Finland) and 0.4 mg/kg medetomidine mixture (Domitor®Vet, Orion pharma, Espoo, Finland). Pupils were dilated similarly as before LIRD induction. The eye drops were instilled twice: before the injection of anesthetics, and immediately when the animal was unconscious. One drop of carbomer (2 mg/g) eye lubricant (Viscotears, Bausch & Lomb Nordic AB, Stockholm, Sweden) was then applied to prevent the corneas from drying, to protect from abrasion during the procedures, and to increase conductivity during ERG recording. The animals were kept warm throughout the procedure using a controlled heating pad (TMP-5b, Supertech Ltd., Pecs, Hungary) maintained at 37°C. Both eyes of the mice were then stimulated simultaneously using an Espion full-field ERG device (Espion E3 console, Diagnosys LLC, Lowell, MA). The active electrodes were silver-wire loops that were placed on the corneas, and reference and electrode were subdermal needles inserted into the head between the eyes and into lower back, respectively. The in-house scripted (Diagnosys Espion V6 software) scotopic ERG protocol utilized monochromatic green light stimulation and included the following steps:

- Step 1: Intensity 0.00001 cd·s/m^2; inter-stimulus interval (ISI) 1 s, 25 sweeps
- Step 2: 0.0001 cd·s/m^2; ISI 2 s, 20 sweeps
- Step 3: 0.0001 cd·s/m^2; ISI 2 s, 20 sweeps
- Step 4: 0.0005 cd·s/m^2; ISI 3 s, 15 sweeps
- Step 5: 0.001 cd·s/m^2; ISI 5 s, 12 sweeps
- Step 6: 0.01 cd·s/m^2; ISI 10 s, 8 sweeps
- Step 7: 0.05 cd·s/m^2; ISI 10 s, 8 sweeps
- Step 8: 0.1 cd·s/m^2; ISI 15 s, 4 sweeps

- Step 9: 0.5 cd·s/m^2; ISI 20 s, 4 sweeps
- Step 10: 1 cd·s/m^2; ISI 20 s, 3 sweeps
- Step 11: 10 cd·s/m^2; ISI 45 s, 2 sweeps
- Step 12: 30 cd·s/m^2; ISI 60 s, 2 sweeps

The ERG signal was acquired at 2 kHz and filtered with a low-frequency cutoff at 1 Hz and a high-frequency cutoff at 300 Hz. Espion software automatically detected the ERG a-wave (first negative ERG component) and the b-wave (first positive ERG component); a-wave amplitude was measured from the signal baseline, whereas b-wave amplitude was measured as the difference between the negative trough (a-wave) and the highest positive peak. The correct detection of a- and b-wave through and peak, respectively, was visually confirmed during offline analysis.

Optical coherence tomography. OCT imaging was performed using the Phoenix Micron IV ophthalmic imaging system (Phoenix-Micron Inc., Bend, OR). Field of view was centered to the optic nerve head (OHN) and images at vertical and horizontal orientations were acquired. ImageJ 1.47v software (National Institutes of Health, Bethesda, MD) and a digital ruler tool was used to measure outer nuclear layer (ONL) thickness at temporal, nasal, superior, and inferior retinal sites at 500 μm distance from ONH border.

Statistical analysis. Data normality was tested by using the Shapiro-Wilk test. In the main figures, ONL thickness analysis was performed using the Kruskal-Wallis (K-W) test followed by Dunn´s multiple comparisons tests, whereas the ERG data was analyzed by Welch´s ANOVA followed by Dunnett´s T3 tests. All analyses were performed using the Prism 10 software (GraphPad Software LLC, Boston, MA). All data is presented as means ± SEM and the level of statistical significance was set at $P < 0.05$.

Results. Supplementary Figures 2-4 display a detailed view of ONL and ERG data, whereas main text figures show averaged ONL thickness data over retinal quadrants and ERG amplitude data near the saturation level, at 10 cd·s/m^2 stimulus.

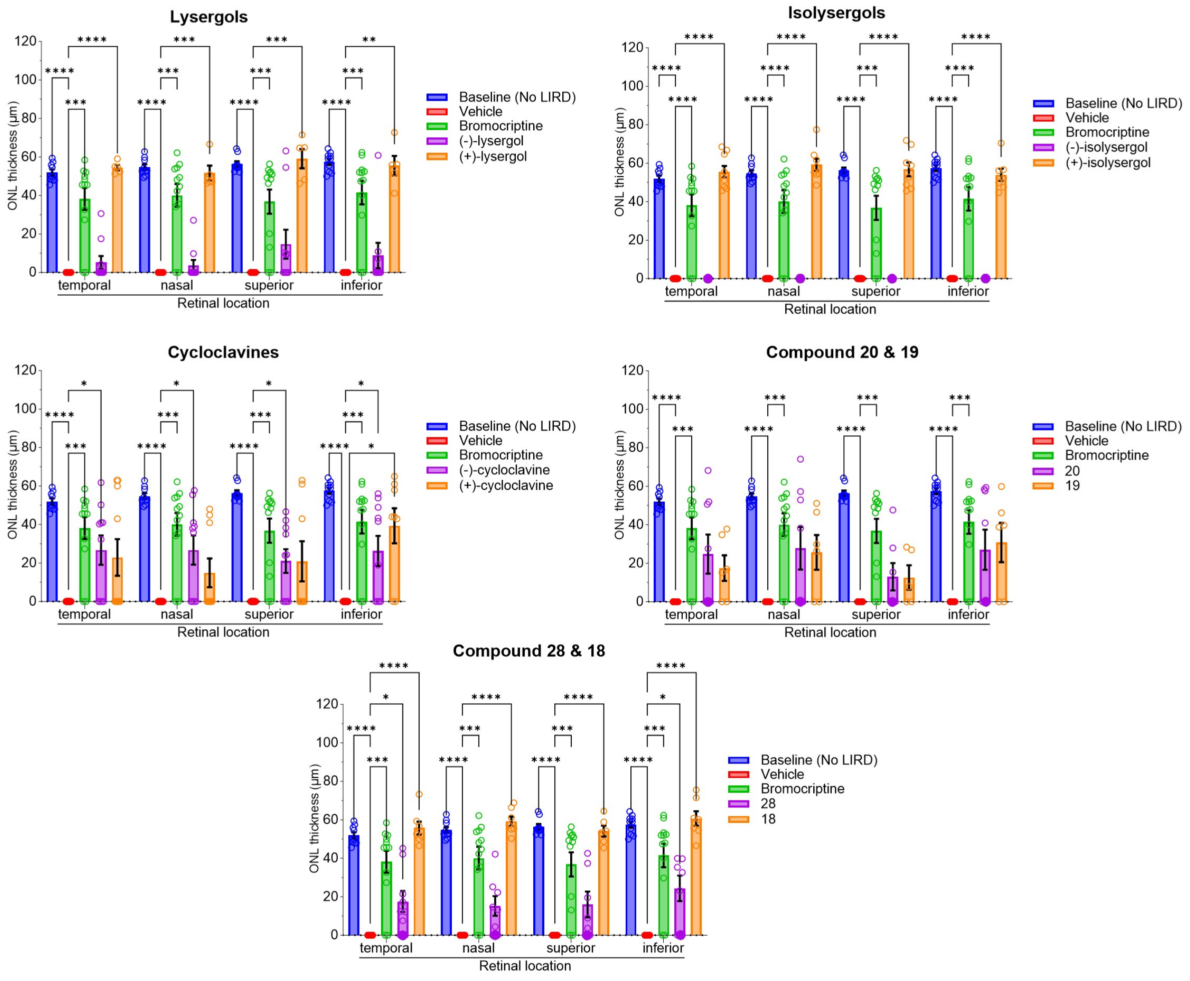

Supplementary Figure 2. Detailed outer nuclear layer (ONL) thickness analysis. For Manuscript Figure 5, ONL thickness at quadrants was averaged for each mouse.

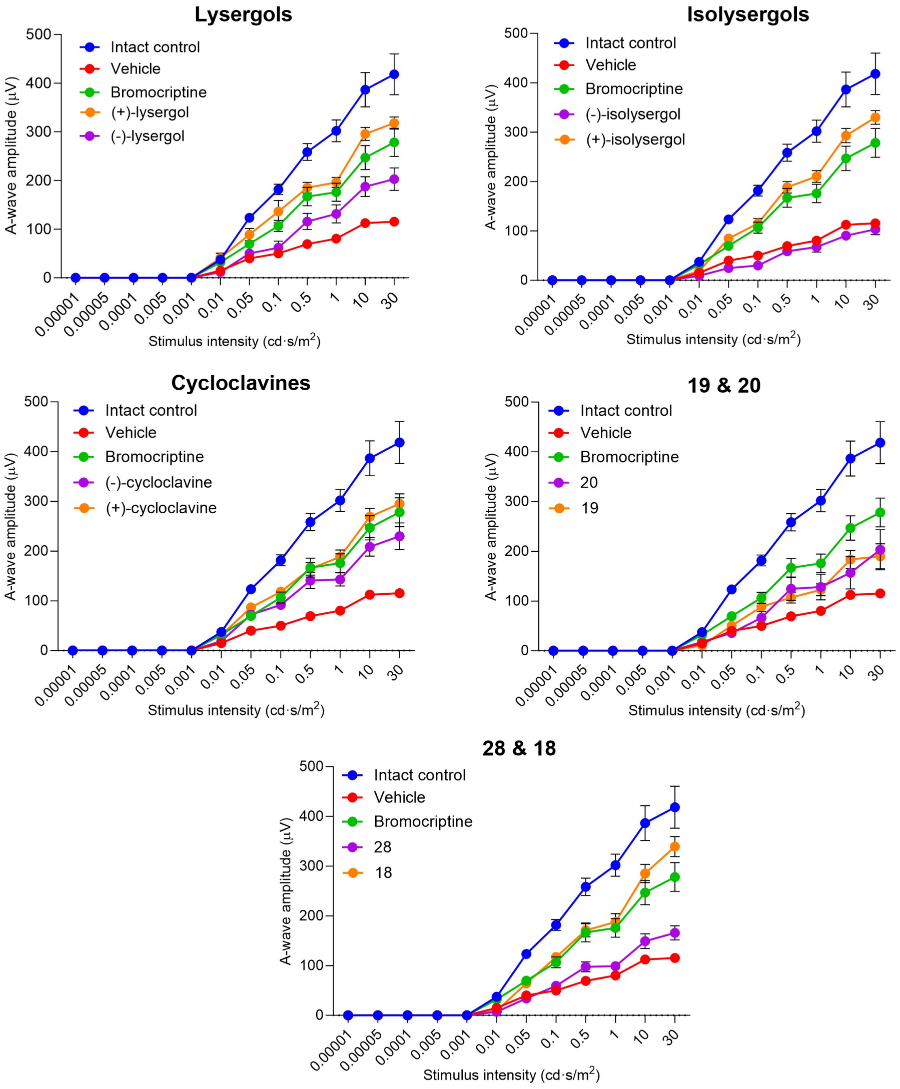

Supplementary Figure 3. Detailed ERG a-wave amplitude analysis. Data from one stimulation intensity (10 cd·s/m^2) was shown in Manuscript Figure 6.

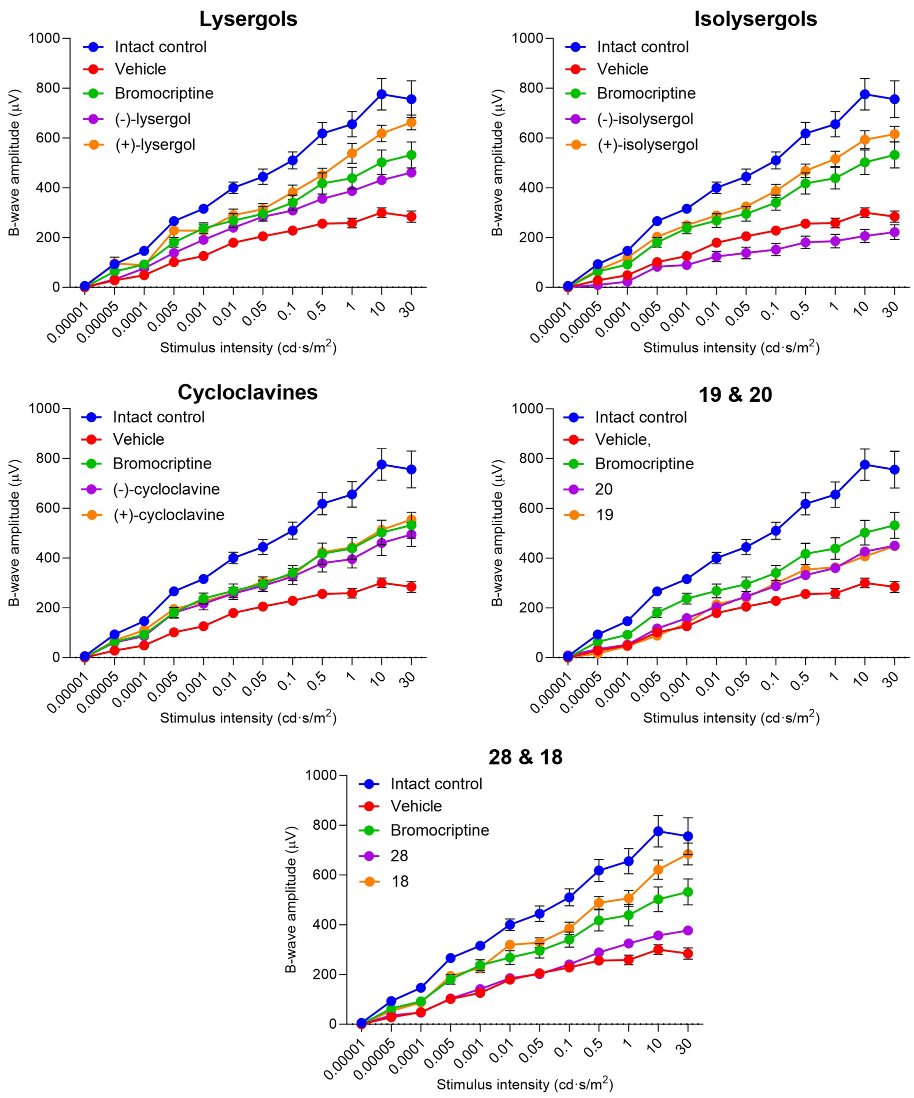

Supplementary Figure 4. Detailed ERG b-wave amplitude analysis. Data from one stimulation intensity (10 cd·s/m^2) was shown in Manuscript Figure 6.

Copies of NMR Spectra

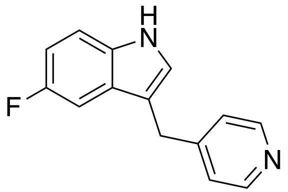

13
500 MHz, $CDCl_3$

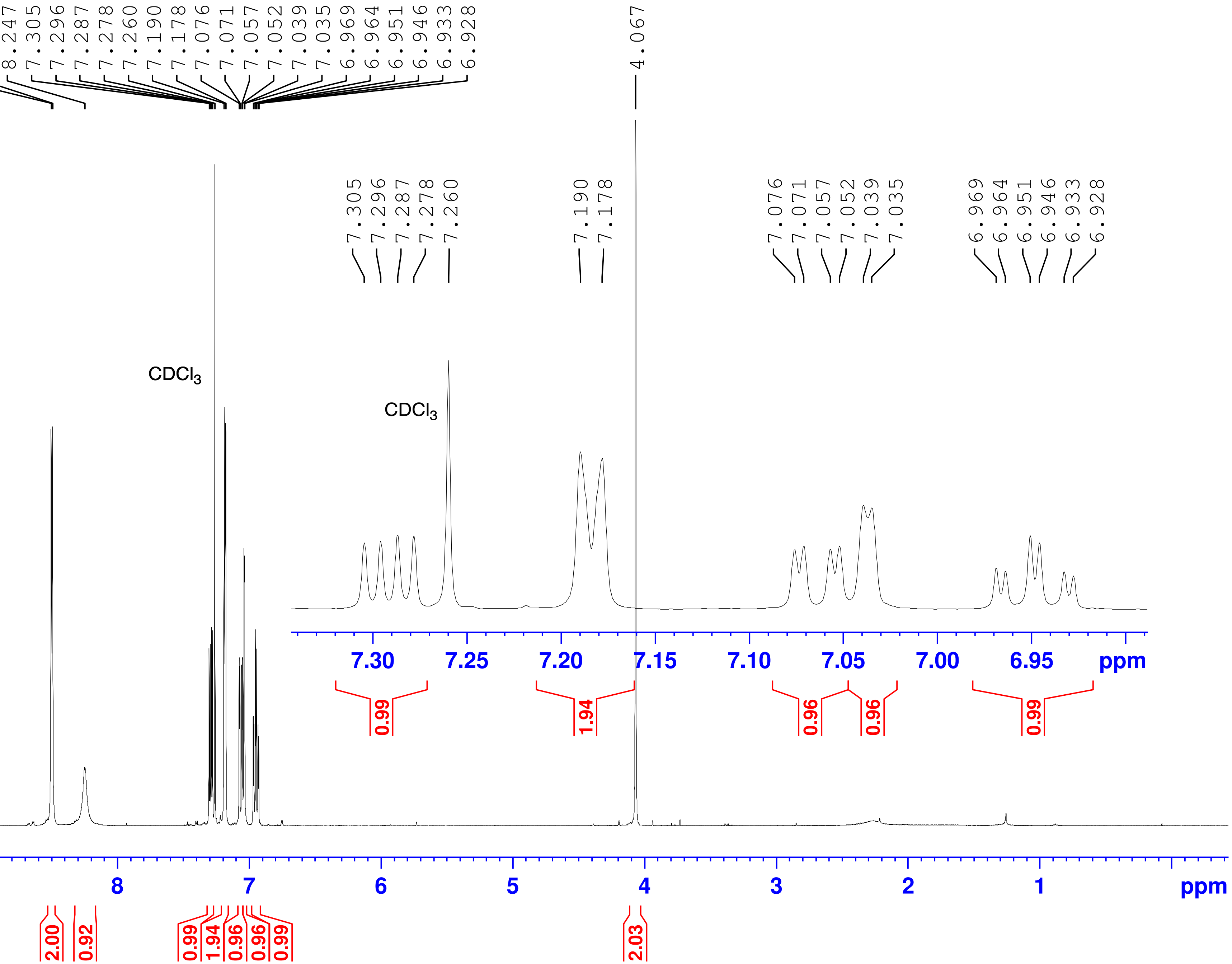

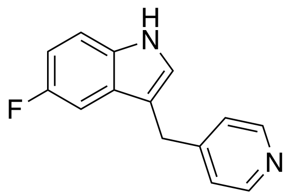

13
125 MHz, $CDCl_3$

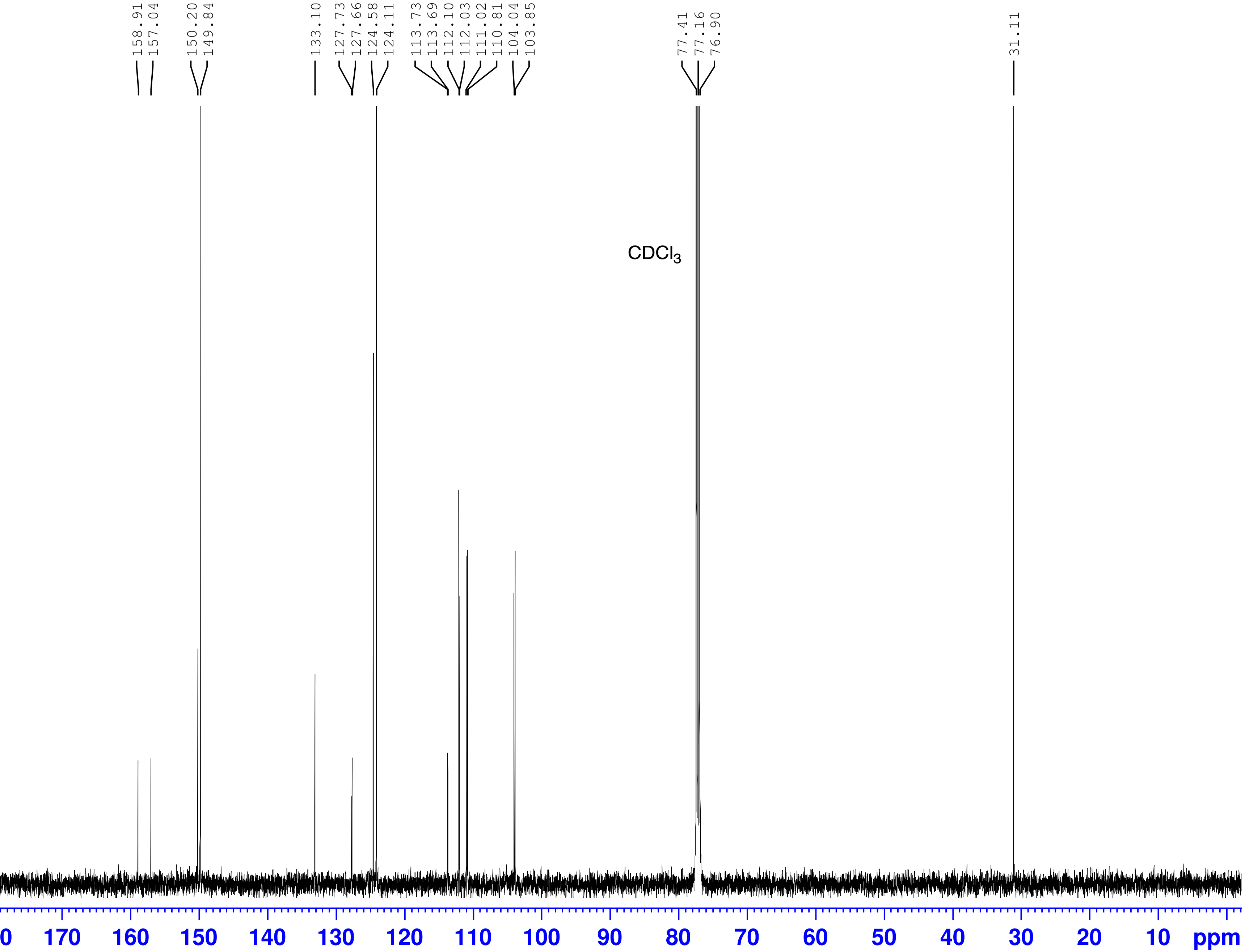

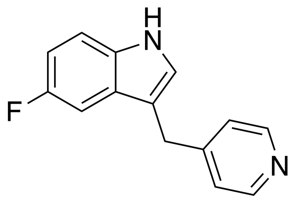

13
282 MHz, $CDCl_3$

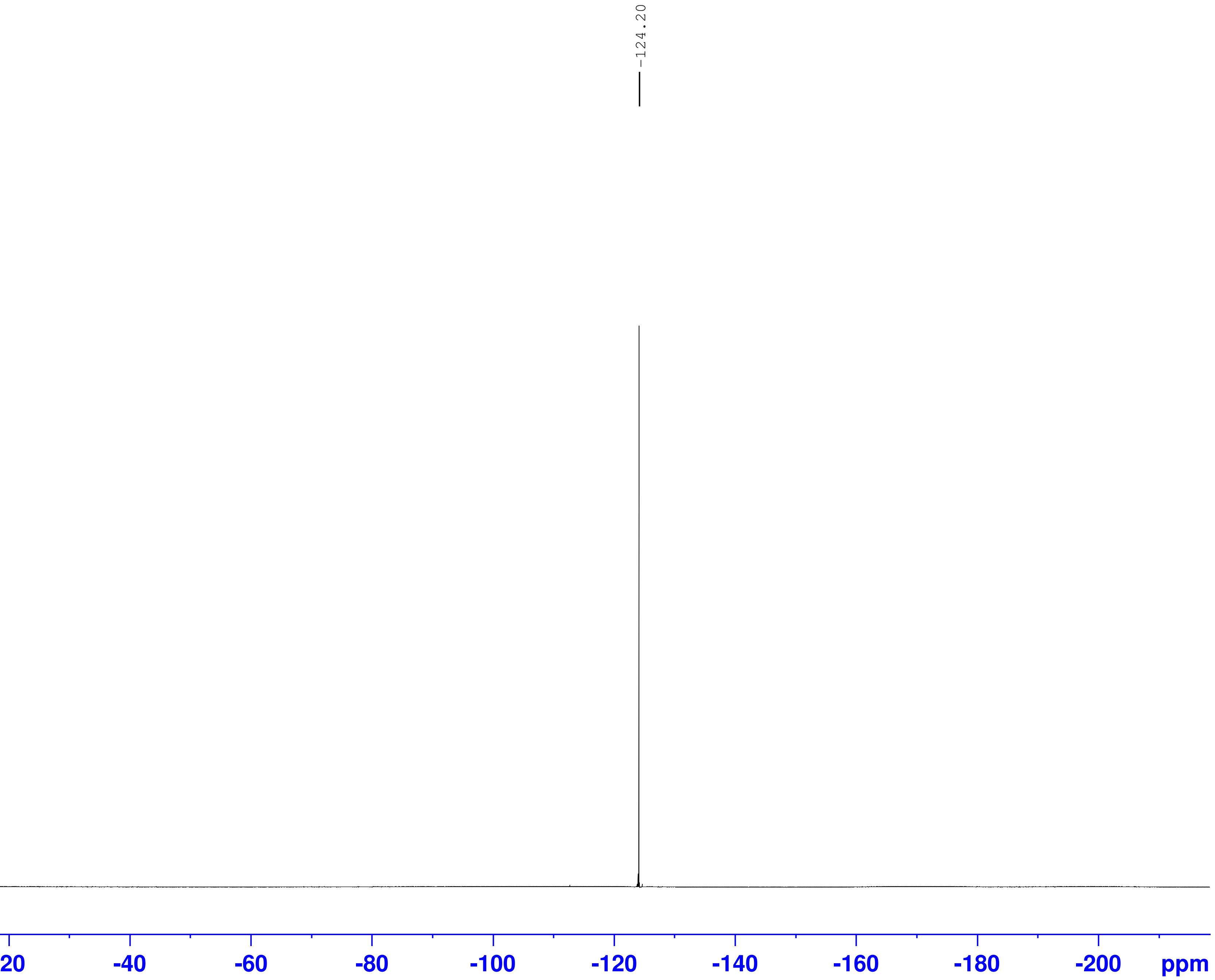

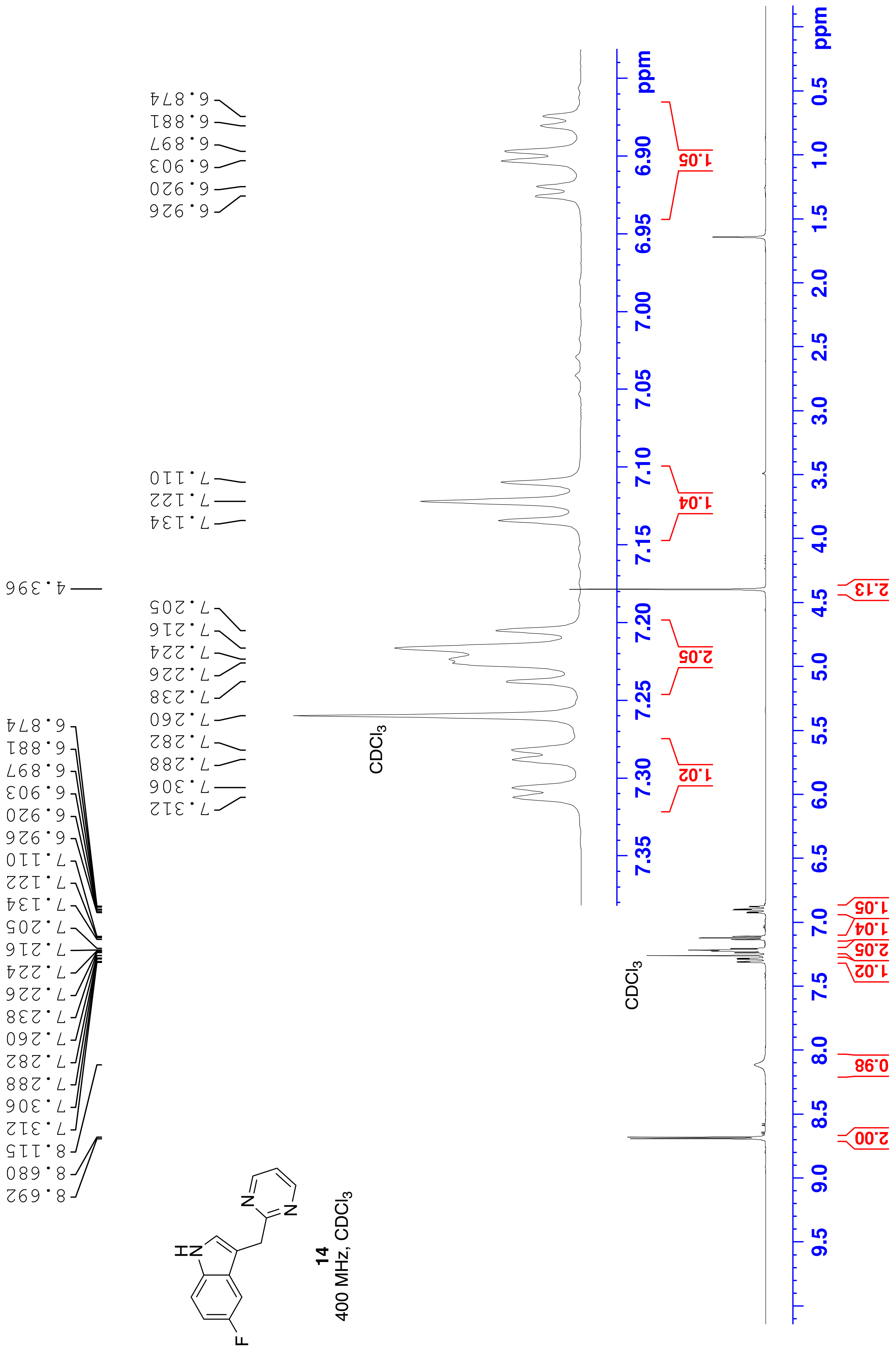
14
400 MHz, CDCl3
CDCl3
ppm
8.692
8.680
8.115
7.312
7.306
7.288
7.282
7.260
7.238
7.226
7.224
7.216
7.205
7.134
7.122
7.110
6.926
6.920
6.903
6.897
6.881
6.874
4.396
2.00
0.98
1.02
2.05
1.04
1.05
2.13

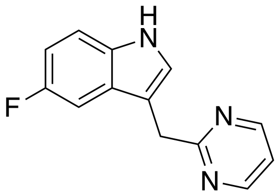

14
100 MHz, $CDCl_3$

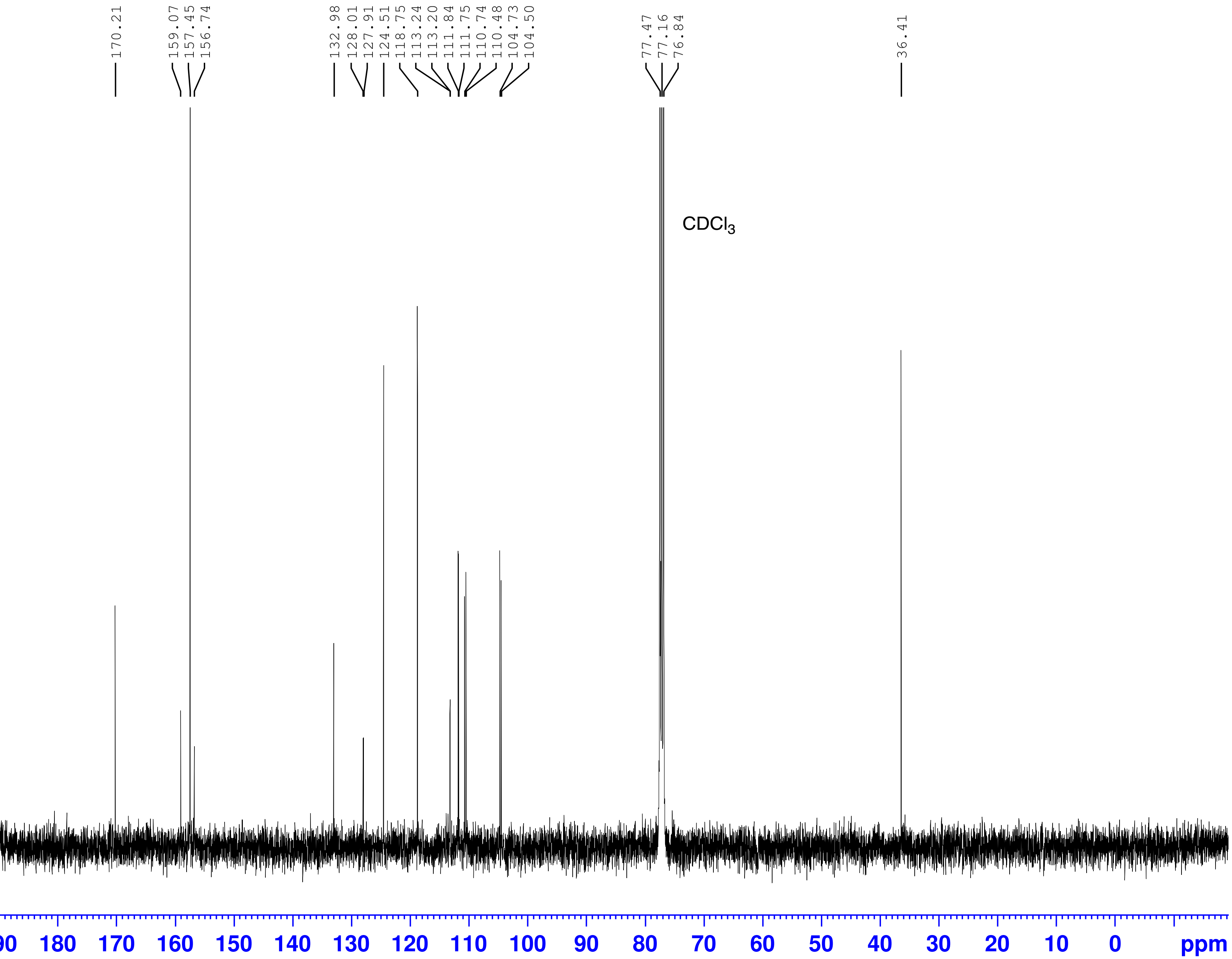

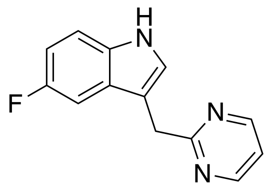

14
282 MHz, $CDCl_3$

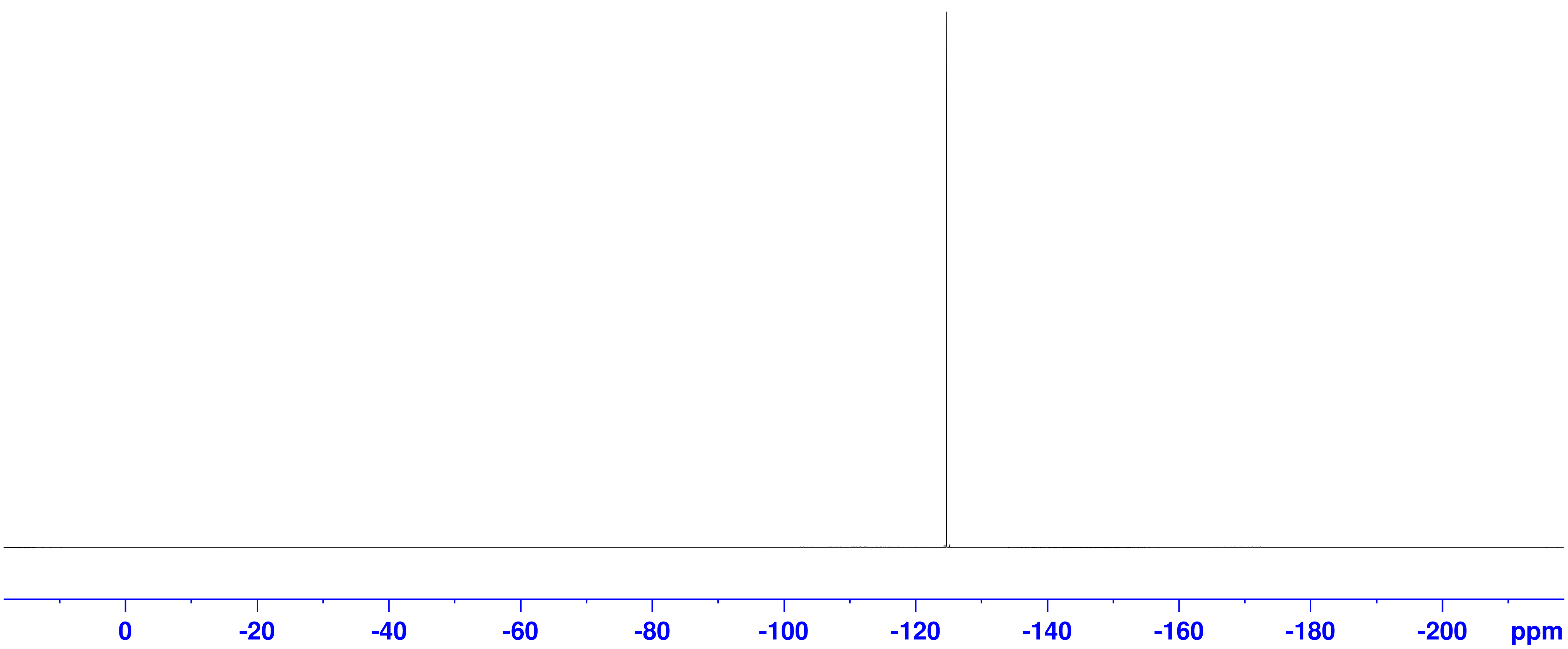

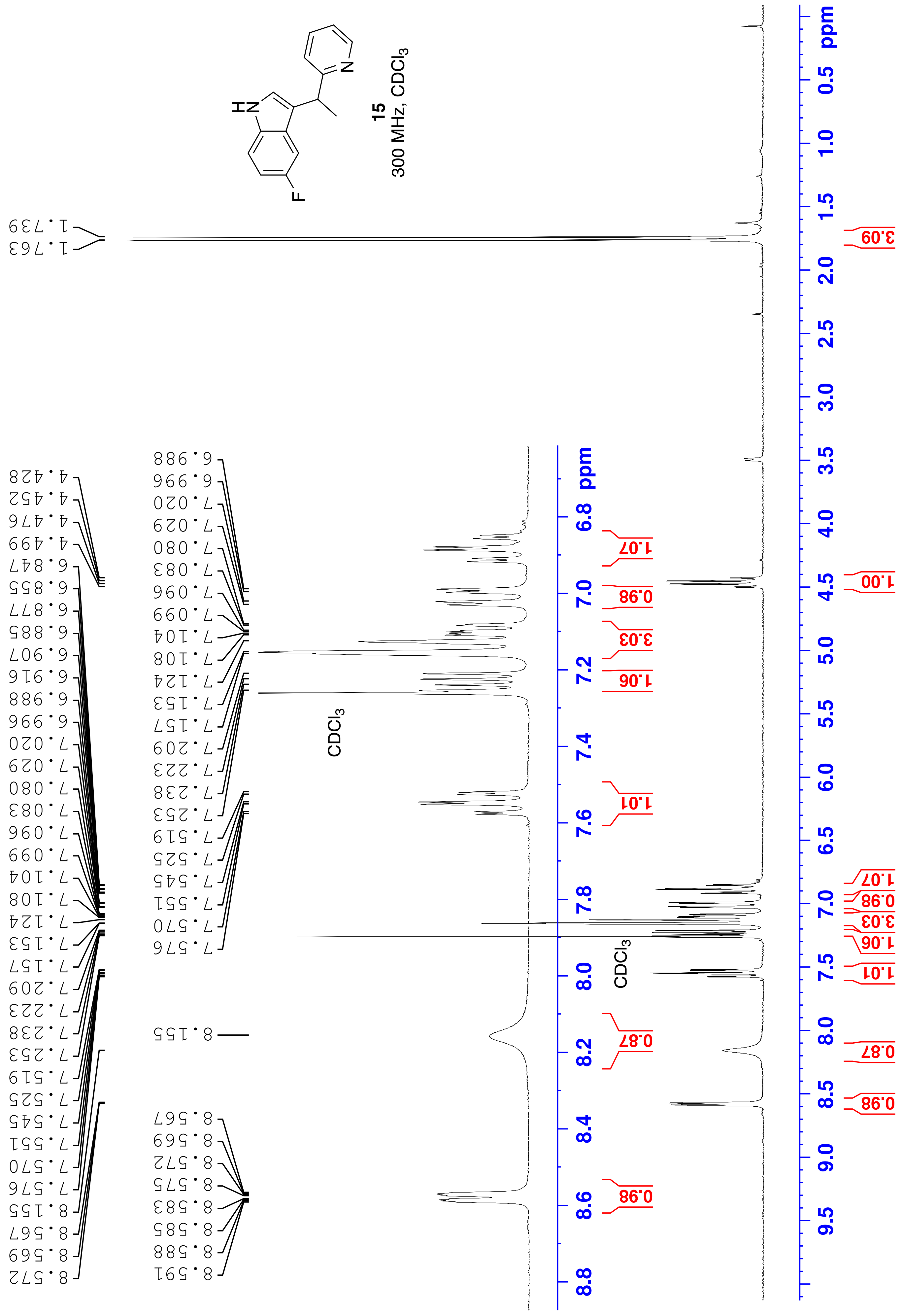
15
300 MHz, CDCl3
CDCl3
CDCl3
ppm
1.739
1.763
4.428
4.452
4.476
4.499
6.847
6.855
6.877
6.885
6.907
6.916
6.988
6.996
7.020
7.029
7.080
7.083
7.096
7.099
7.104
7.108
7.124
7.153
7.157
7.209
7.223
7.238
7.253
7.519
7.525
7.545
7.551
7.570
7.576
8.155
8.567
8.569
8.572
8.575
8.583
8.585
8.588
8.591
3.09
1.00
1.07
0.98
3.03
1.06
1.01
0.87
0.98

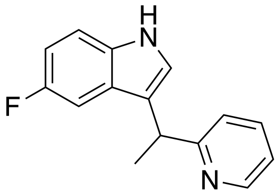

15
75 MHz, $CDCl_3$

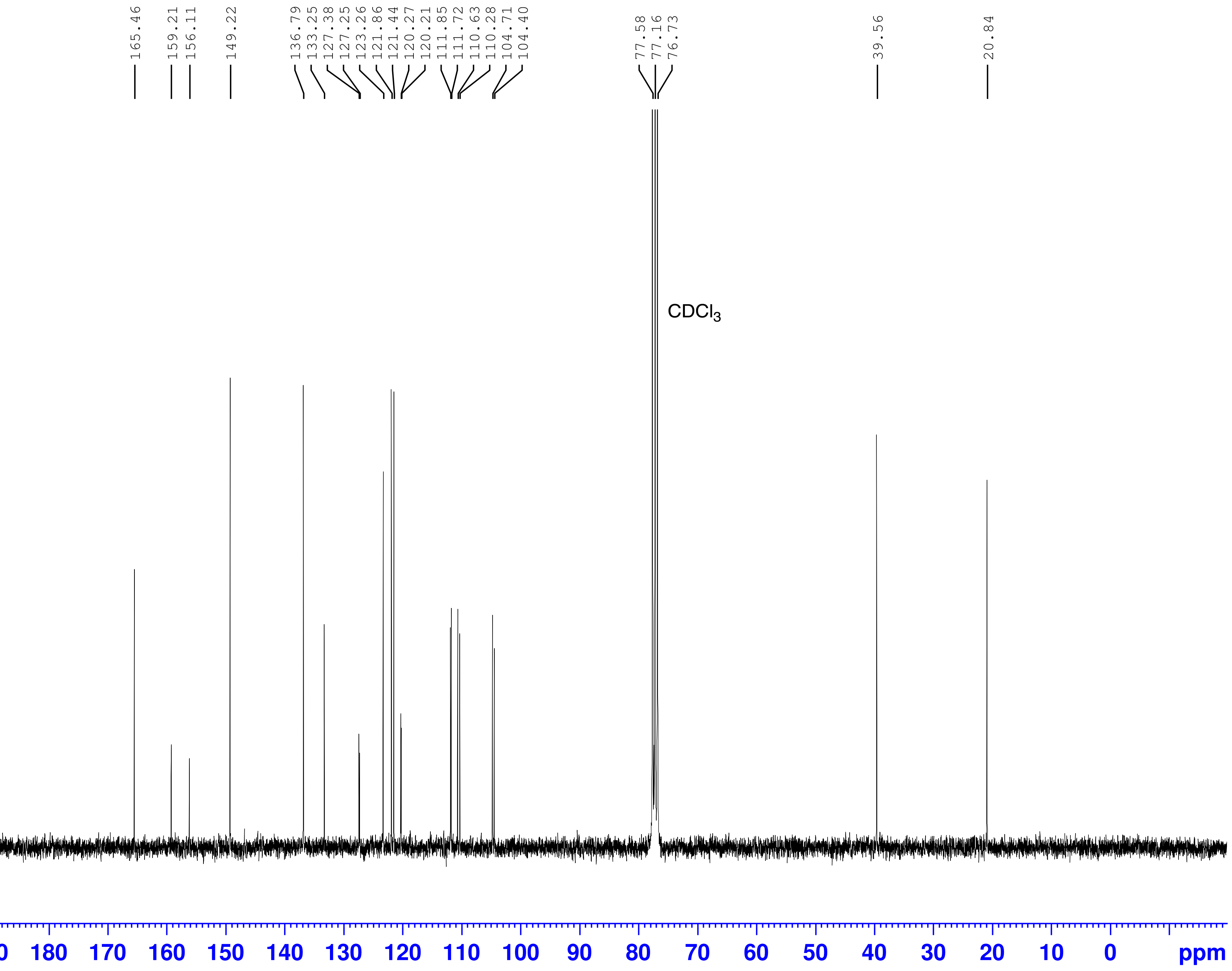

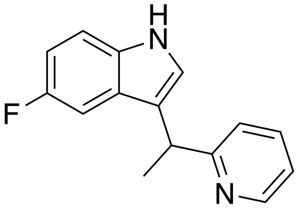

15
282 MHz, $CDCl_3$

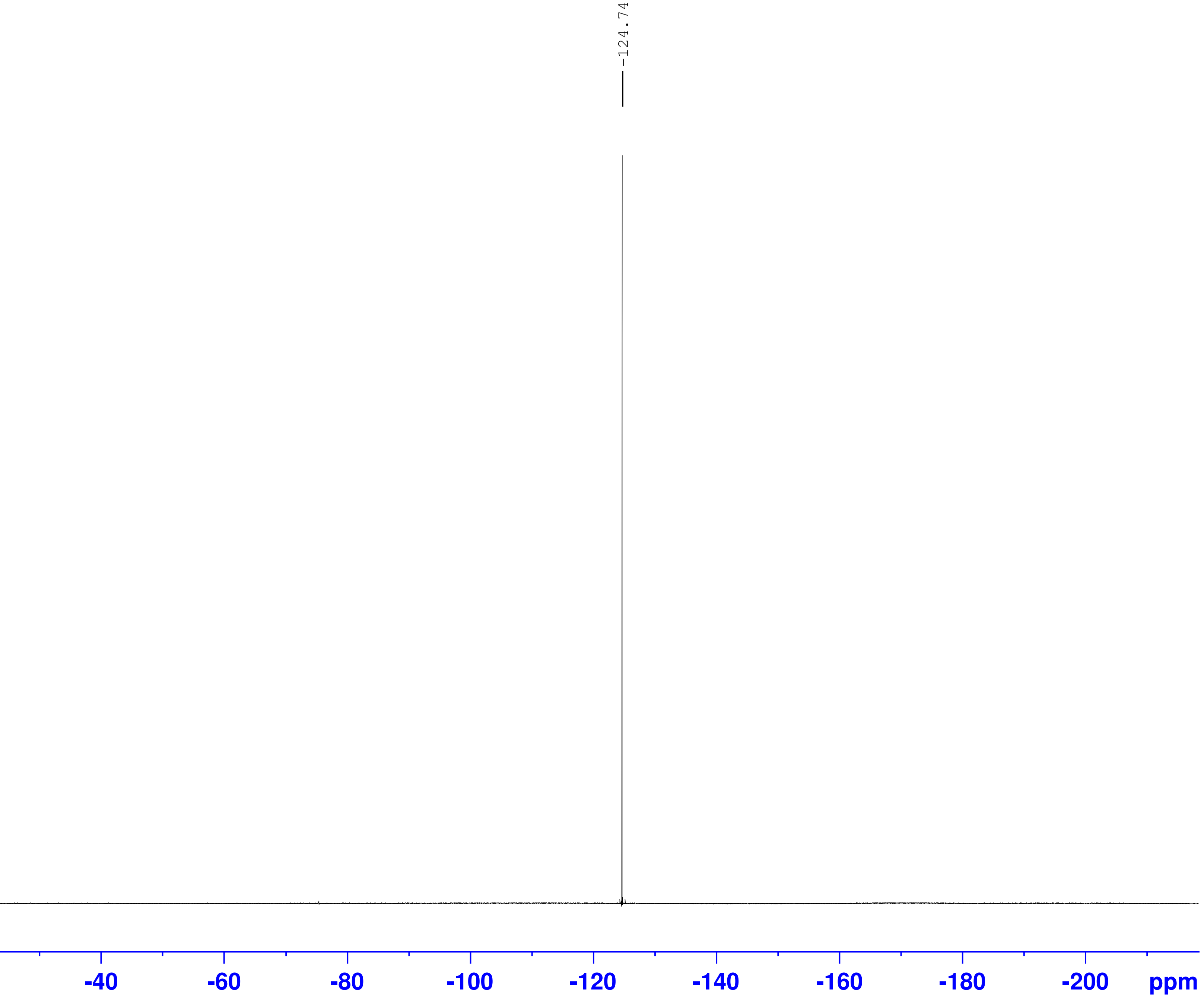

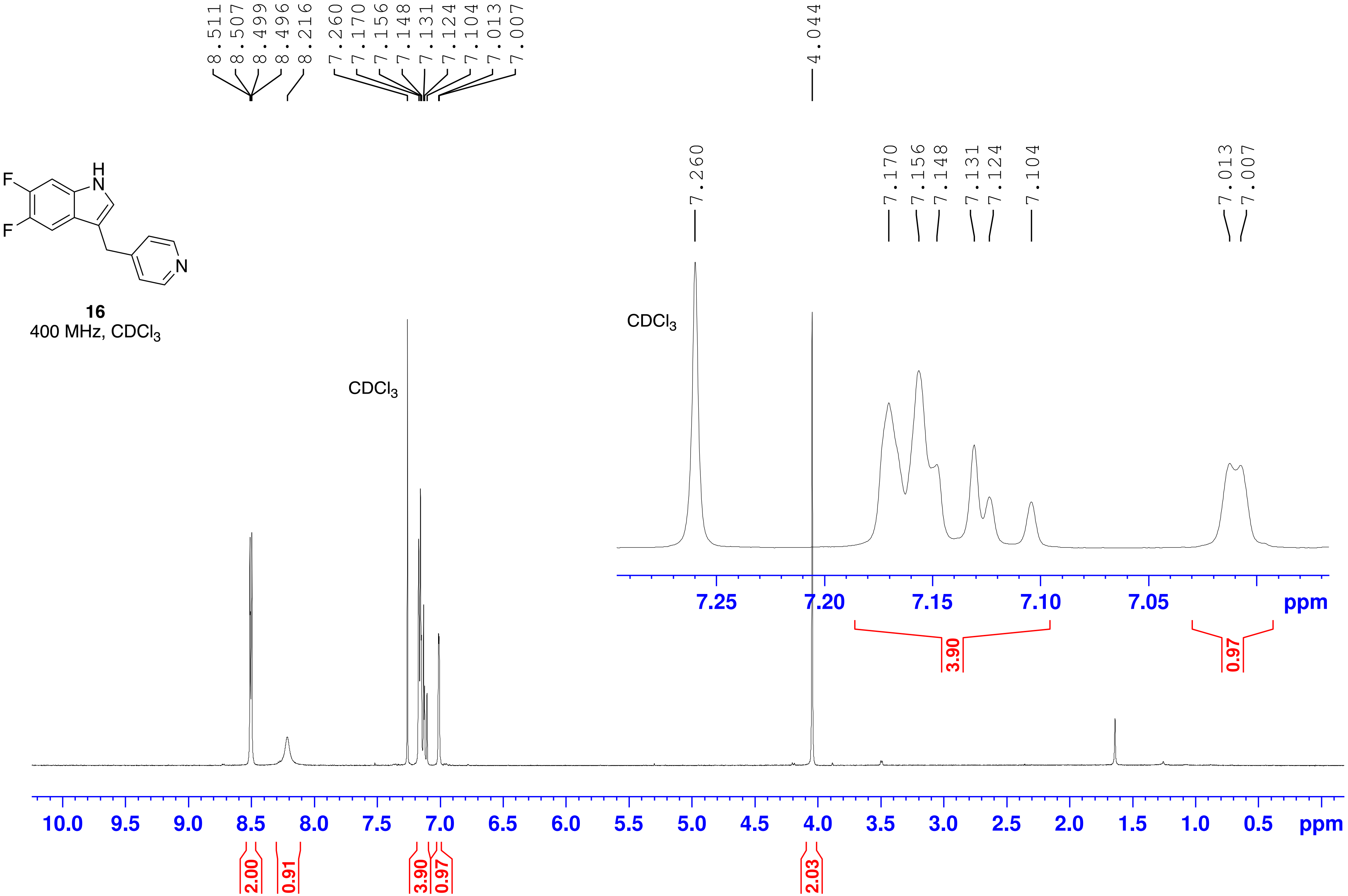

16
400 MHz, $CDCl_3$

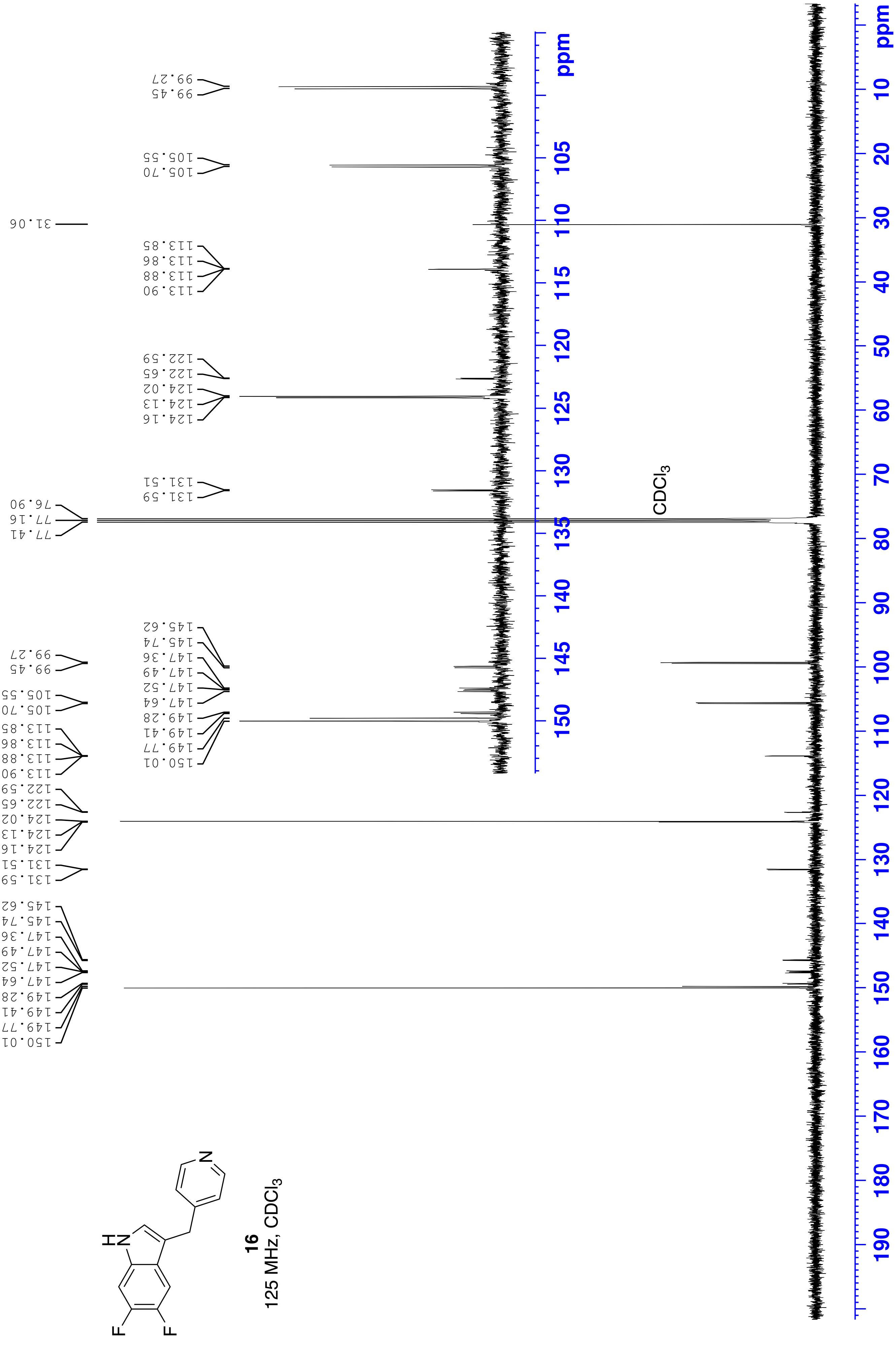

16
125 MHz, $CDCl_3$

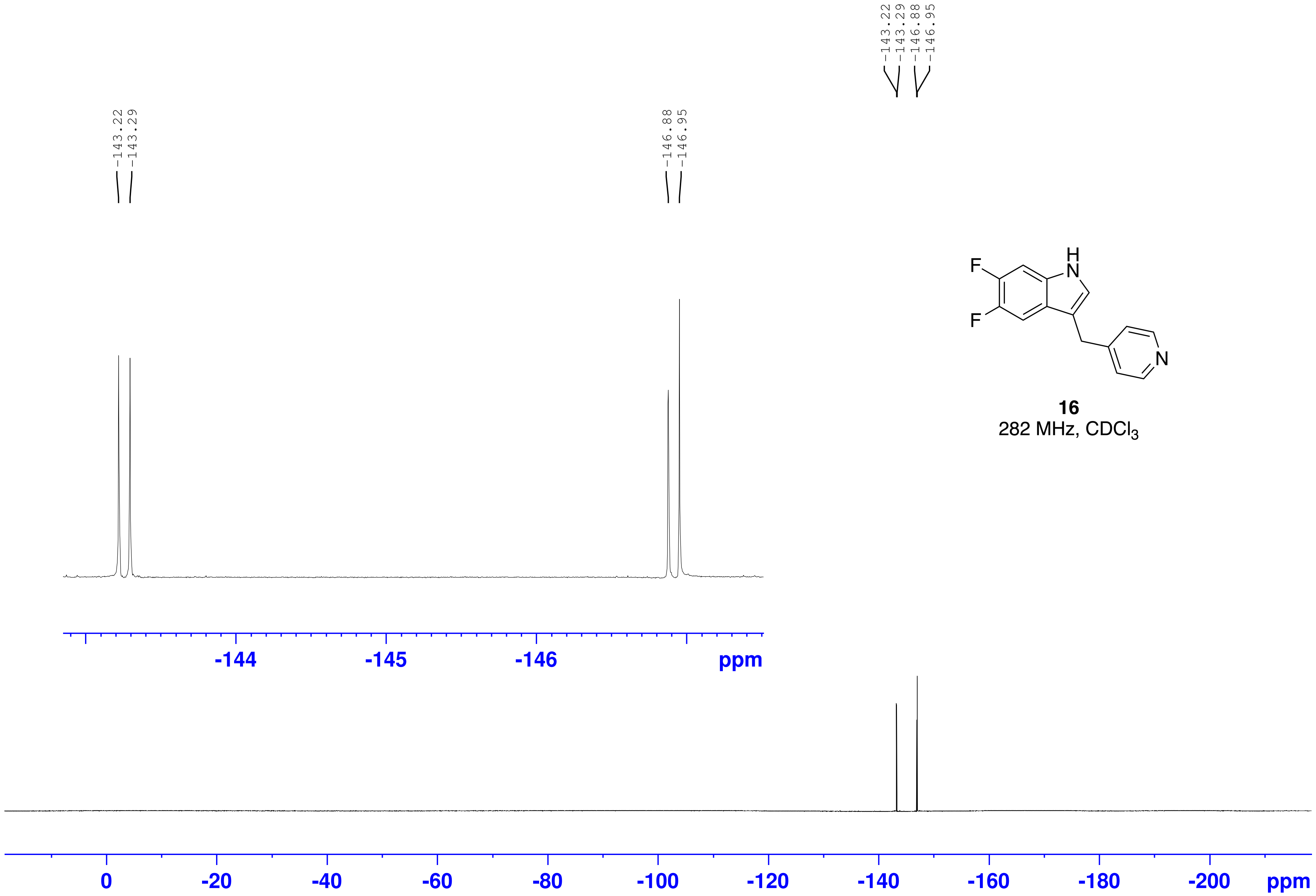

16
282 MHz, $CDCl_3$

17
500 MHz, $CDCl_3$

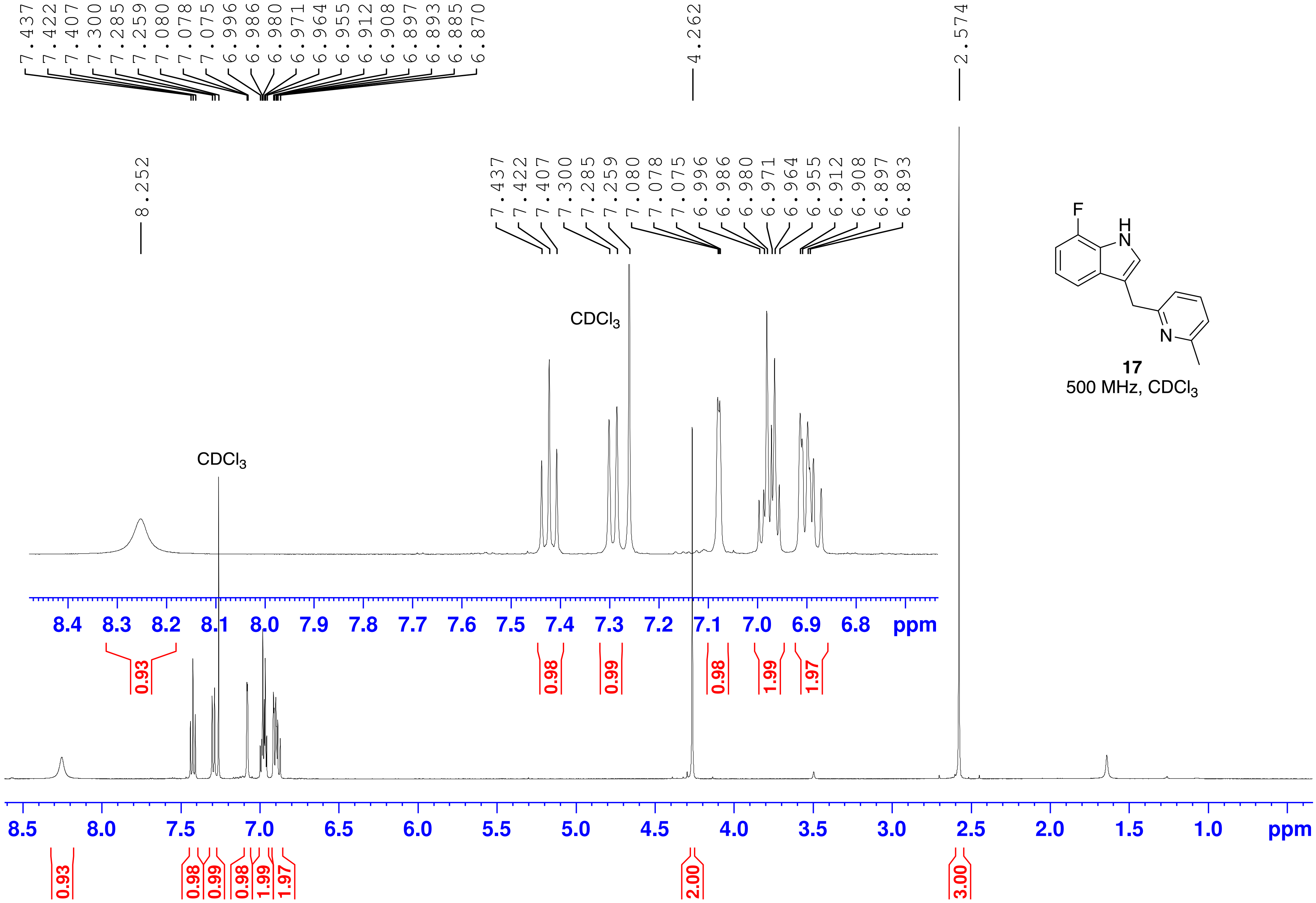

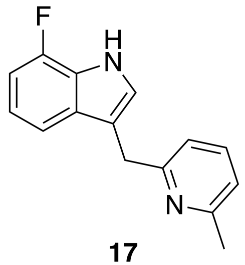

17
125 MHz, $CDCl_3$

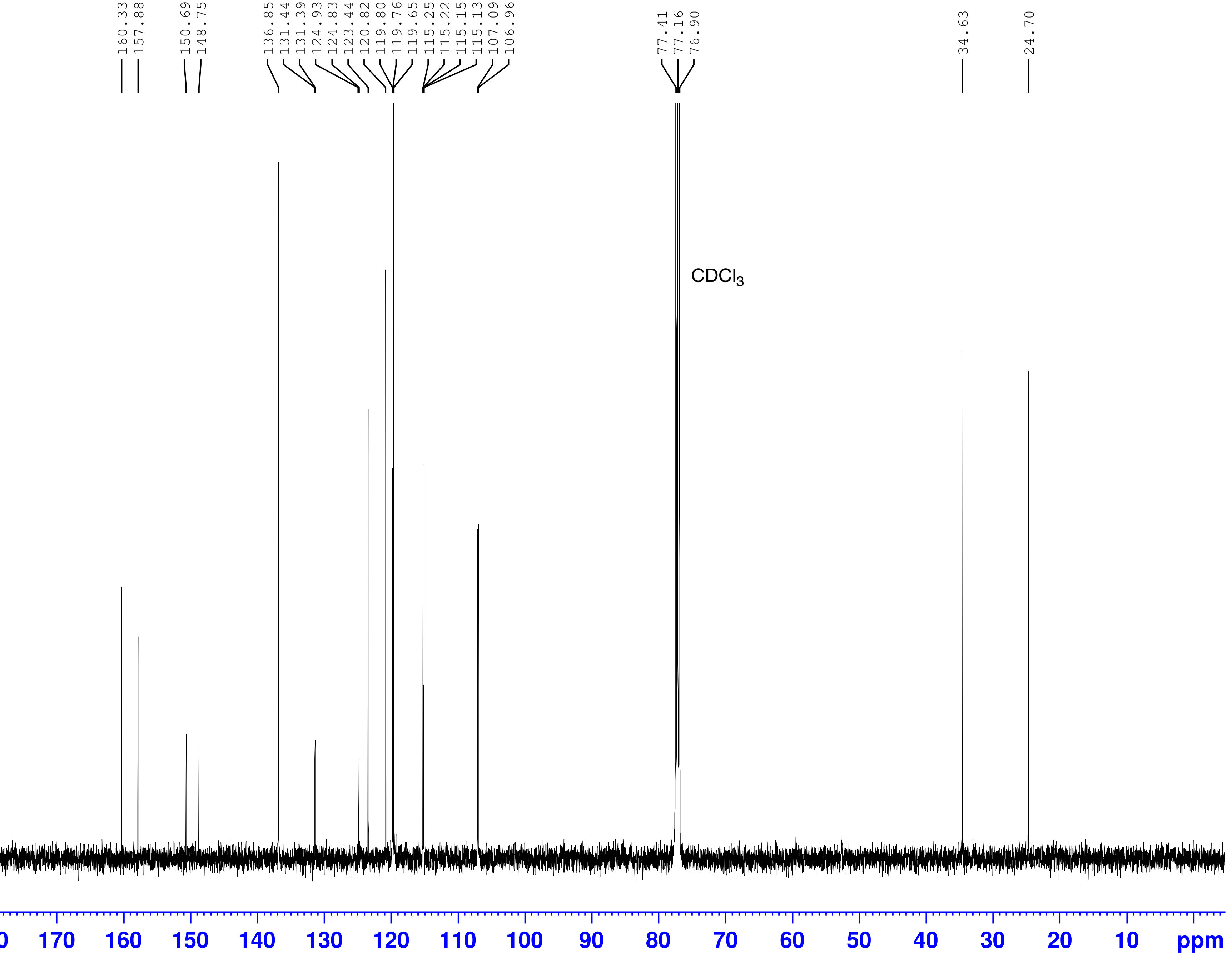

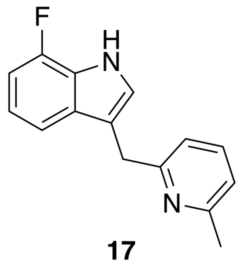

17
282 MHz, $CDCl_3$

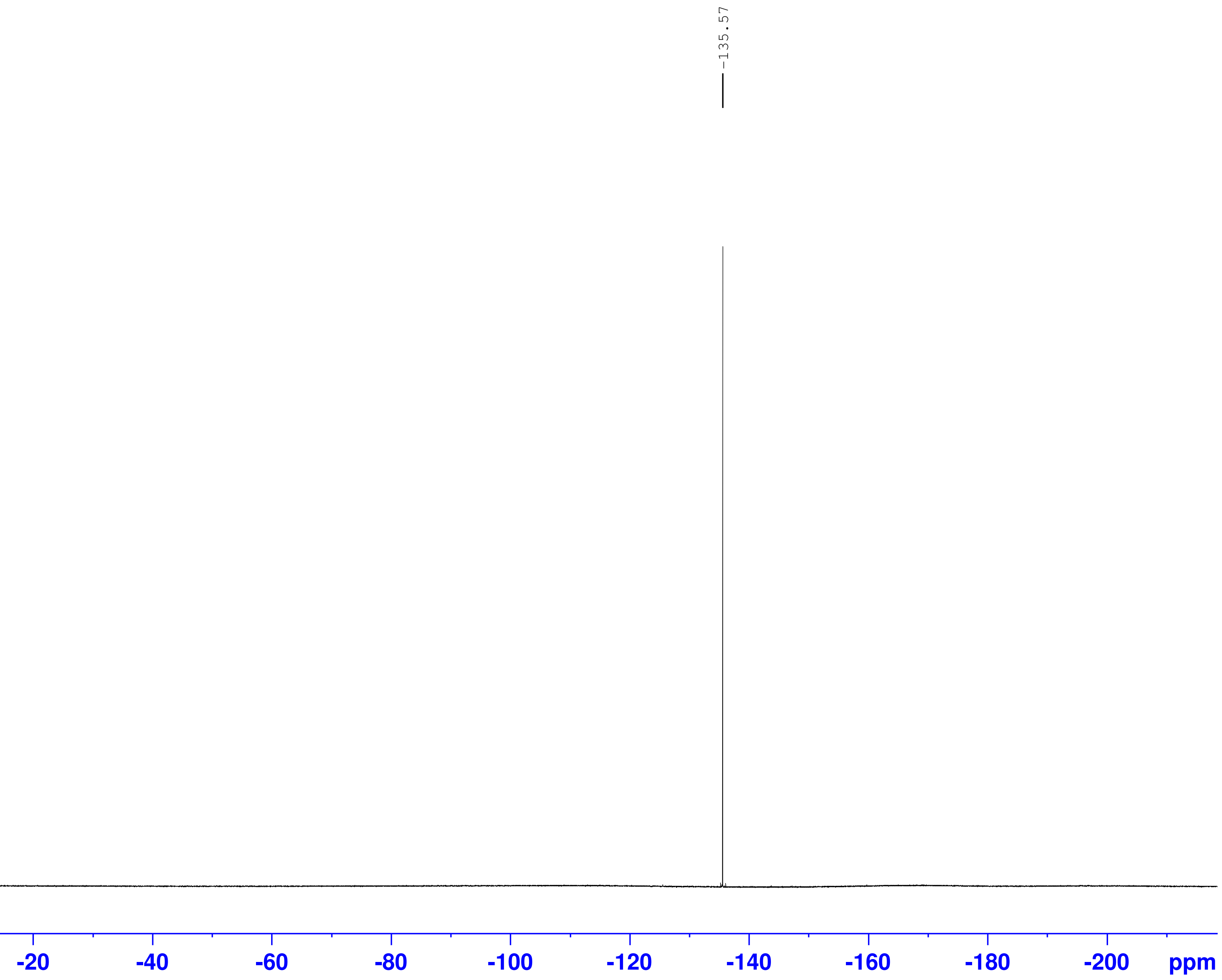

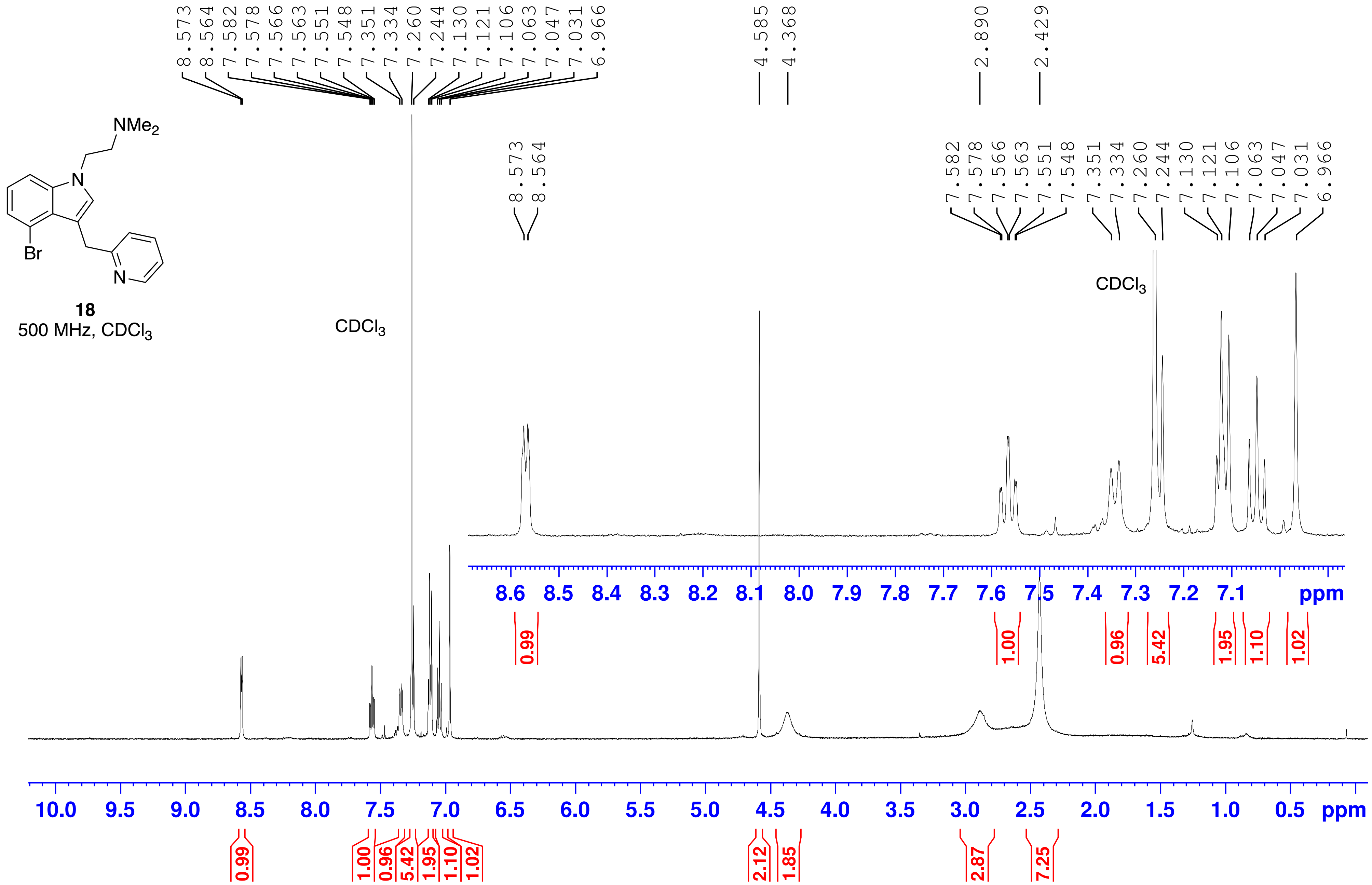

18
500 MHz, $CDCl_3$

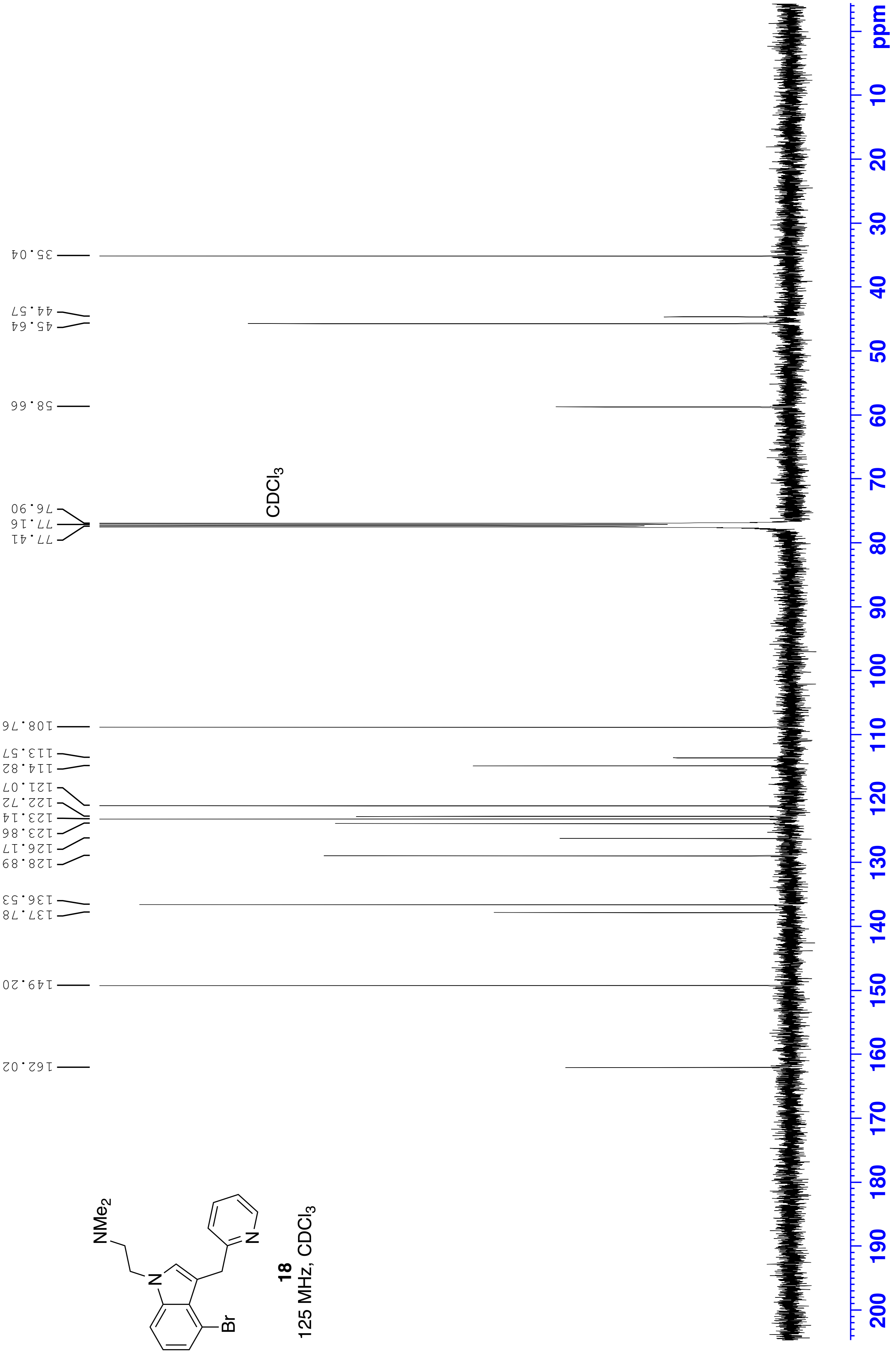
ppm
10
20
30
40
50
60
70
80
90
100
110
120
130
140
150
160
170
180
190
200
35.04
44.57
45.64
58.66
76.90
77.16
77.41
CDCl3
108.76
113.57
114.82
121.07
122.72
123.14
123.86
126.17
128.89
136.53
137.78
149.20
162.02
NMe2
N
Br
N
18
125 MHz, CDCl3

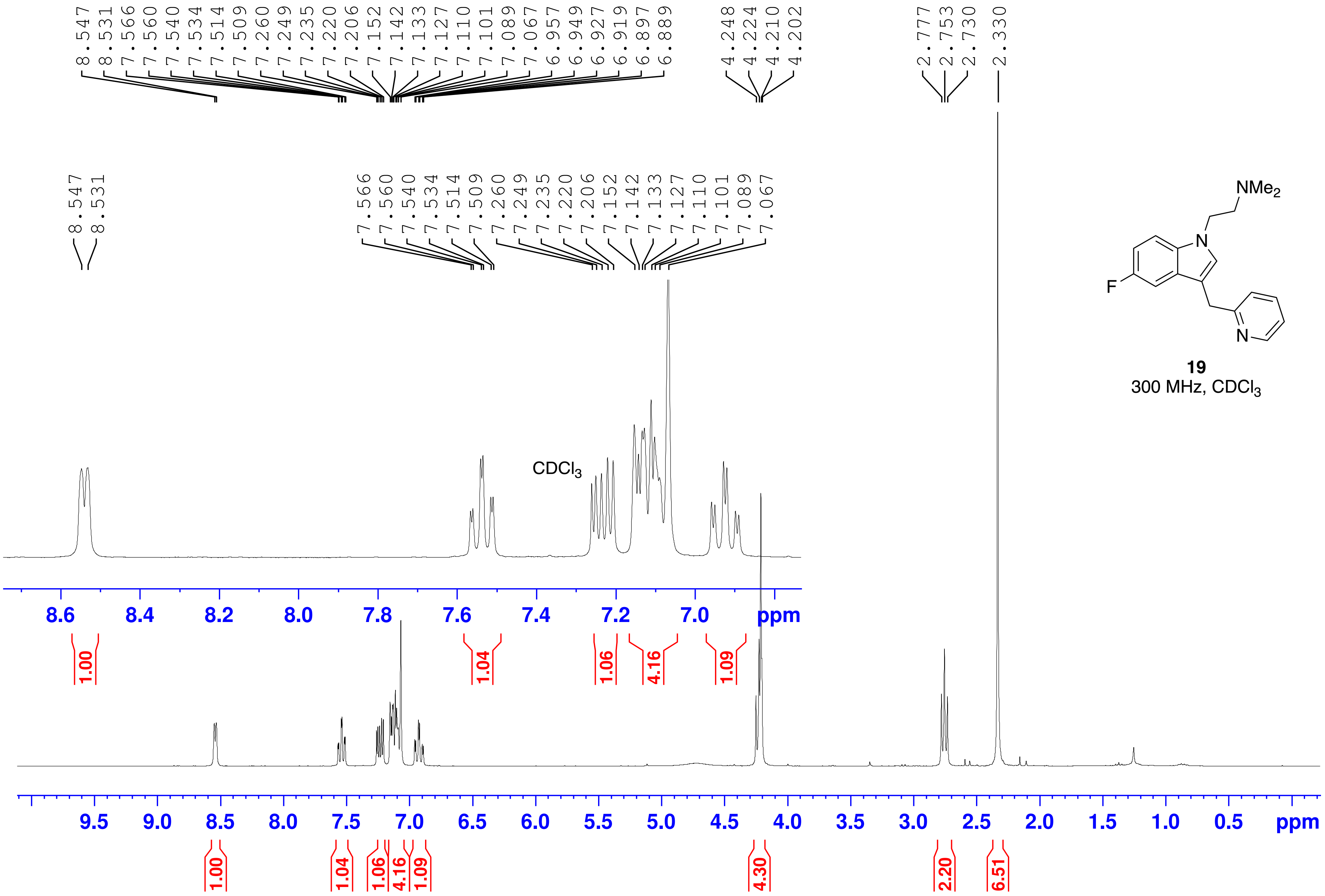

19
300 MHz, $CDCl_3$

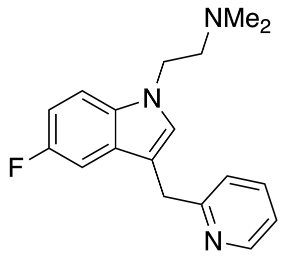

19
75 MHz, $CDCl_3$

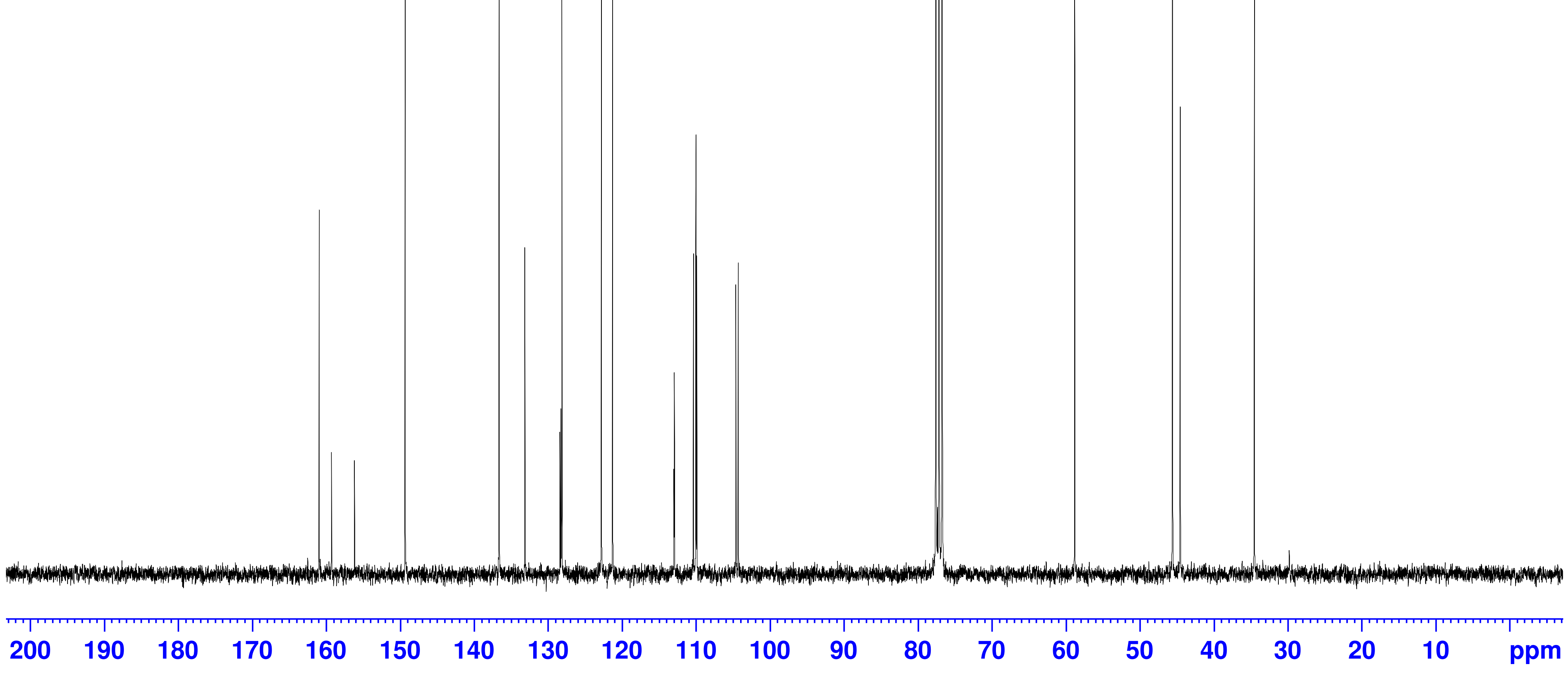

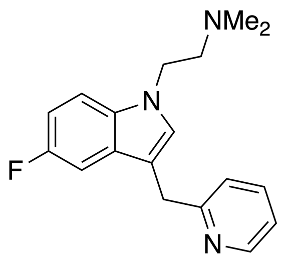

19
282 MHz, $CDCl_3$

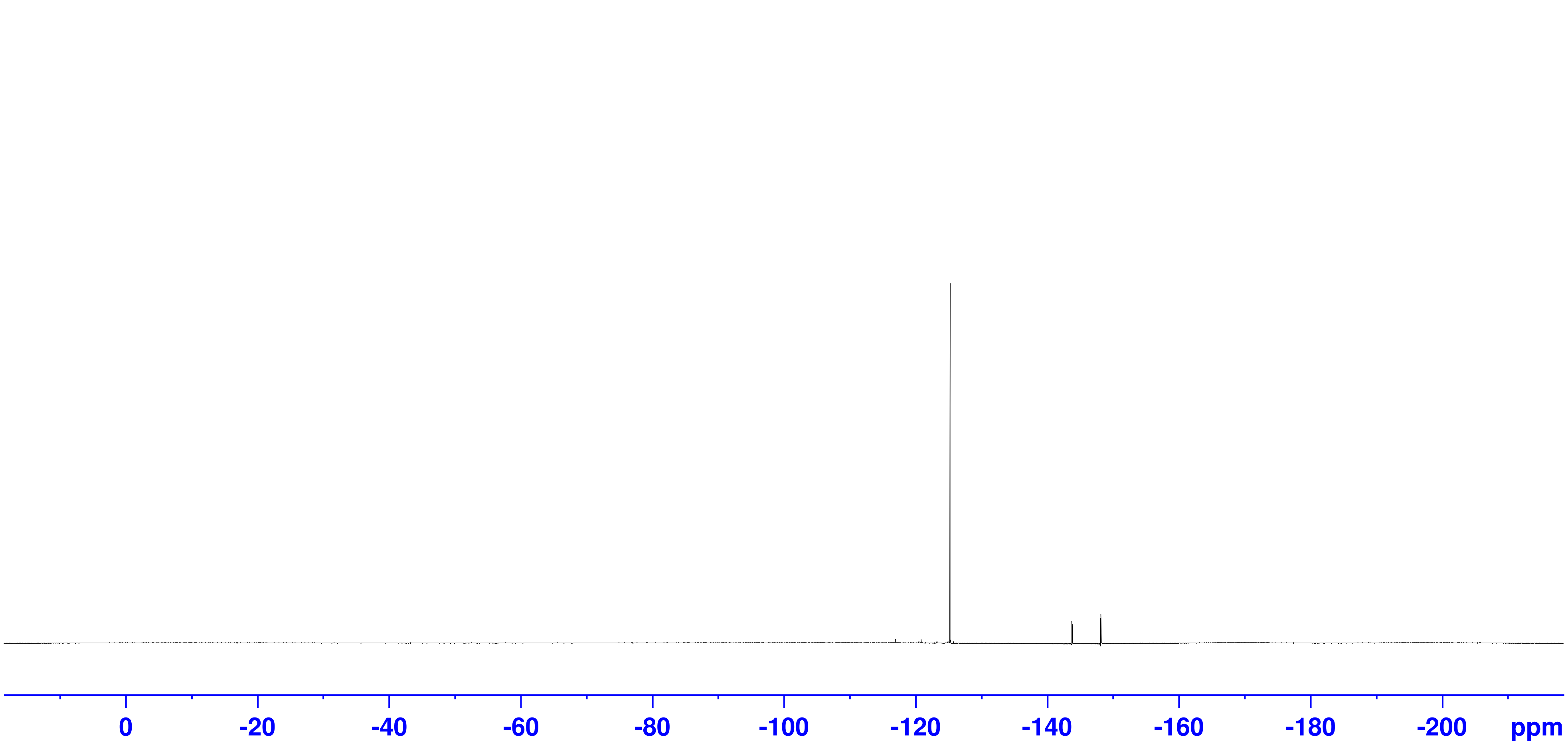

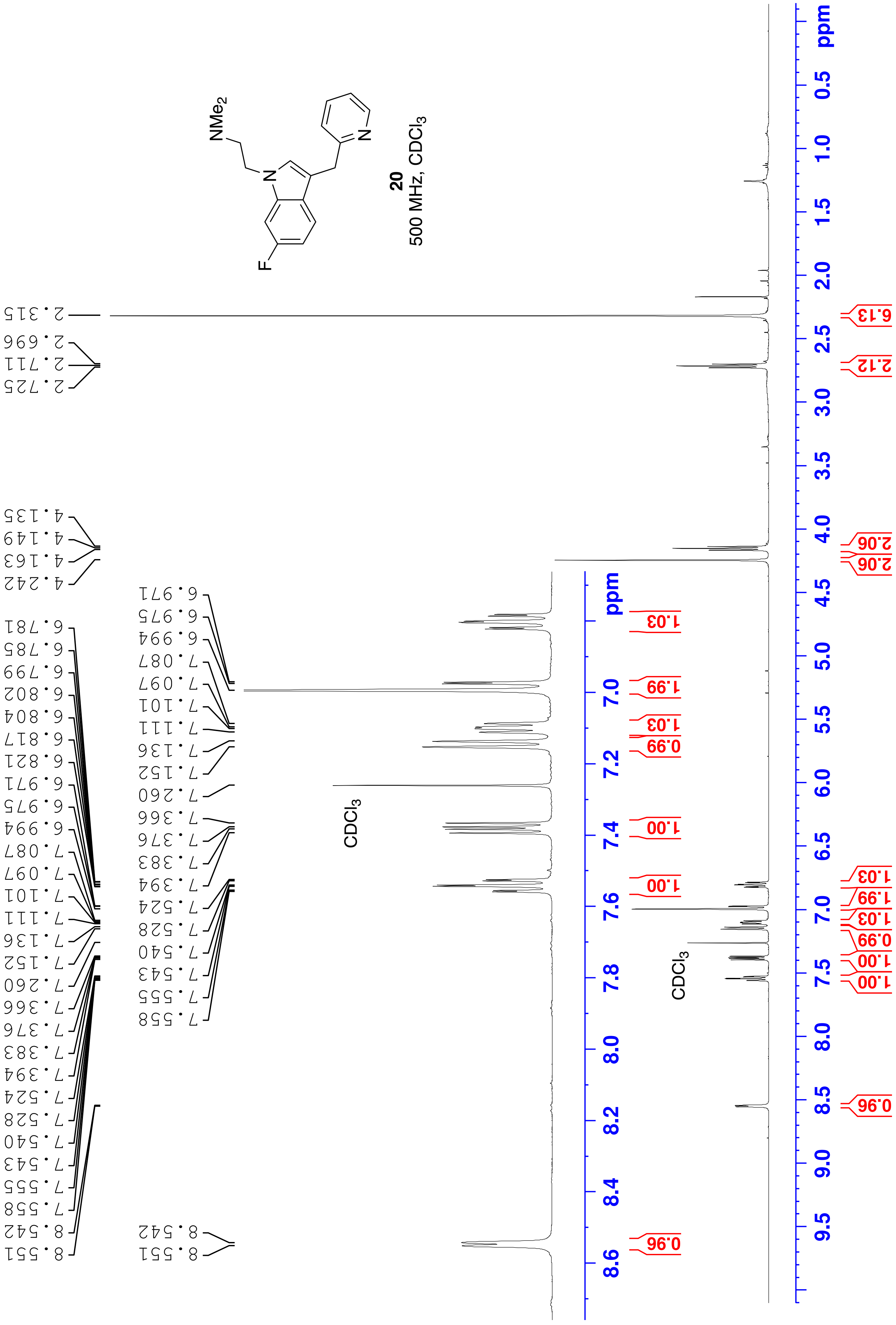
NMe2
N
N
F
20
500 MHz, CDCl3
CDCl3
CDCl3
ppm
ppm

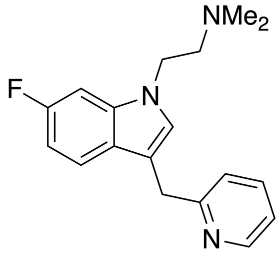

20
125 MHz, $CDCl_3$

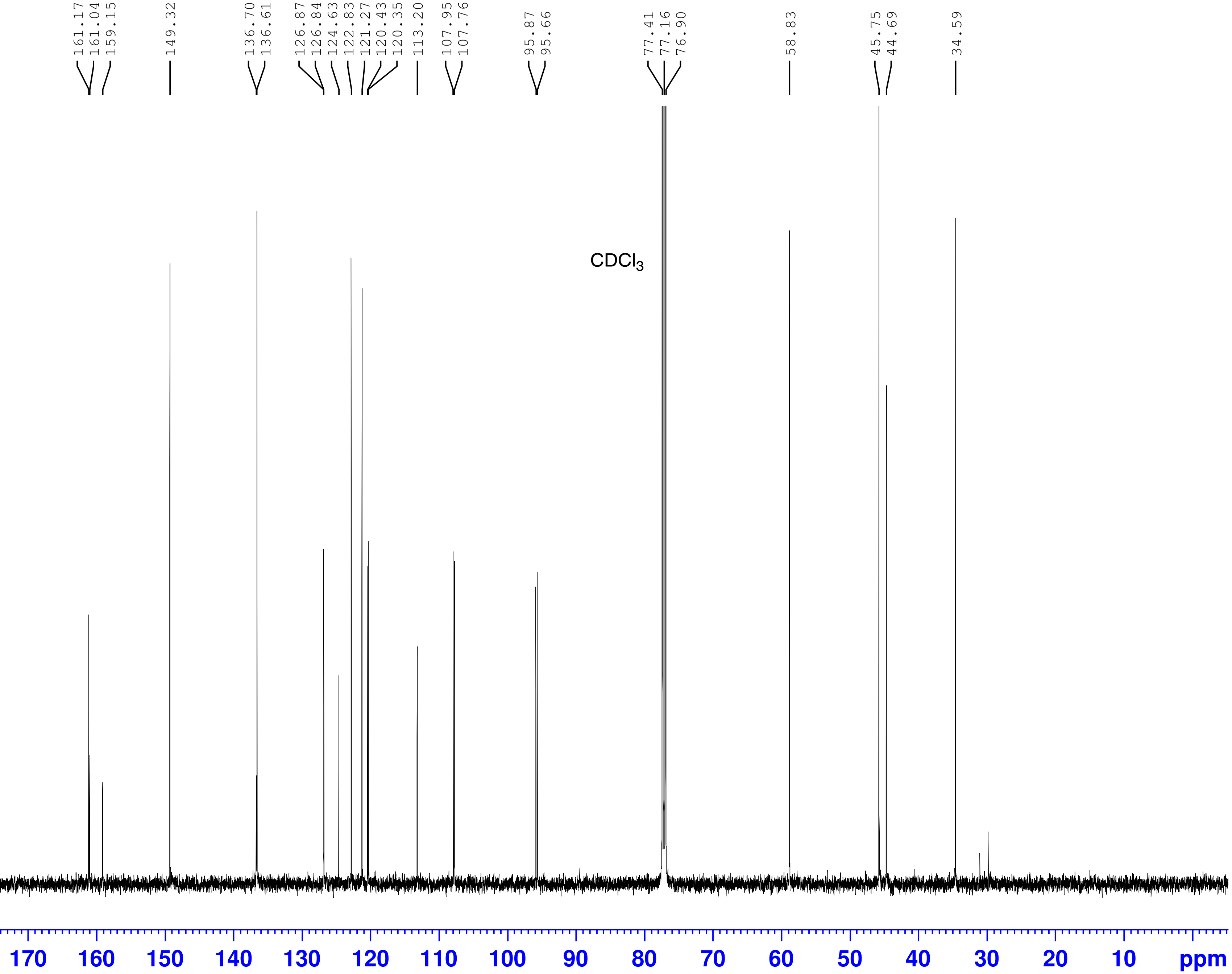

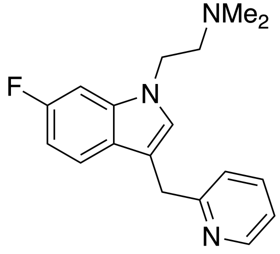

20
282 MHz, $CDCl_3$

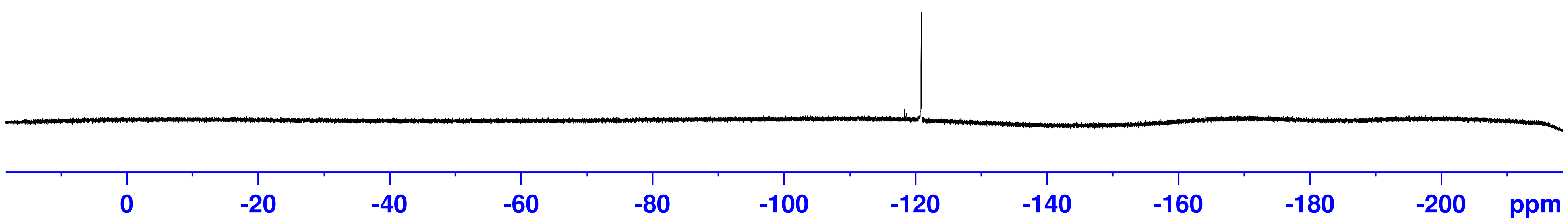

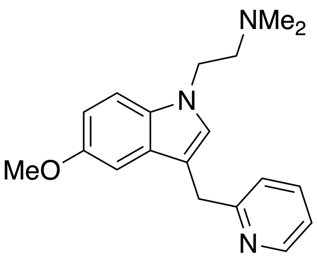

21
300 MHz, $CDCl_3$

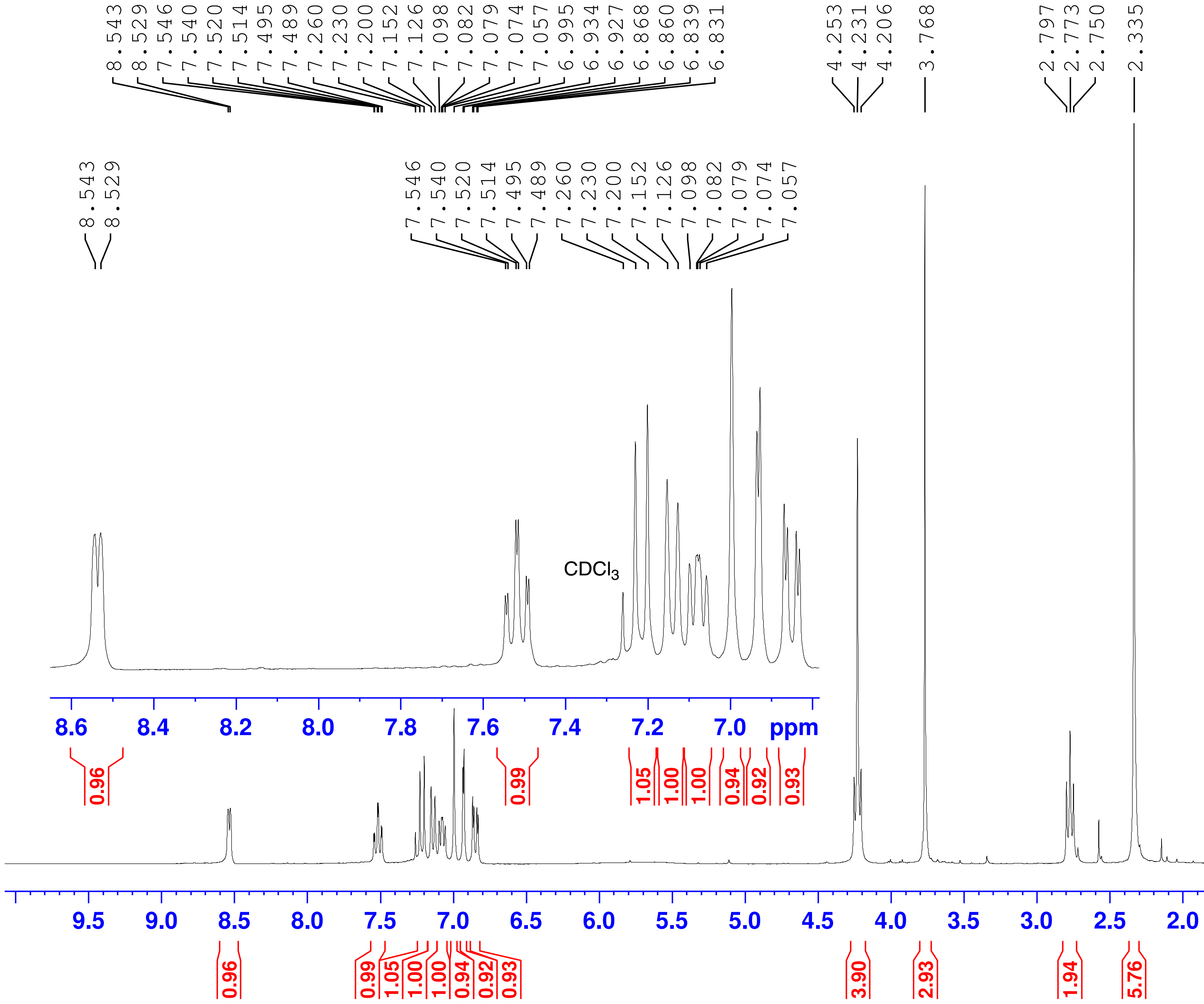

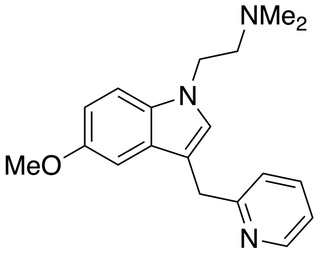

21
75 MHz, $CDCl_3$

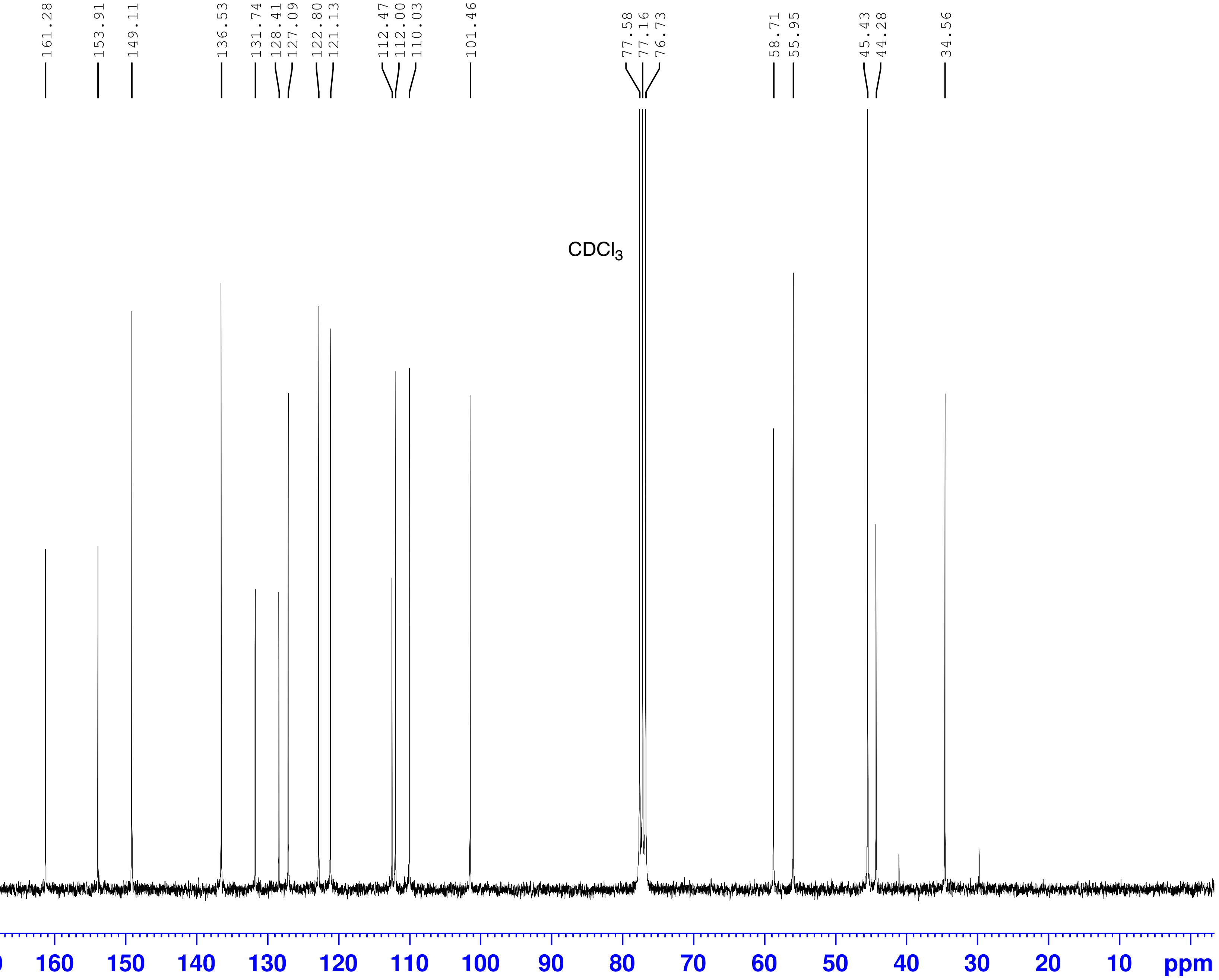

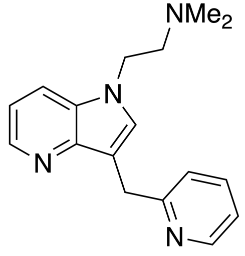

22
400 MHz, $CDCl_3$

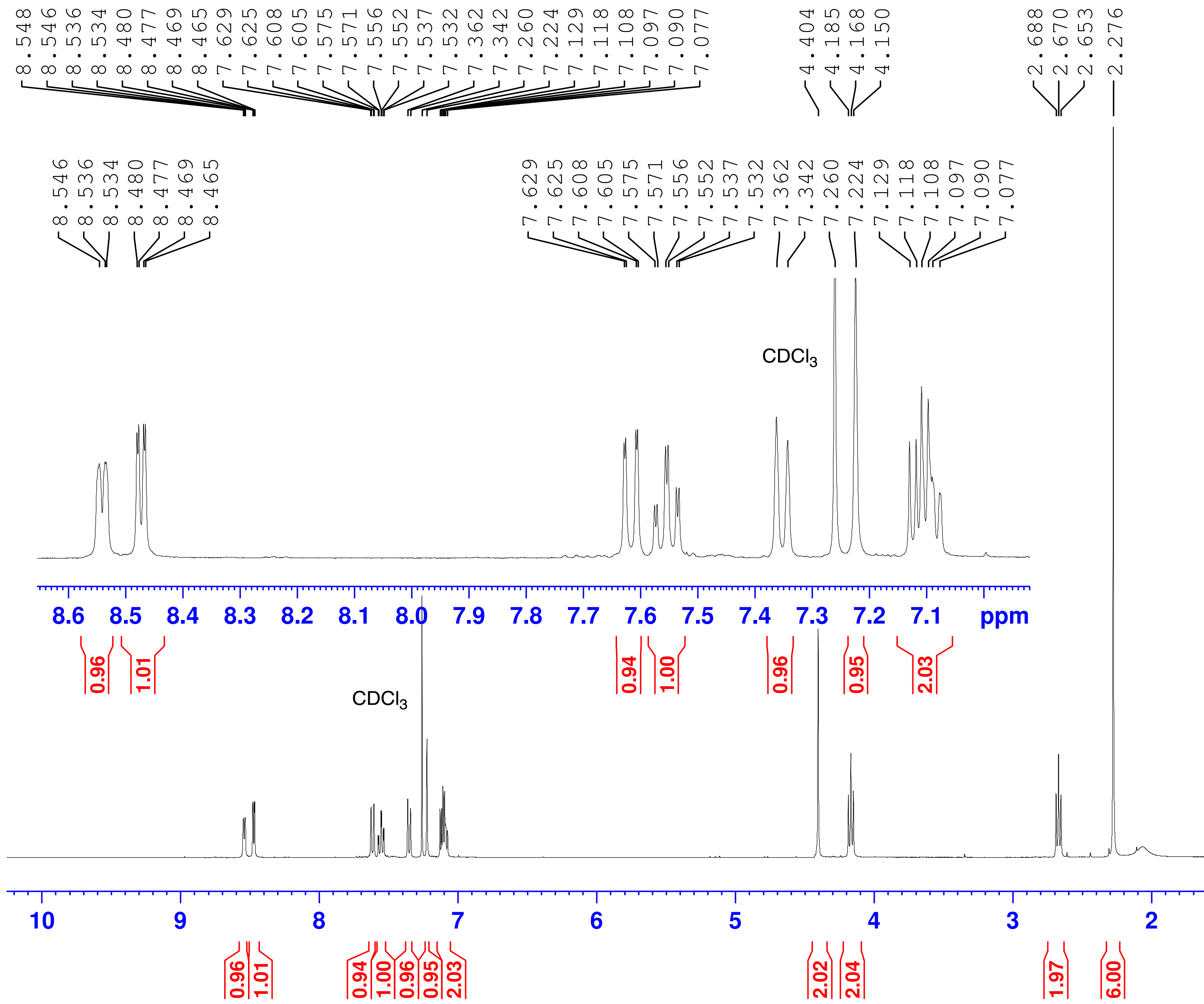

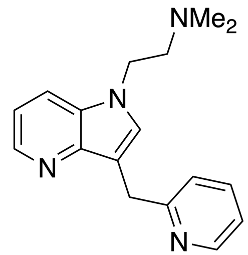

22
100 MHz, $CDCl_3$

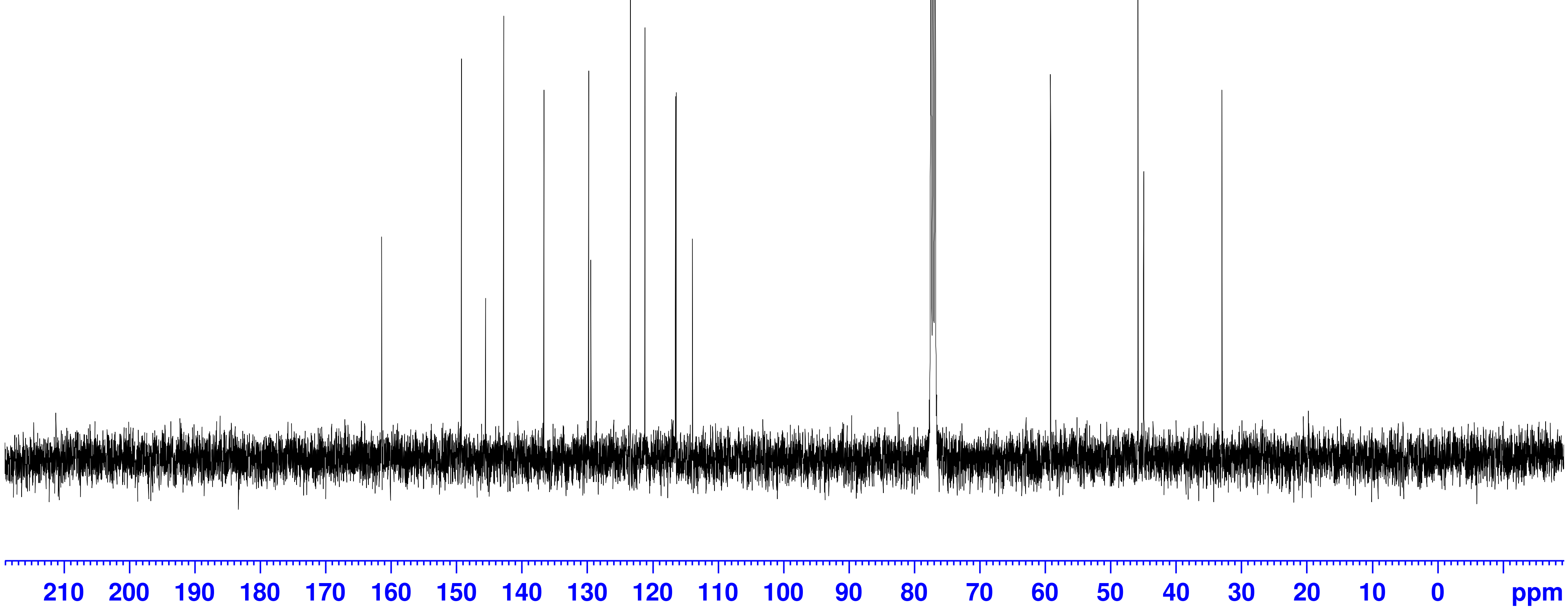

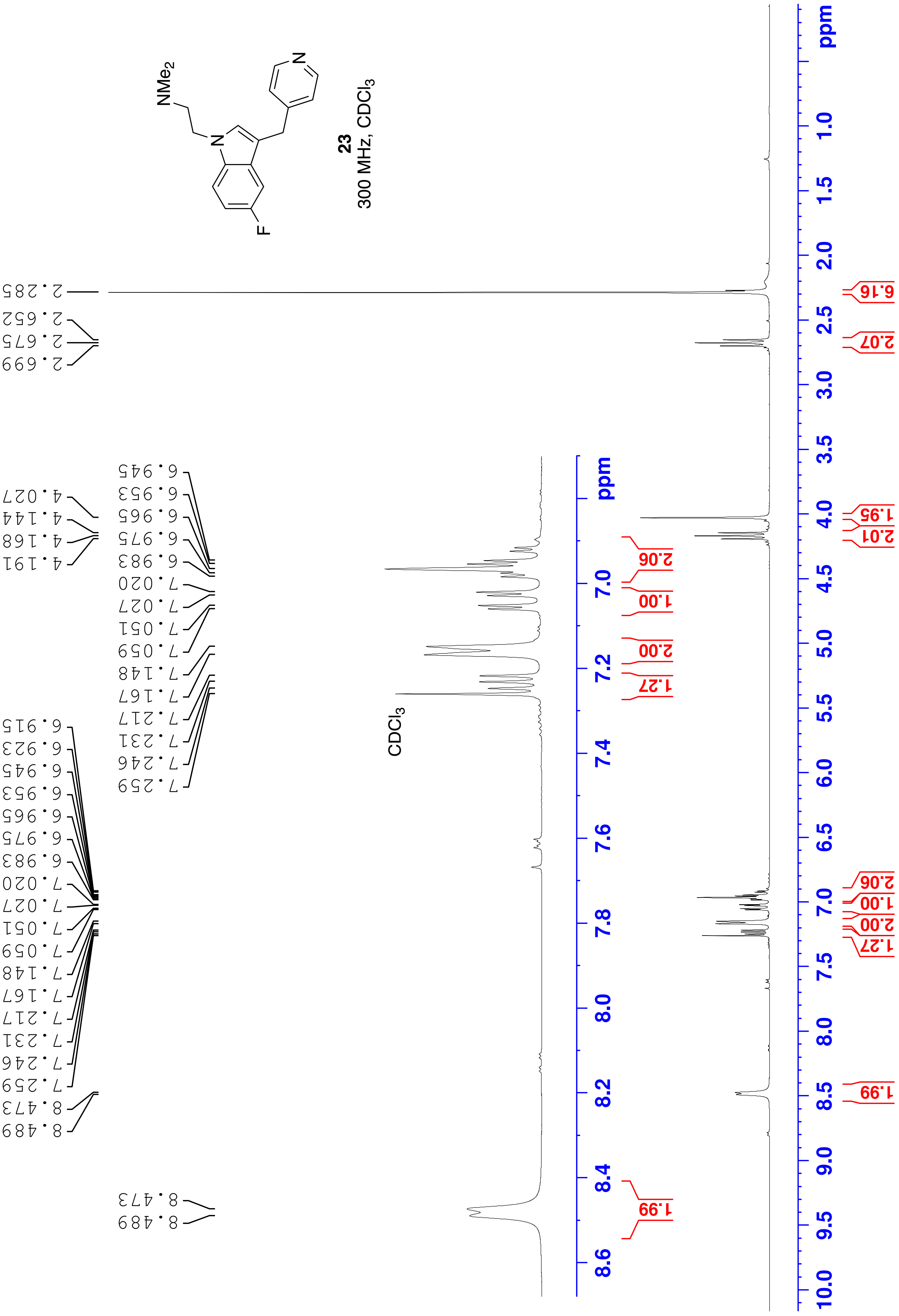

NMe2
N
F
N
23
300 MHz, CDCl3
CDCl3
2.285
2.652
2.675
2.699
4.027
4.144
4.168
4.191
6.915
6.923
6.945
6.953
6.965
6.975
6.983
7.020
7.027
7.051
7.059
7.148
7.167
7.217
7.231
7.246
7.259
8.473
8.489
6.16
2.07
1.95
2.01
2.06
1.00
2.00
1.27
1.99
ppm
1.0
1.5
2.0
2.5
3.0
3.5
4.0
4.5
5.0
5.5
6.0
6.5
7.0
7.5
8.0
8.5
9.0
9.5
10.0
7.0
7.2
7.4
7.6
7.8
8.0
8.2
8.4
8.6

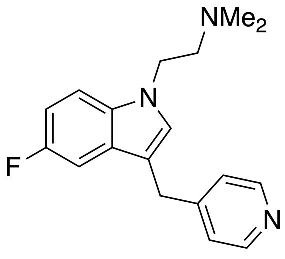

23
75 MHz, $CDCl_3$

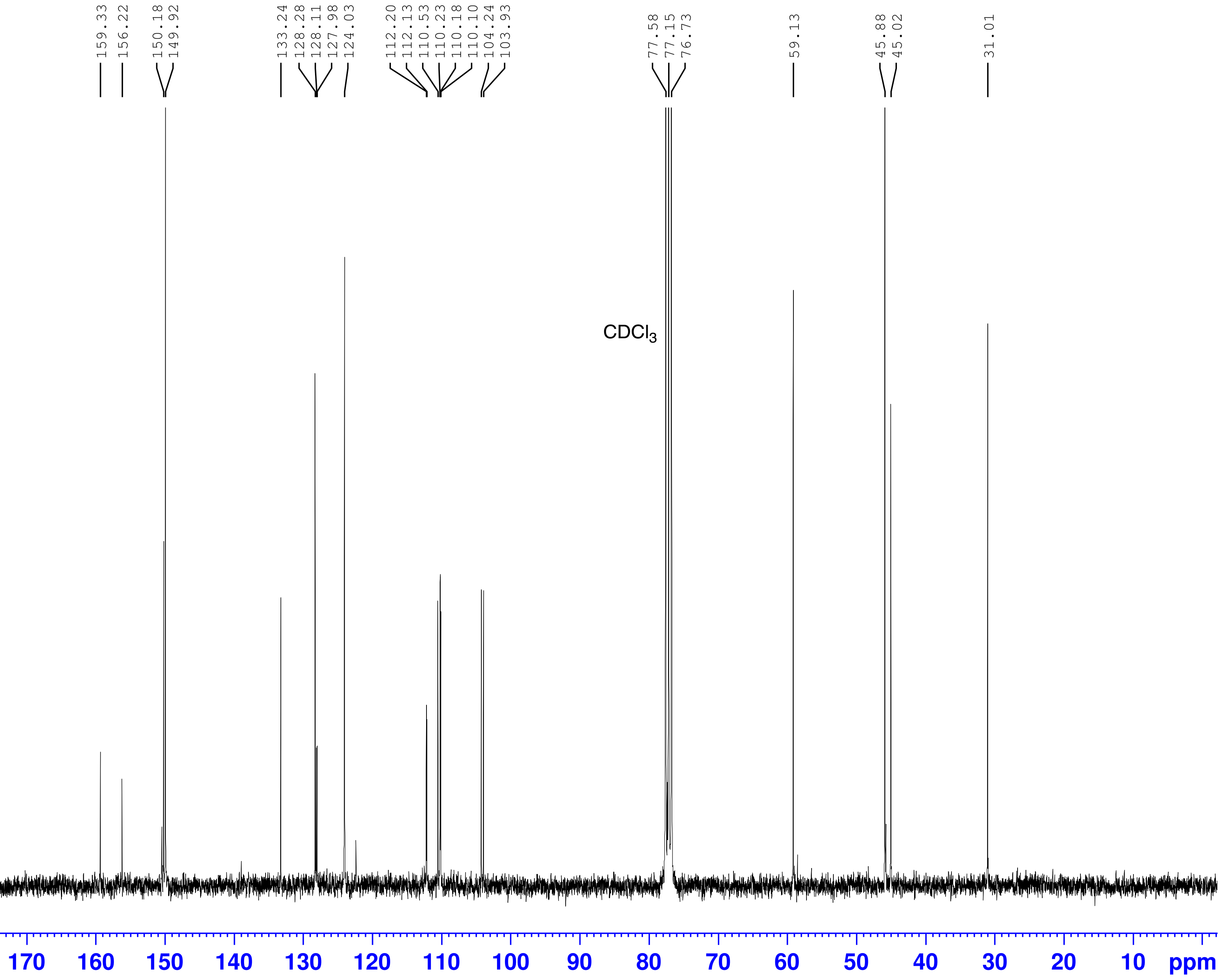

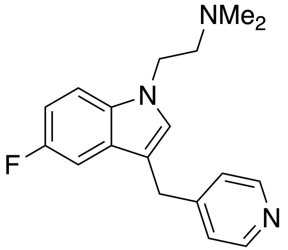

23
282 MHz, $CDCl_3$

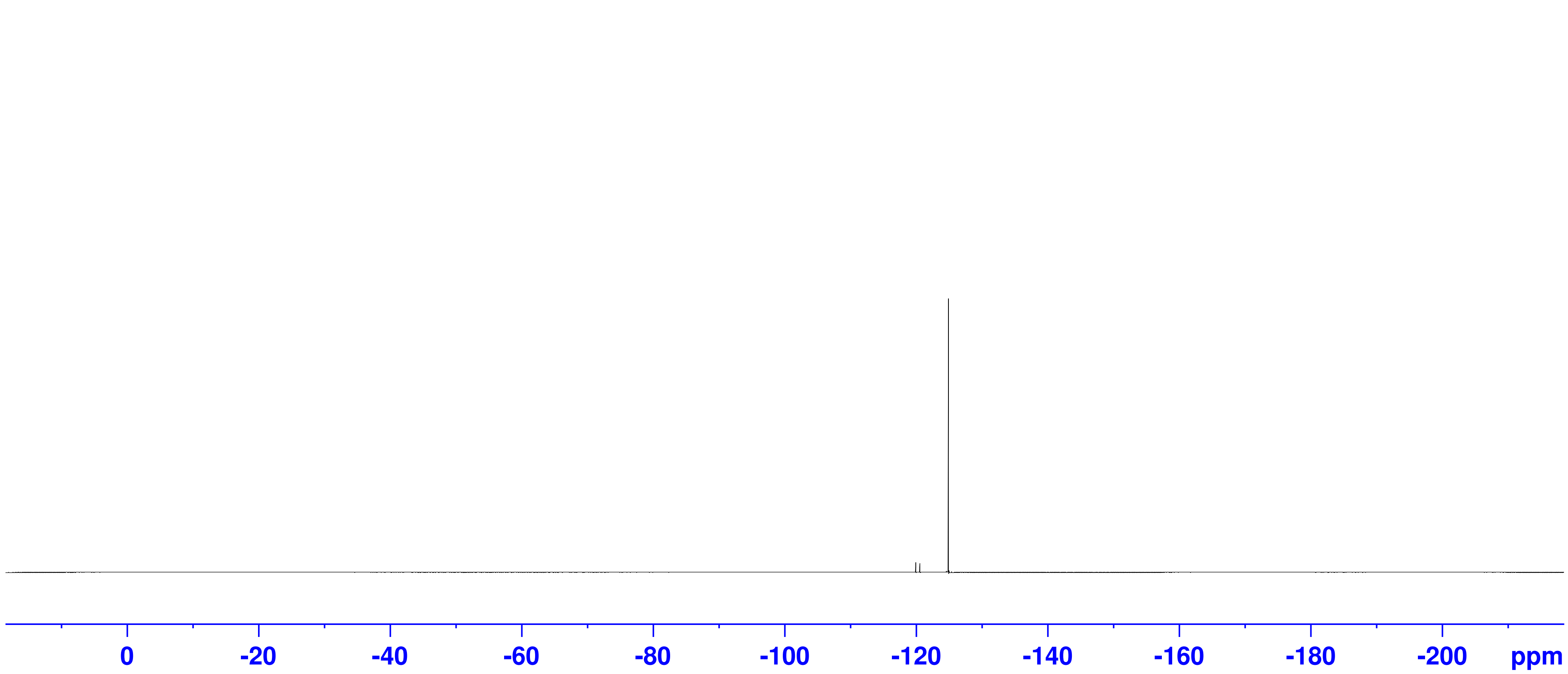

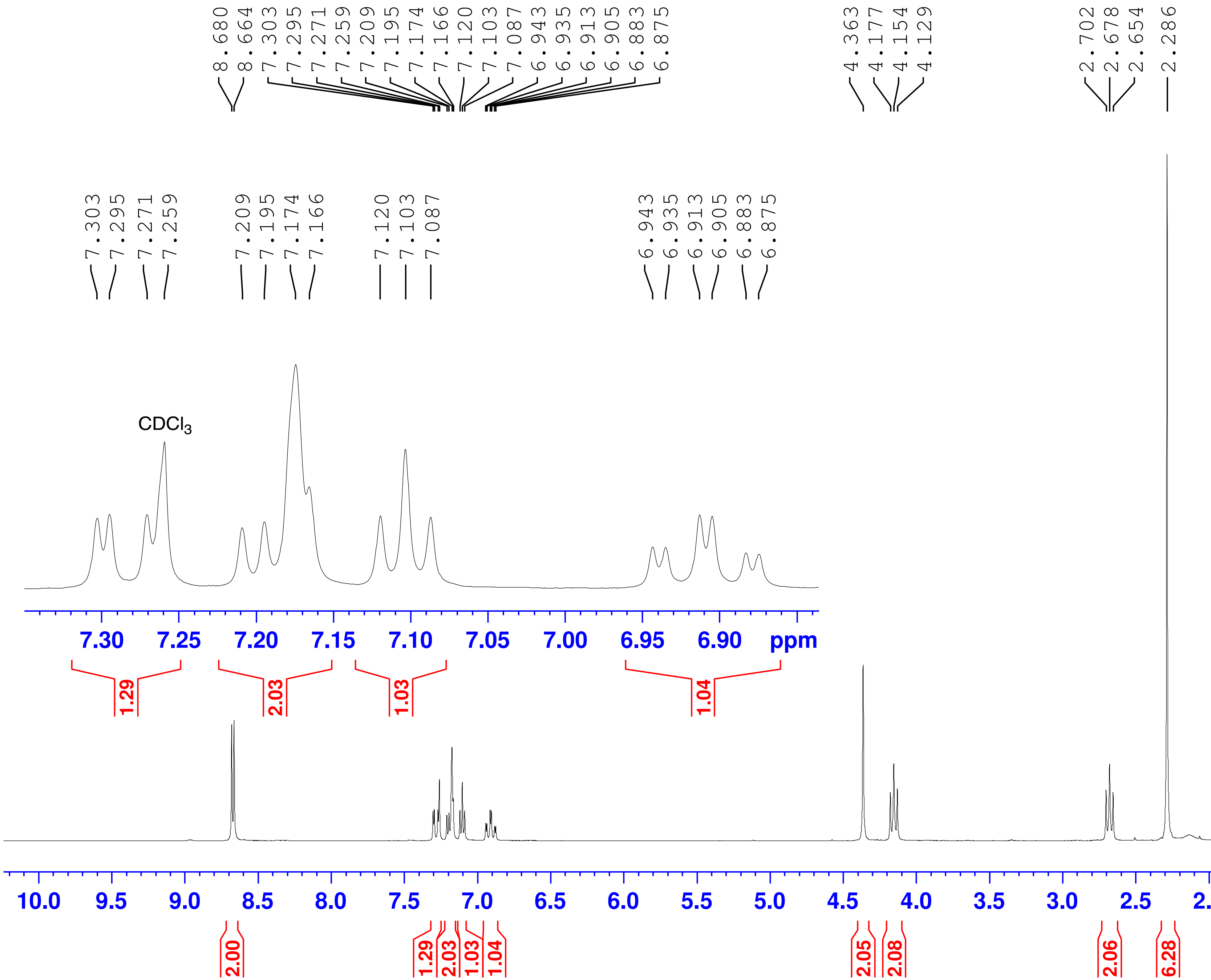

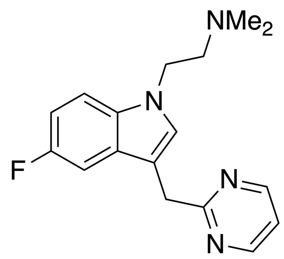

24
300 MHz, $CDCl_3$

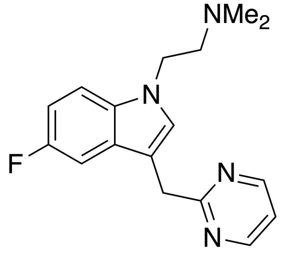

24
75 MHz, $CDCl_3$

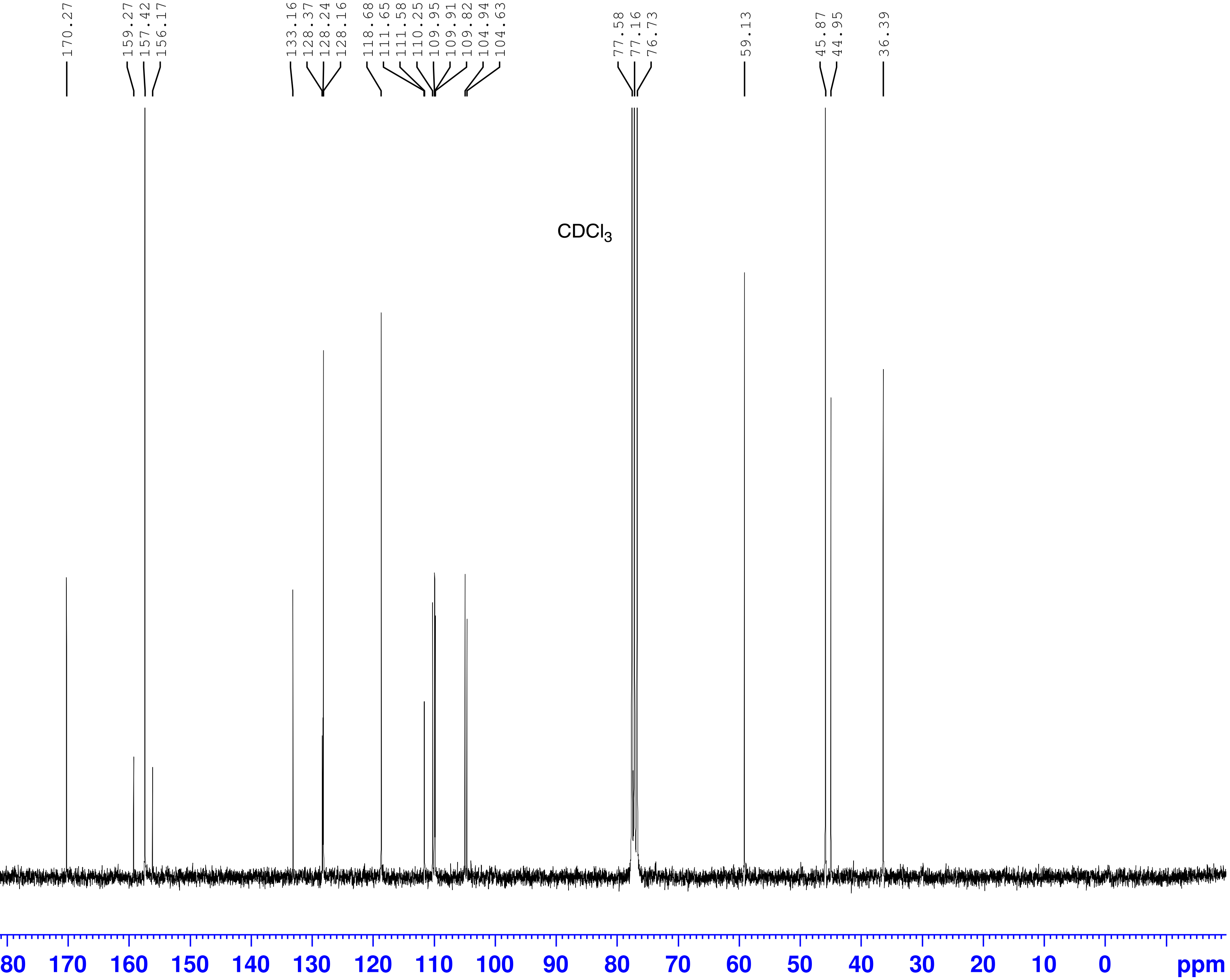

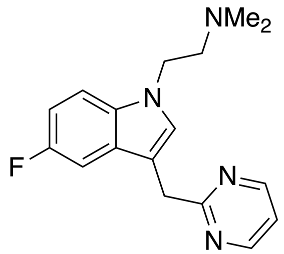

24
282 MHz, $CDCl_3$

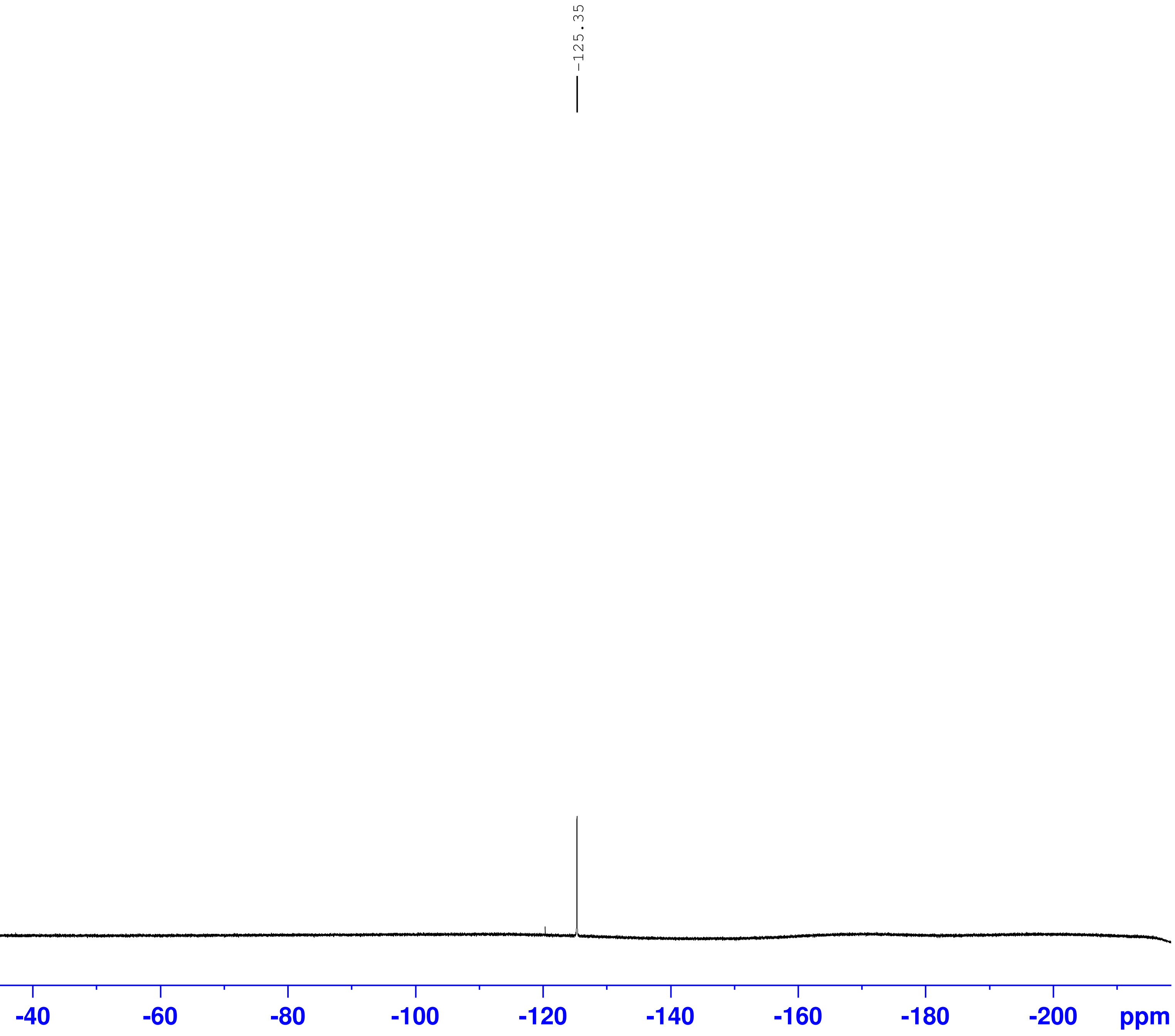

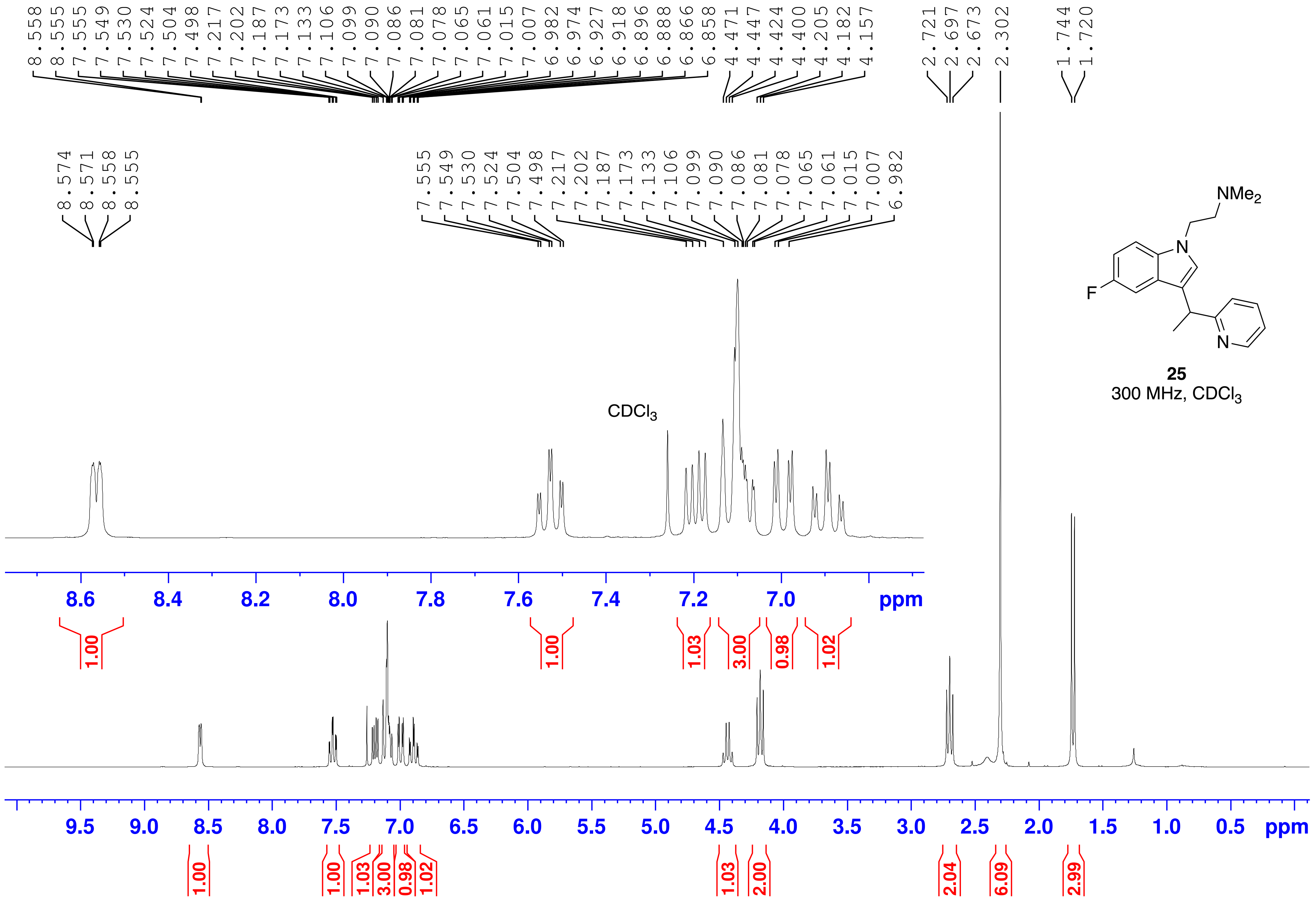

8.558 8.555 7.555 7.549 7.530 7.524 7.504 7.498 7.217 7.202 7.187 7.173 7.133 7.106 7.099 7.090 7.086 7.081 7.078 7.065 7.061 7.015 7.007 6.982 6.974 6.927 6.918 6.896 6.888 6.866 6.858 4.471 4.447 4.424 4.400 4.205 4.182 4.157 2.721 2.697 2.673 2.302 1.744 1.720
8.574 8.571 8.558 8.555
7.555 7.549 7.530 7.524 7.504 7.498 7.217 7.202 7.187 7.173 7.133 7.106 7.099 7.090 7.086 7.081 7.078 7.065 7.061 7.015 7.007 6.982
CDCl3
NMe2
N
F
N
25
300 MHz, CDCl3
8.6 8.4 8.2 8.0 7.8 7.6 7.4 7.2 7.0 ppm
1.00 1.00 1.03 3.00 0.98 1.02
9.5 9.0 8.5 8.0 7.5 7.0 6.5 6.0 5.5 5.0 4.5 4.0 3.5 3.0 2.5 2.0 1.5 1.0 0.5 ppm
1.00 1.00 1.03 3.00 0.98 1.02 1.03 2.00 2.04 6.09 2.99

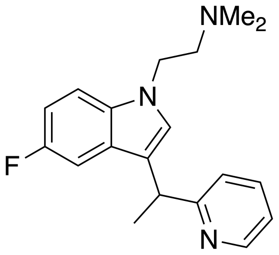

25
75 MHz, $CDCl_3$

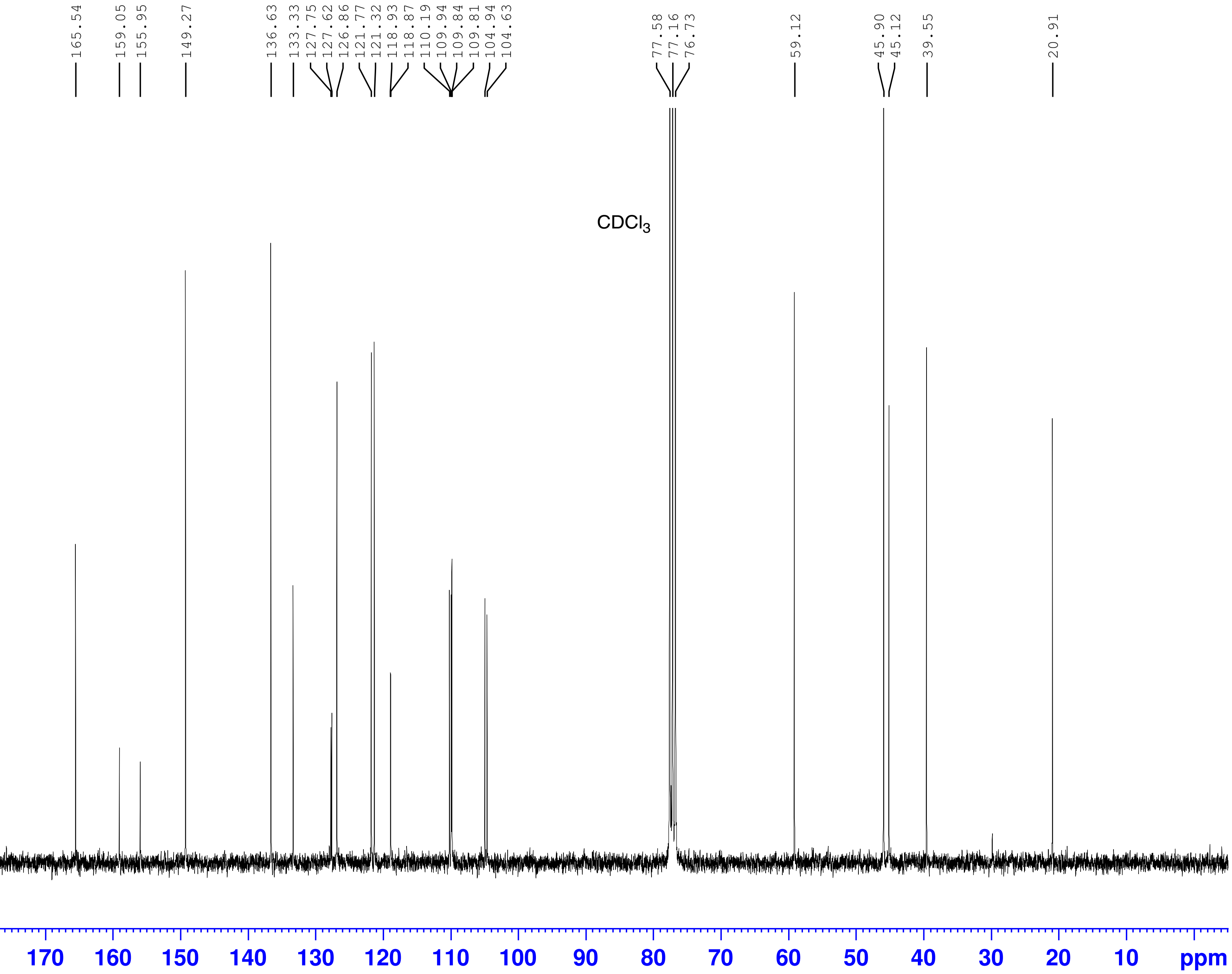

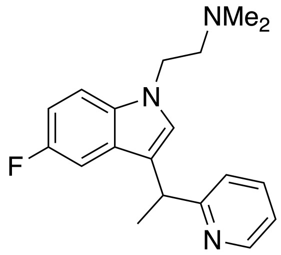

25
282 MHz, $CDCl_3$

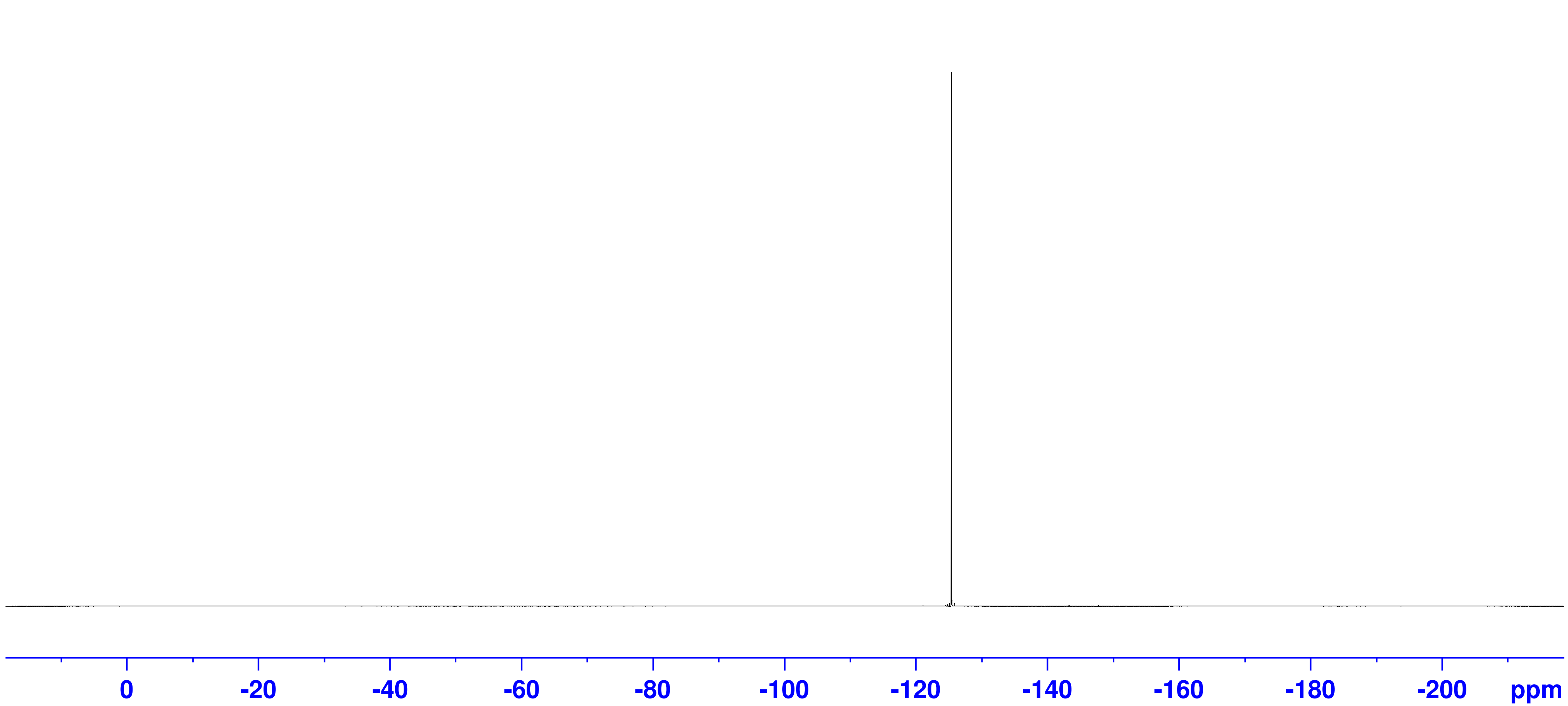

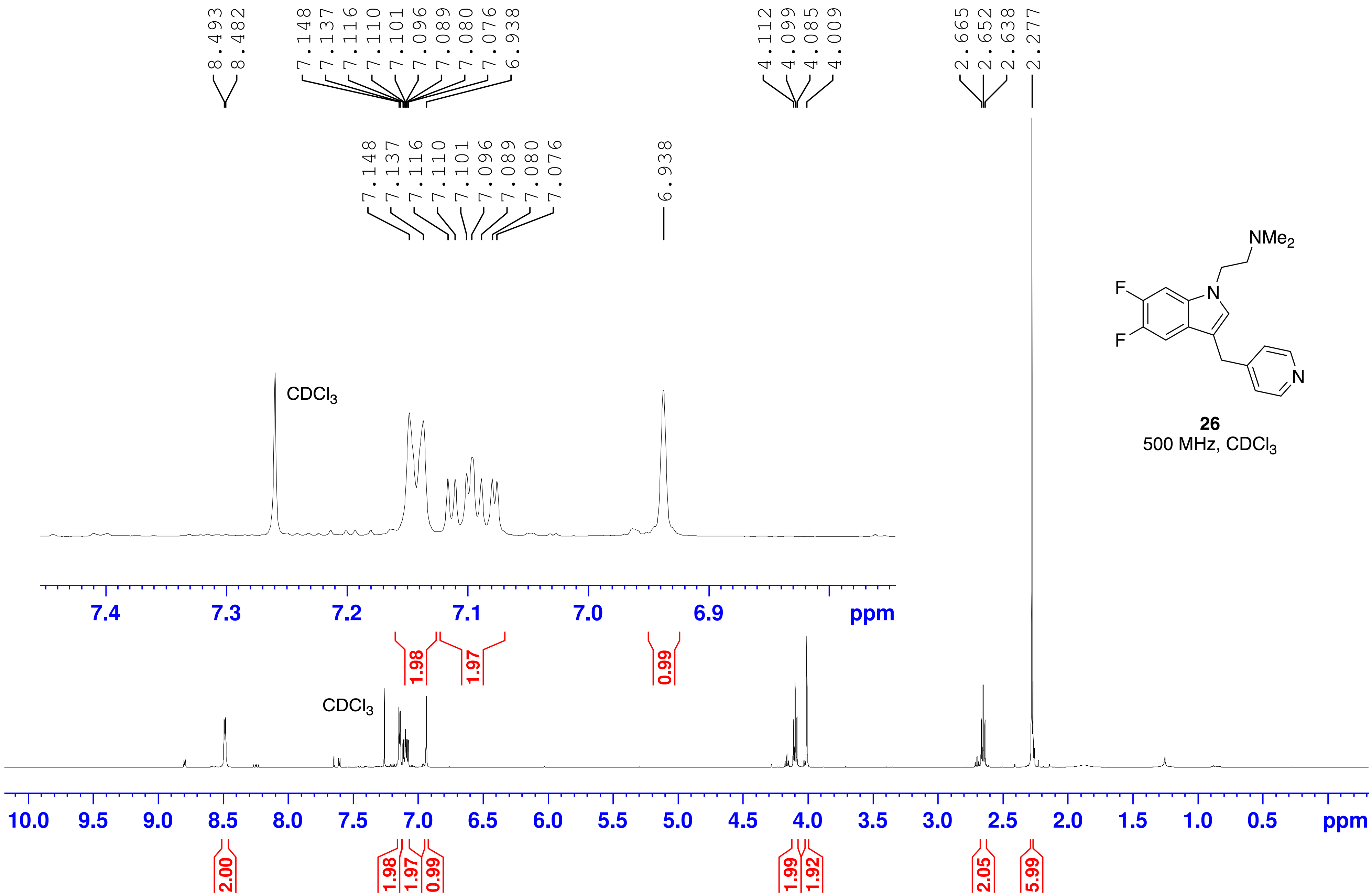
8.493
8.482
7.148
7.137
7.116
7.110
7.101
7.096
7.089
7.080
7.076
6.938
4.112
4.099
4.085
4.009
2.665
2.652
2.638
2.277
NMe2
F
F
N
N
26
500 MHz, CDCl3
CDCl3
7.4
7.3
7.2
7.1
7.0
6.9
ppm
1.98
1.97
0.99
10.0
9.5
9.0
8.5
8.0
7.5
7.0
6.5
6.0
5.5
5.0
4.5
4.0
3.5
3.0
2.5
2.0
1.5
1.0
0.5
2.00
1.99
1.92
2.05
5.99

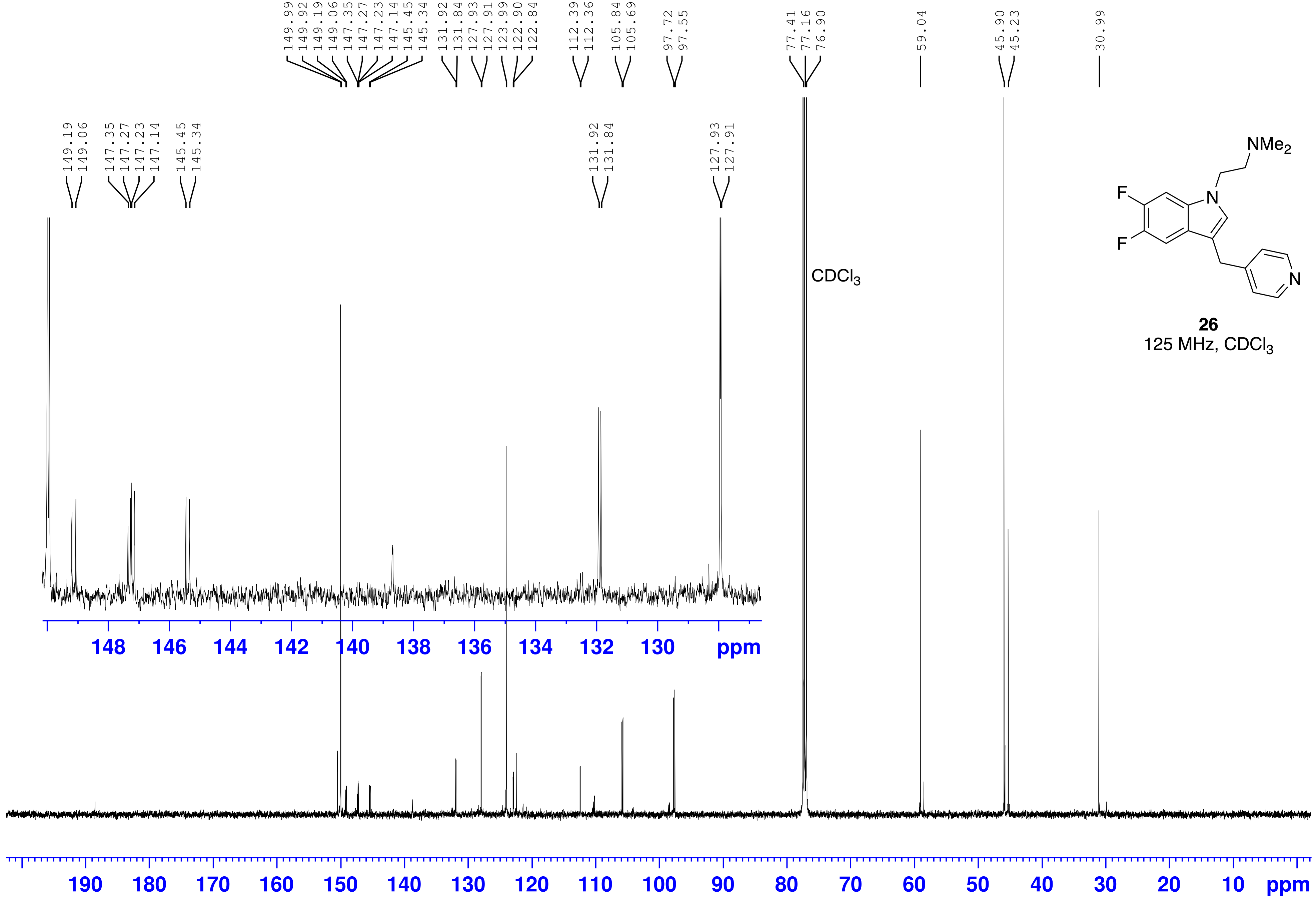
149.99
149.92
149.19
149.06
147.35
147.27
147.23
147.14
145.45
145.34
131.92
131.84
127.93
127.91
123.99
122.90
122.84
112.39
112.36
105.84
105.69
97.72
97.55
77.41
77.16
76.90
59.04
45.90
45.23
30.99
CDCl3
NMe2
F
F
N
N
26
125 MHz, CDCl3
ppm

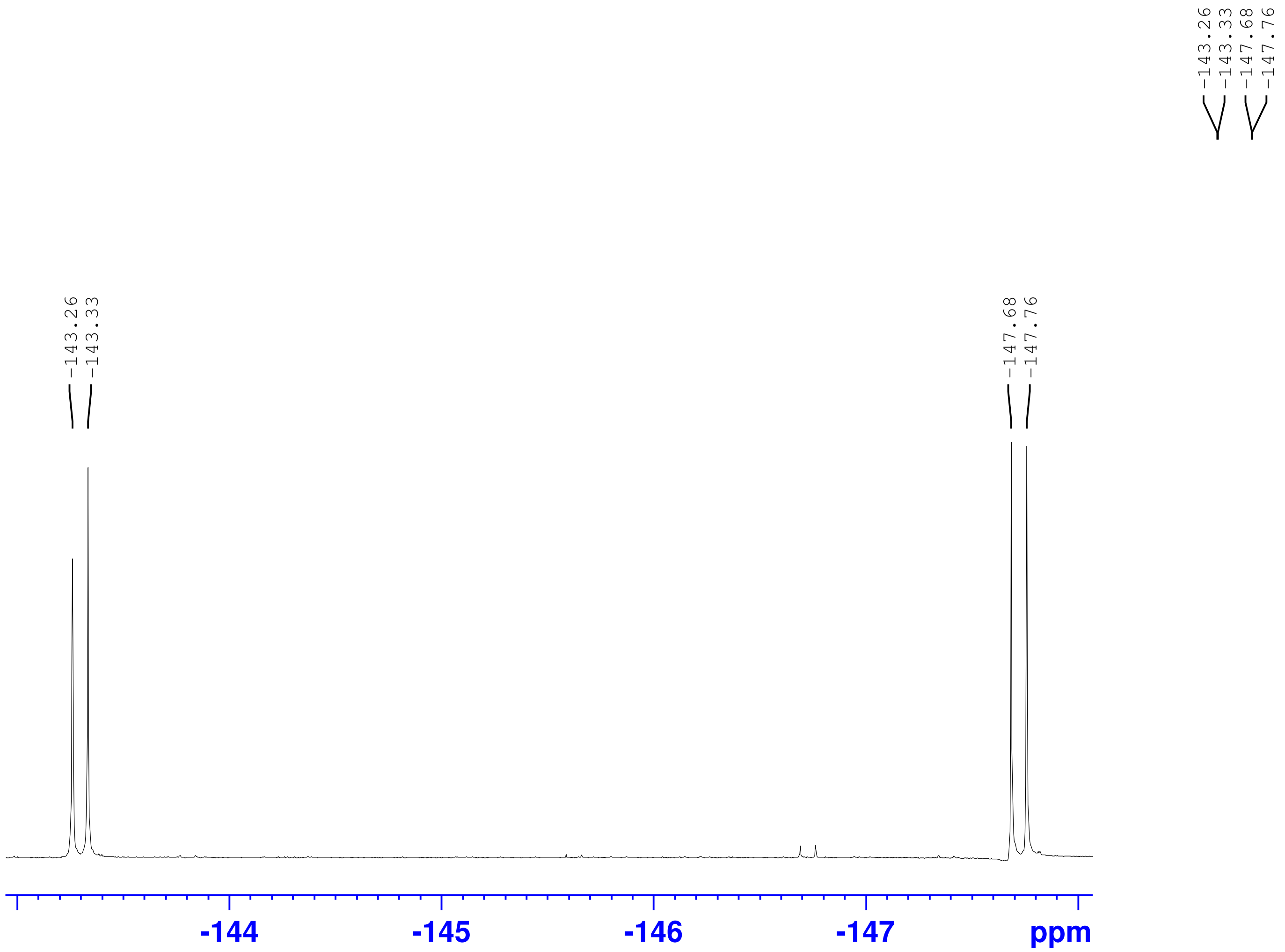

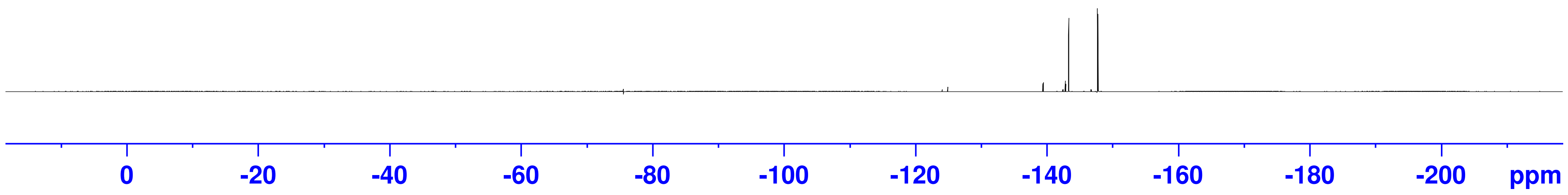

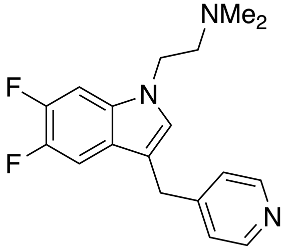

26
282 MHz, $CDCl_3$

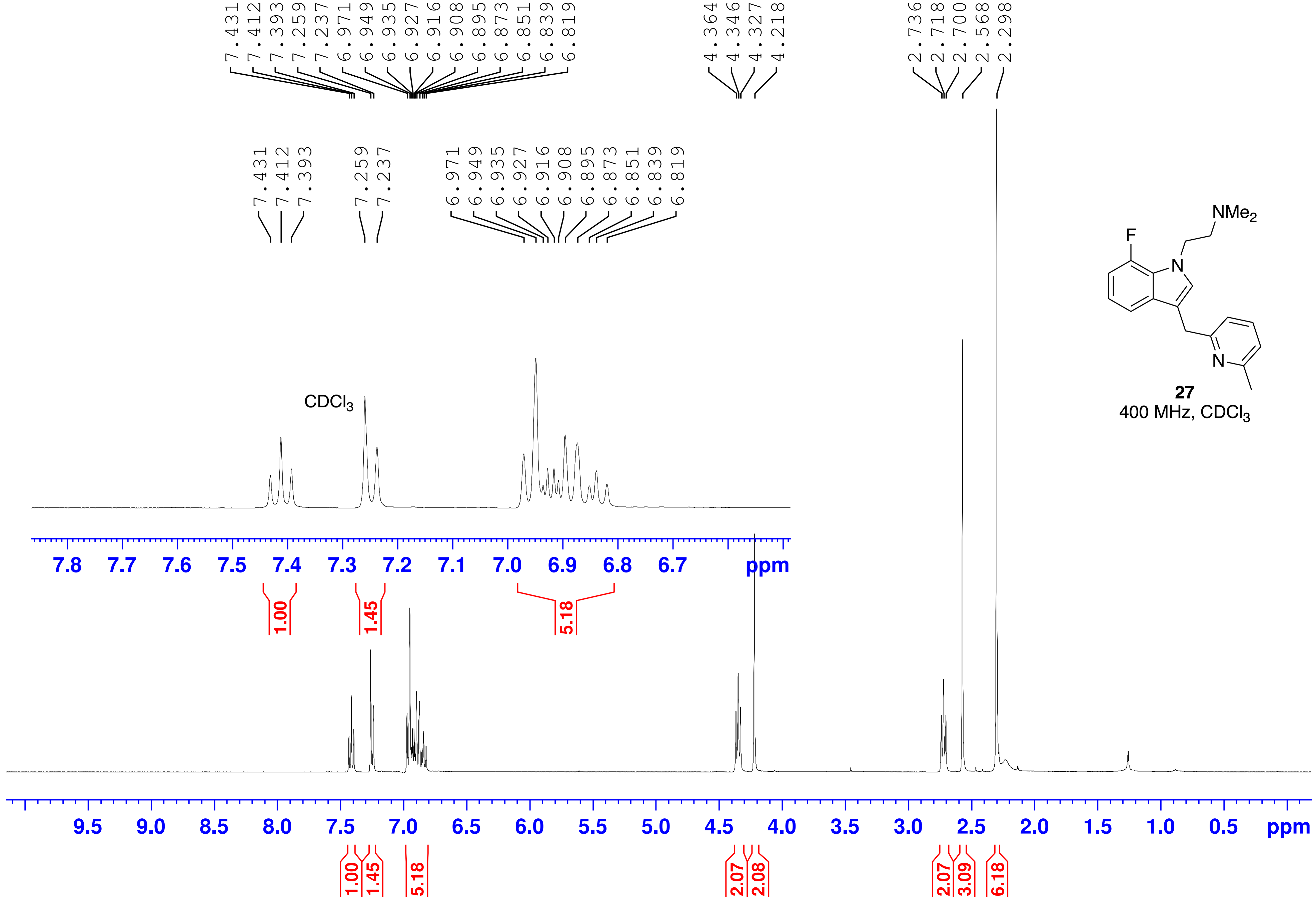

27
400 MHz, $CDCl_3$

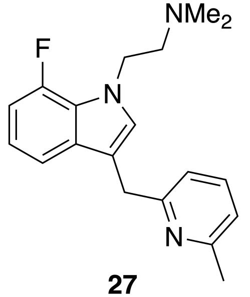

27
100 MHz, $CDCl_3$

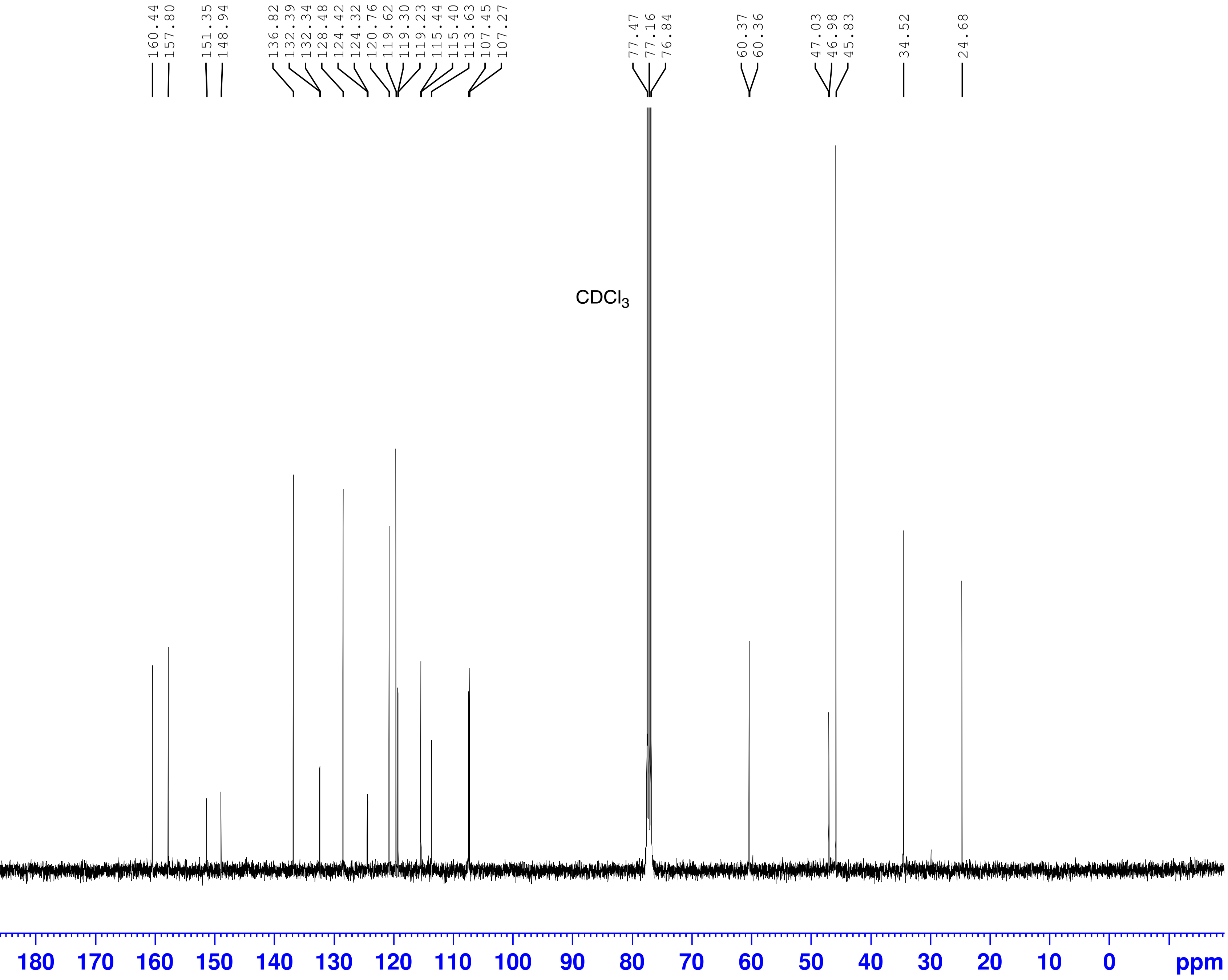

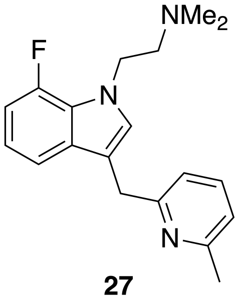

27
282 MHz, $CDCl_3$

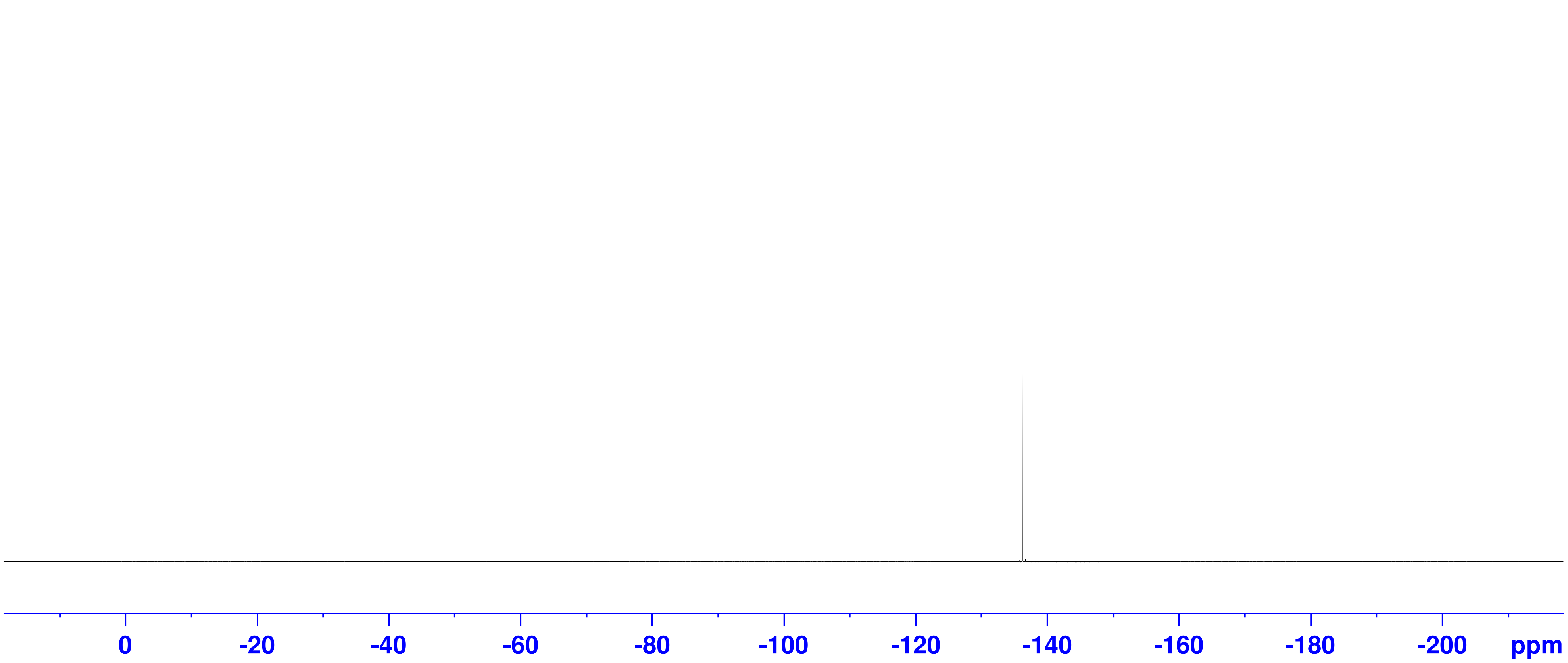

Supplement: Supplementary file 1 — ml4c00130_si_001.pdf [file ml4c00130_si_001.pdf]
